# Isolation and characterization of *Candida metapsilosis* from foci of chronic pododermatitis in captive steppe eagles in Kazakhstan

**DOI:** 10.1371/journal.pone.0353552

**Published:** 2026-07-31

**Authors:** Elena Kukhar, Gulshat Bailina, Aziza Nessipbayeva, Rano Sattarova, Marat Turkeyev

**Affiliations:** 1 S. Seifullin Kazakh Agro Technical Research University, Astana, Kazakhstan; 2 Kazakh Scientific Research Veterinary Institute LLP, Almaty, Kazakhstan; Charles University: Univerzita Karlova, CZECHIA

## Abstract

Pododermatitis (bumblefoot) is a chronic, debilitating disease of the plantar surface of the foot that affects birds of prey kept in captivity worldwide. Although bacterial pathogens, especially *Staphylococcus aureus*, are most commonly considered as causative agents, the contribution of opportunistic yeasts to chronic, non-healing footpad lesions remains poorly characterized. Keratinophilic yeasts may sustain the disease process by degrading keratin in superficial tissues, impairing wound healing and, owing to their thermotolerance and minimal nutritional requirements, persisting in the environment of the bird’s enclosure. In this study, three captive steppe eagles (*Aquila nipalensis*) from a single aviary in Kazakhstan, all presenting with chronic pododermatitis unresponsive to antibacterial treatment, were investigated by integrated mycological, biochemical and molecular approaches. The yeast isolates were recovered from the deep footpad lesions and identified to species level by sequencing of the ITS1-5.8S-ITS2 rDNA region. All these isolates were assigned to *Candida metapsilosis*, and phylogenetic analysis confirmed their close clustering with reference *C. metapsilosis* sequences. Phenotypic characterization showed that all isolates were thermotolerant (growth at 8–37 °C), expressed strong urease and keratinolytic activity (the latter confirmed *in vitro* by the hair perforation test), high saccharolytic activity and selective, weak proteolytic activity. Disk diffusion screening showed susceptibility to azoles (ketoconazole, clotrimazole, fluconazole) and reduced susceptibility to polyenes (nystatin, amphotericin B). To our knowledge, this is the first report of *C. metapsilosis* isolated from chronic pododermatitis lesions in captive steppe eagles. Combined with the documented *in vitro* virulence-associated traits and the resolution of the lesions following targeted antifungal therapy, our findings support a contributory etiological role of *C. metapsilosis* as an opportunistic pathogen in raptor pododermatitis in immunocompromised birds maintained under suboptimal husbandry. Mycological work-up, including molecular identification, is therefore warranted in cases of chronic, non-resolving pododermatitis in captive birds of prey.

## Introduction

Pododermatitis (also known as bumblefoot) is a common disease of the plantar surface of the foot in captive birds of prey, affecting the metatarsal footpad [1–5]. The condition has been described in detail in falcons used for falconry in Abu Dhabi, United Arab Emirates [6,7], and has also been reported in owls, eagles, geese, ospreys, penguins and swans [8–12]. Possible causes of pododermatitis include poor housing conditions (unsuitable perches), excessive weight and lack of physical activity [3,13], poor hygiene and uncleanliness [5,14,15]. As a rule, footpad dermatitis (FPD) in captive birds occurs when the integrity of the skin is compromised and inflammatory processes develop on the plantar surface of the foot [16]. A distinctive feature of the disease is the chronic process of “necrosis” of the epithelium of the plantar surface of the foot, followed by the penetration of pathogens and deep inflammation of the tissues, leading to their destruction and complete loss of foot function [4,17]. The main infectious agent of footpad dermatitis (FPD) is usually *Staphylococcus aureus* [17]. However, other bacterial pathogens of avian pododermatitis are known, such as *E. coli*, *Pasteurella* spp., *Klebsiella* spp. and several others [12]. The isolation of *Candida* spp. microscopic fungi from affected areas has been reported [15]. Fungal diseases in birds caused by opportunistic yeast fungi of the genus *Candida*, especially *C. albicans*, pose a serious threat to domestic and wild birds, both captive and free ranging [18]. Literature sources report a case of systemic candidiasis caused by *Candida parapsilosis* (to which *C. metapsilosis* belongs) in a 20-year-old blue-fronted Amazon parrot. The main clinical symptom in this bird was lameness [19]. There is no information on the role of *C. metapsilosis* in the development of diseases in wild birds kept in captivity, including candidal pododermatitis, and the biological properties of the pathogens have not been described [20].

The aim of the present study was to characterize, using combined morphological, biochemical, molecular and phylogenetic approaches, yeast isolates recovered from chronic, non-healing footpad lesions of captive steppe eagles in Kazakhstan, and to evaluate their potential contribution to the etiology of pododermatitis in these birds.

## Materials and methods

### Animals and clinical history

The Mycology and Fungal Biotechnology Laboratory at the S. Seifullin Kazakh Agrotechnical Research University received biomaterial from wild eagles kept in captivity and samples of bedding from the enclosure where they were kept, with complaints of chronic pododermatitis that did not respond to treatment an antimicrobial combination drug of dioxomethyltetrahydropyrimidine (methyluracil) and chloramphenicol in the form of an ointment called Levomecol, followed by bandaging.

Steppe eagle No. 1 (“Stary”), an adult male of approximately 40 years of age, had been used for many years for commercial photography at the year-round Shymbulak Mountain resort in Almaty. The bird was brought to the zoo by members of the public in 1994 and was presented to the veterinary clinic in the autumn of 2023; the initial clinical signs and behavioral disturbances had been first noted in October 2023, and the bird had been suffering from chronic pododermatitis for more than three years prior to presentation.

Steppe eagle No. 2 (“Orlik”), hatched in 2018, had been received from the public in 2019. Prior to admission to the zoo, the bird had been used for commercial photography in the Kolsai Kolderi State National Nature Park (Almaty region). Veterinary care was first sought in December 2023; at presentation the bird had a clinical history of pododermatitis of 2–3 years.

**Fig 1:**
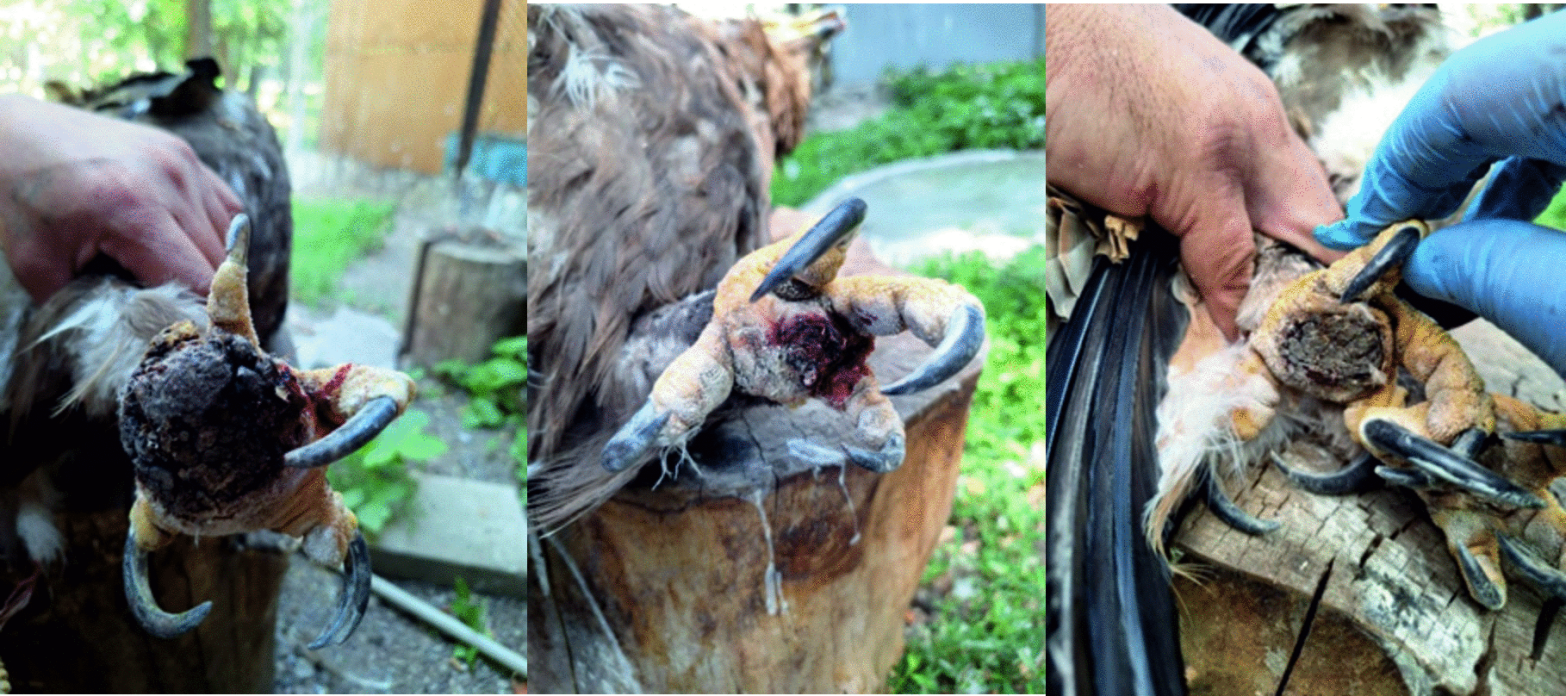
External appearance of lesions on the footpads of steppe eagles.

Steppe eagle No. 3 (“Gorny”), aged 11 years (hatched in 2014), had been received from the public in 2016 after previously being used by a private individual for hunting. The bird first presented to the veterinary clinic in spring 2022 with chronic pododermatitis of 2–3 years’ duration (Fig 1). Medical history revealed that all three eagles were kept in the same aviary. The aviary was an open-type cage with one solid wall and partial rain cover. The remaining walls and 50% of the ceiling were made of wire mesh. The aviary had perches made of thick branches 7–10 cm in diameter.

The birds were fed raw red meat purchased from the local market and were given fresh water *ad libitum*; feeders and drinkers were placed directly on the floor of the aviary. At the time of inspection the bedding was visibly saturated (water seeping out under pressure) after recent rain and snowfall. The eagles remained aggressive and anxious during handling, which is typical of this species. On clinical examination, all three birds showed wet plumage with damp, ruffled and damaged feather structure. The birds were generally depressed, with reduced appetite, weak responses to external stimuli and soft vocalizations. The two younger eagles were still able to reach their perch in short flights, whereas the older eagle attempted unsuccessful escape flights when approached and retreated into a corner of the aviary, limping when ambulating and frequently sitting down. On closer examination, the older bird also exhibited a sticky, soiled feather coat and reduced peripheral skin temperature, consistent with poor general condition.

According to veterinary specialists, the affected areas on the soles of the birds’ feet were treated with Levomekol ointment (chloramphenicol) and then bandaged. The treatment had no effect, the positive dynamics were short-lived, the affected area increased and bled regularly. After rain or the formation of puddles in the enclosure, the suppuration process on the soles of the eagles’ feet intensified, and the condition of the wild birds deteriorated.

A visual examination revealed that the gauze bandage was wet, dirty and saturated with liquid from the litter. Under the bandage, inflammation of the horny coverings of the soles (the horny corium of the foot) was detected. The skin was thin and, when attempts were made to remove the bandage or take a sample of biomaterial, it cracked easily and bled for a long time.

### Sample collection

Sampling was performed as follows: prior to sampling, the footpads of wild birds were rinsed with water to remove dirt and litter particles, then treated with 70% alcohol, carefully wiping the affected pad with a swab [21]. The biological material consisted of the following: epidermal scales, scrapings, and pieces of epithelial tissue.

Sampling of biological material was performed from the affected area of the bird’s foot pads using skin scrapings, by excising necrotic tissues, scraping scabs from the affected areas with a scalpel until the first drops of blood appeared, removing tissue fragments from the depth of the affected wounds with forceps on day 0, and plucking 1–2 sticky, ruffled feathers from the body [22]. Bedding samples were taken from the location of the birds, near the feeder.

Immediately after collection, the biomaterial samples were placed on prepared dry CompactDry YM plates (Nissui Pharmaceutical, Japan) and incubated at 28 °C for 1–3 days [23].

The isolation and identification of pure cultures, as well as the analysis of cultural and biochemical properties, were carried out in the microbiology laboratory of the Engineering Center for Organic Agricultural Technologies at the S. Seifullin Kazakh Agricultural Technical Research University. Yeast isolates were recovered by sub-culture on Sabouraud dextrose agar (HiMedia, M063) and incubated at 28 °C for up to 10 days until typical colonies developed [24,25]. To assess thermotolerance, the isolates were sub-cultured in parallel at 8 °C, 28 °C and 37 °C [26].

Microscopic analysis of the morphological structures of micromycetes was performed using an AxioScope A1 trinocular transmitted light microscope (Zeiss, Germany) at ×40 magnification, and microstructures were studied using an Olympus microscope [26,27].

To study saccharolytic properties, Giss culture media containing glucose, mannitol, lactose, sucrose, and maltose were used. Cultures were inoculated into test tubes using the puncture method and incubated at 28°C under constant observation for changes in color, turbidity, and gas production [28]. Biochemical characterization was performed by conventional in-house assays on individual classical media (Giss media, Christensen urea agar, meat-peptone gelatin, skim milk, peptone water, blood and keratin-enriched Sabouraud agar) rather than commercial identification panels such as API 20C AUX or ID 32 C (bioMérieux), since these systems were not available in our laboratory. Definitive species-level identification therefore relied on ITS rDNA sequencing, while the biochemical assays were used to characterize the metabolic and pathogenicity-related phenotypes of the isolates.

To detect urease activity, the ability of fungi to break down urea into ammonia was determined on Christensen medium (HiMedia, M112) supplemented with 40% urea. Changes in the medium occurring during cultivation were assessed visually, and the intensity of the reaction was expressed in crosses (from “+” to “++++”) [26,28].

The proteolytic activity of the yeast was studied on gelatin, peptone, and skim milk. To assess the yeast’s ability to hydrolyze gelatin, meat-peptone agar supplemented with gelatin was used [29].

To study casein hydrolysis, cow’s milk was used, which had been previously skimmed by centrifugation at 3000 rpm and 2°C for 60 minutes. Inoculation was performed by the streak method in test tubes, followed by incubation in a thermostat at 28°C for 7 days [28,30].

The ability of yeast to utilize peptone was assessed using peptone water. Yeast cultures were inoculated into sterile test tubes containing peptone water, then incubated in a thermostat at 28 °C for 5 days. The assessment was based on growth criteria: positive peptone assimilation was indicated by clouding of the medium and the formation of a precipitate. In the case of a negative result, the medium remains clear [30].

Saccharolytic activity was studied on Giss media, urease activity on Christensen media with 40% urea and proteolytic activity on media enriched with peptone, gelatin, skim milk and sheep blood [31].

Keratinolytic activity was assessed using a modified Sabouraud medium enriched with 2% keratin [24,31,32].

The keratinolytic properties of micromycetes were detected using an in vitro hair perforation test described by Ajello L. (1967). For this, short hair or wool strands were placed in Petri dishes, autoclaved for 10 minutes at 120 °C and 25 mL of sterile distilled water and 2–3 drops of 10% sterile yeast extract were added. These dishes were inoculated with a yeast culture grown on Sabouraud agar with glucose. The inoculated Petri dishes were incubated at 25 °C for 20–30 days with regular microscopic monitoring. Typically, the hair becomes covered with mycelium, beneath which perforations can be observed under a microscope [24,31]. For each isolate, at least 30 hair fragments were examined microscopically at ×400 magnification on days 7, 14, 21 and 30 of incubation. Keratinolytic activity was scored semi-quantitatively, based on the proportion of hair fragments showing wedge- or cone-shaped perforations of the cortex: negative (no perforations on any of the examined fragments), low (perforations on ≤25% of fragments, single perforations per hair), moderate (perforations on 26–50% of fragments) and high (perforations on >50% of fragments, with multiple perforation sites per hair). All assays were performed in duplicate; isolates were classified as “high” only when both replicates met this criterion.

The susceptibility of *C. metapsilosis* isolates to antifungal agents was determined using the disk diffusion method in accordance with CLSI M44-A2 recommendations for yeasts. Susceptibility was assessed based on the diameters of growth inhibition zones according to CLSI interpretation criteria [33]. The disk diffusion assay was performed on Mueller-Hinton agar supplemented with 2% glucose and 0.5 µg/mL methylene blue, inoculated with a yeast suspension adjusted to a 0.5 McFarland standard, and incubated at 35 ± 2 °C for 24 h before reading inhibition zone diameters. We note that CLSI M44 currently provides validated zone-diameter breakpoints only for fluconazole and voriconazole against a limited number of *Candida* species, and that no species-specific clinical breakpoints are available for *C. metapsilosis* or for nystatin, clotrimazole, ketoconazole and amphotericin B. Inhibition zones were therefore interpreted using categorical thresholds previously applied to disk-diffusion testing of yeasts in veterinary studies: ≥ 20 mm – susceptible (S), 15–19 mm - intermediate/dose-dependent susceptibility (I/SDD), < 15 mm – resistant (R). The results obtained should therefore be considered a screening characterization of the isolates rather than formal clinical susceptibility categorization; broth microdilution according to CLSI M27 (reference standard for yeasts) will be performed in a follow-up study to determine MIC values.

For molecular identification of the species, a small amount of cell biomass was selected from each culture and homogenized. The homogenate was placed in an extraction buffer (0.1 M Tris (pH 8), 0.01 M EDTA, 2% SDS) with the addition of Proteinase K (20 ng/μL), 10% TAV, and 5 M NaCl. Next the extraction was performed with a mixture of chloroform and isoamyl alcohol (24:1). Then, the DNA was precipitated with ethanol, purified, dissolved in 1 × TE buffer and stored at −20°C for subsequent analysis. The amount and purity of the isolated DNA were determined by measuring the optical density at 260 and 280 nm using a NanoDrop 2000 device (Thermo Scientific, USA) [34,35].

PCR amplification of the variable ITS region of rDNA was performed using universal primers ITS1 (5′TCCGTAGGTGAACCTGCGG3′) and ITS4 (5′TCCTCCGCTTATTGATATGC3′) [36,37]. Amplification was performed using HS-Taq PCR Biomasters (2×) (Biolabmix LLC, Novosibirsk, Russia), with 10 pmol of each primer and 100 ng/μl of fungal DNA as a template. The reaction was performed with an initial denaturation at 95°C for 5 min, followed by 35 cycles consisting of: denaturation at 95°C for 30 sec, annealing at 57°C for 30 sec and elongation at 72°C for 60 sec. Final extension at 72°C for 7 min. The results of the PCR analysis were interpreted by gel electrophoresis and documented using a GelDoc XR+ transilluminator (Bio-Rad, Hercules, CA, USA). Sequencing was performed on a SeqStudio genetic analyzer (Thermo Fisher Scientific, USA) [36]. Each ITS amplicon was sequenced in both directions using the ITS1 and ITS4 primers, and consensus sequences covering the complete ITS1-5.8S-ITS2 region were assembled from the bidirectional reads. The initial sequencing run produced incomplete reads for two isolates; therefore, the PCR and bidirectional sequencing were repeated, yielding consensus sequences of the expected length for fungal ITS identification [38,39].

The obtained nucleotide sequences were manually checked and edited using BioEdit software (version 7.0) before analysis using BLAST against reference sequences in GenBank (https://www.ncbi.nlm.nih.gov/).

Ethics statement. The study protocol, including sampling of biological material from captive steppe eagles (*Aquila nipalensis*) presenting with clinical pododermatitis, was reviewed and approved by the Local Ethics Committee on Biological and Medical Ethics for Animal Research of the S. Seifullin Kazakh Agrotechnical Research University (Protocol No. 2, dated 1 November 2023). All sampling and treatment procedures were performed by qualified veterinary specialists on animals under regular clinical care, and biological material was obtained as part of routine diagnostic work-up of the lesions; no procedures were performed solely for the purpose of this study. Animals were handled in accordance with the International Guiding Principles for Biomedical Research Involving Animals (CIOMS, 1985) and with applicable national regulations of the Republic of Kazakhstan on the humane treatment of animals. Owners of the birds (the holding facility) provided written informed consent for the use of clinical data and biological material from their animals for research and publication.

## Results

**Fig 2:**
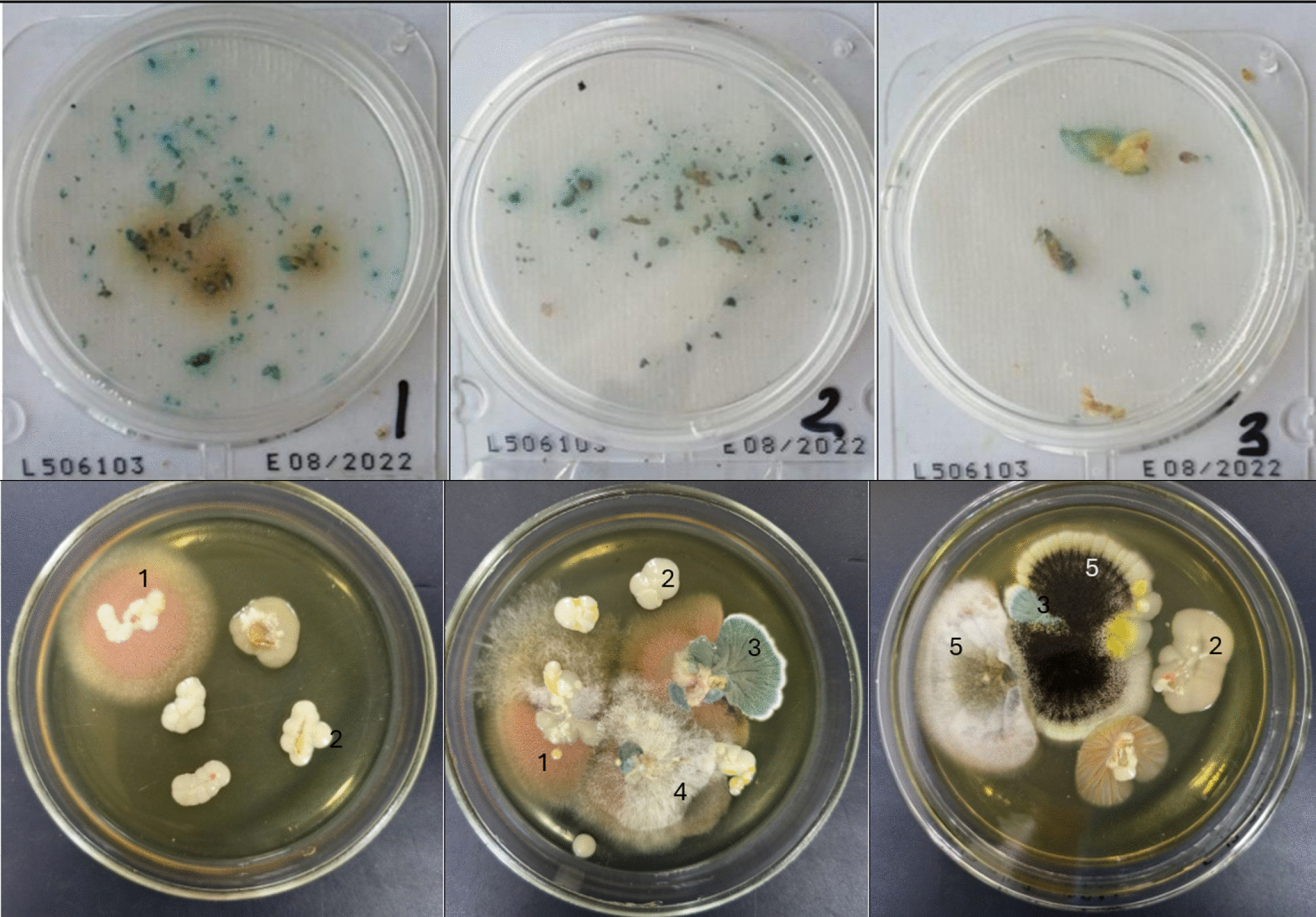
Growth of microfungal colonies: on CompactDry YM media (top row), on Sabouraud medium (bottom row). 1 – *Fusarium* spp., 2 – *Candida* spp., 3 – *Penicillium* spp., 4 – *Mucor* spp., 5 – *Aspergillus* spp.

**Fig 3:**
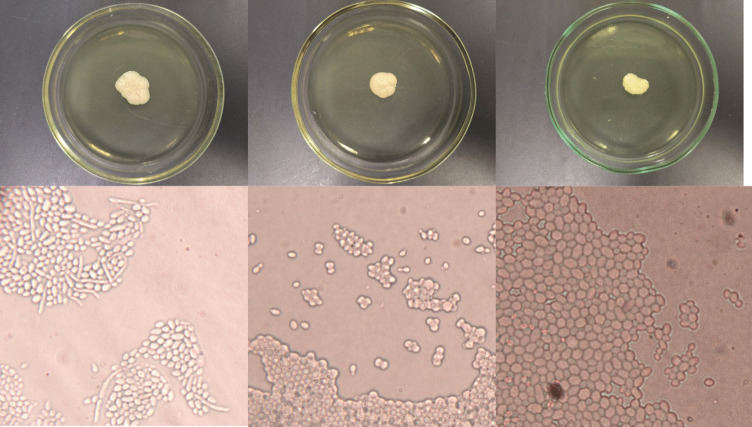
Appearance of colonies on media and yeast isolate cells.

By the end of the first day, multiple blue colonies (yeast growth) and rare brown-green colonies (mold growth) were observed on the CompactDry YM medium (Fig 2, top row). Various molds and yeasts were observed growing on Sabouraud media (Fig 2, bottom row). Analysis of the primary culture revealed the growth of yeast characteristics of *Candida* spp. The cultural and morphological properties of yeast strains isolated from wild eagles and litter are shown in Fig 3. Molecular genetic identification of isolates allowed strains to be identified as *Candida metapsilosis* and nucleotide sequences of isolates to be deposited in the GenBank database of the NCBI (USA).

**Fig 4:**
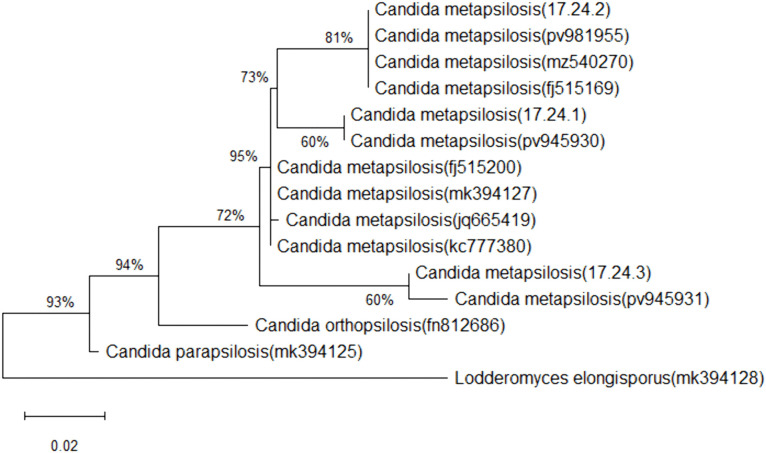
Maximum-likelihood phylogenetic tree of the ITS1–5.8S–ITS2 rDNA region showing the position of the three steppe-eagle *Candida metapsilosis* isolates among reference *Candida* sequences from GenBank.

The phylogeny was inferred using the Maximum Likelihood method and Tamura-Nei (1993) model [40] of nucleotide substitutions and the tree with the highest log likelihood (−1,398.60) is shown. The initial tree for the heuristic search was selected by choosing the tree with the superior log-likelihood between a Neighbor-Joining (NJ) tree [41] and a Maximum Parsimony (MP) tree. The NJ tree was generated using a matrix of pairwise distances computed using the Tamura-Nei (1993) model [40]. The MP tree had the shortest length among 10 MP tree searches; each performed with a randomly generated starting tree. The analytical procedure encompassed 12 nucleotide sequences with 309 positions in the final dataset. Evolutionary analyses were conducted in MEGA12 [42] (Fig 4).

**Fig 5:**
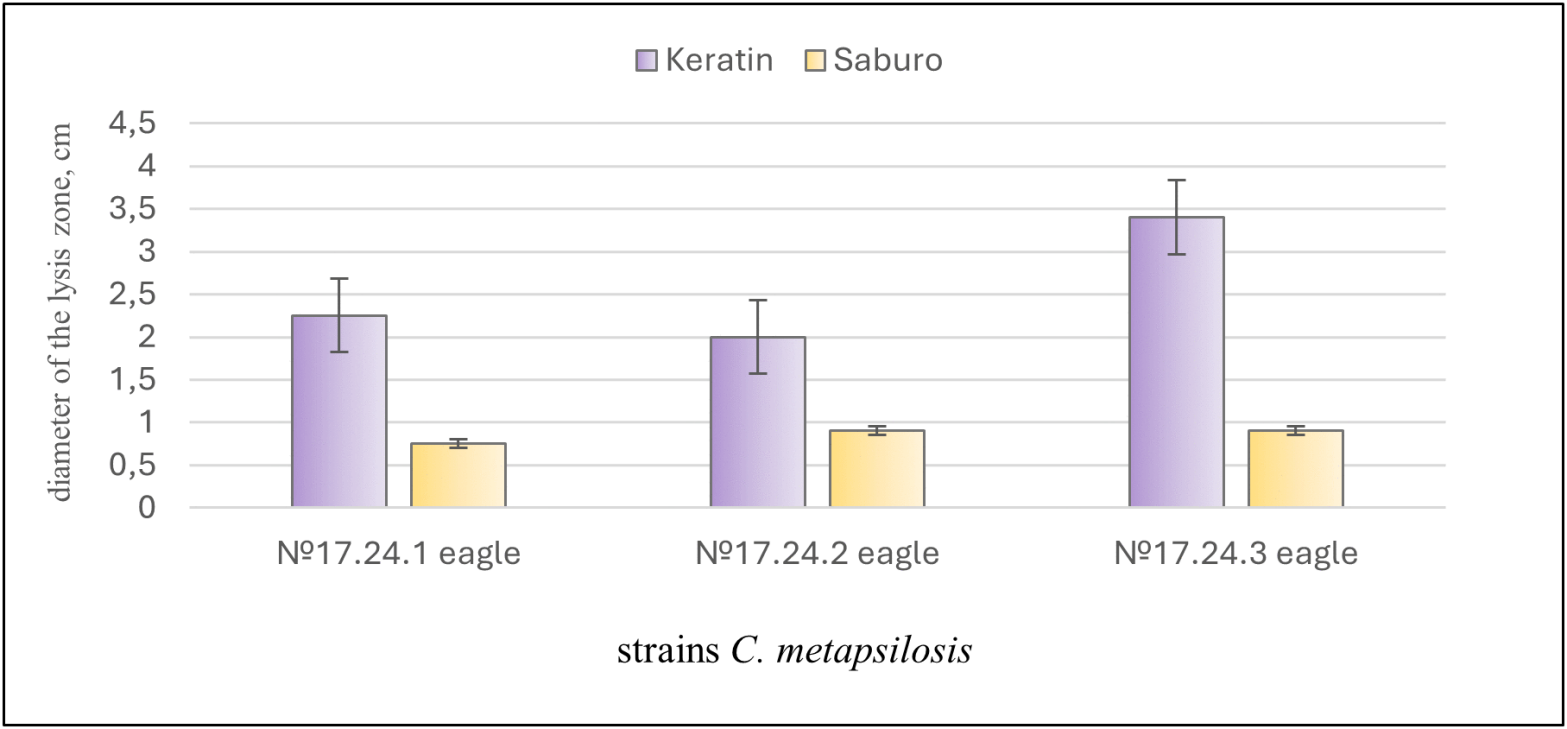
Keratinophilic activity of *C. metapsilosis* strains.

Biochemical analysis showed that *C. metapsilosis* yeast strains have high saccharolytic activity, breaking down all carbohydrates in the sequence maltose>sucrose>lactose>mannitol>glucose, weak proteolytic activity, selectively breaking down hemoglobin, gelatin, and peptone (Table 1). High keratinophilic (Fig 5) and urease (Table 1) activity was noted in all three strains.

**Table 1:** Biochemical activity of *Candida metapsilosis* strains isolated from wild eagles.

Substrate	№17.24.1 eagle	№17.24.2 eagle	№17.24.3 eagle
**Sucrose**	**4**	**2**	**2**
**Lactose**	**2**	**3**	**3**
**Glucose**	**2**	**1**	**1**
**Maltose**	**4**	**3**	**4**
**Mannitol**	**1**	**2**	**3**
**Hemoglobin**	**0**	**0,75**	**0,8**
**Casein**	**0**	**0**	**0**
**Gelatin**	**0**	**0**	**1**
**Peptone**	**0**	**1**	**0**
**Urea**	**4**	**4**	**3**

**Fig 6:**
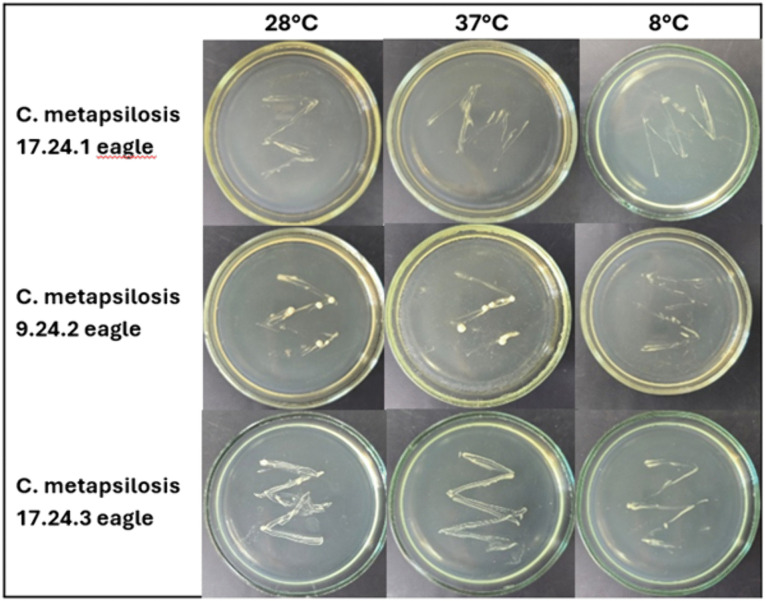
Growth of C. metapsilosis under various temperature conditions: 1 – Strain *C. metapsilosis* 17.24.1 eagle, 2 – Strain *C. metapsilosis* 9.24.2 eagle, 3 – Strain *C. metapsilosis* 17.24.3 eagle.

The analysis of the thermal tolerance of the strains revealed that all three strains retain the ability to grow at +8 ° C and at +37 ° C and the growth activity and rate of biomass accumulation in two strains was more pronounced at +37 ° C (Fig 6).

### Sensitivity of strains to antifungal drugs

**Fig 7:**
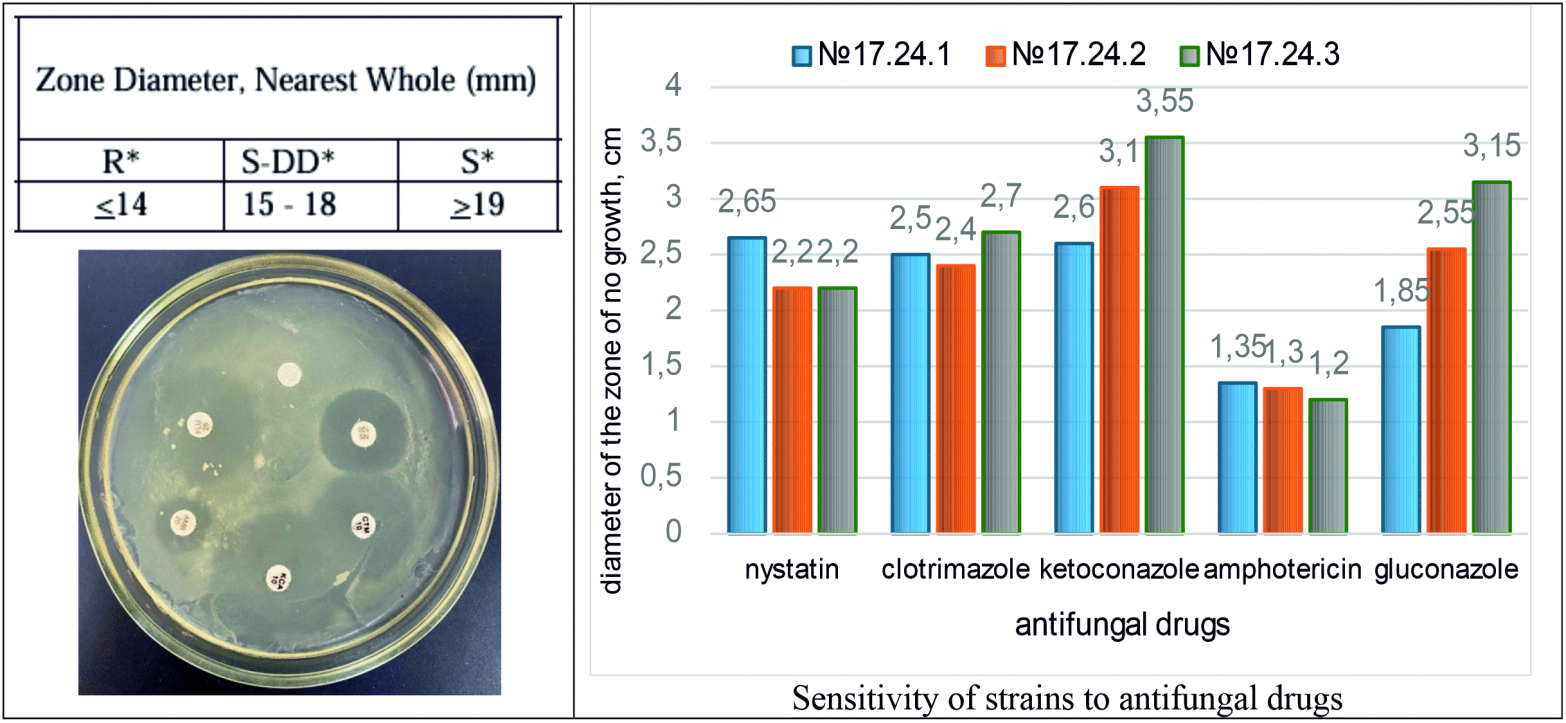
Susceptibility of *C. metapsilosis* strains to antifungal agents.

Sensitivity testing of the strains to antifungal agents, performed using the disk diffusion method in accordance with CLSI M44-A2 guidelines for yeasts, showed that the yeasts are susceptible to a number of drugs. All three strains are highly sensitive to ketoconazole, two are sensitive to clotrimazole and fluconazole and one is sensitive to nystatin. All strains are weakly sensitive to amphotericin (Fig 7).

**Fig 8:**
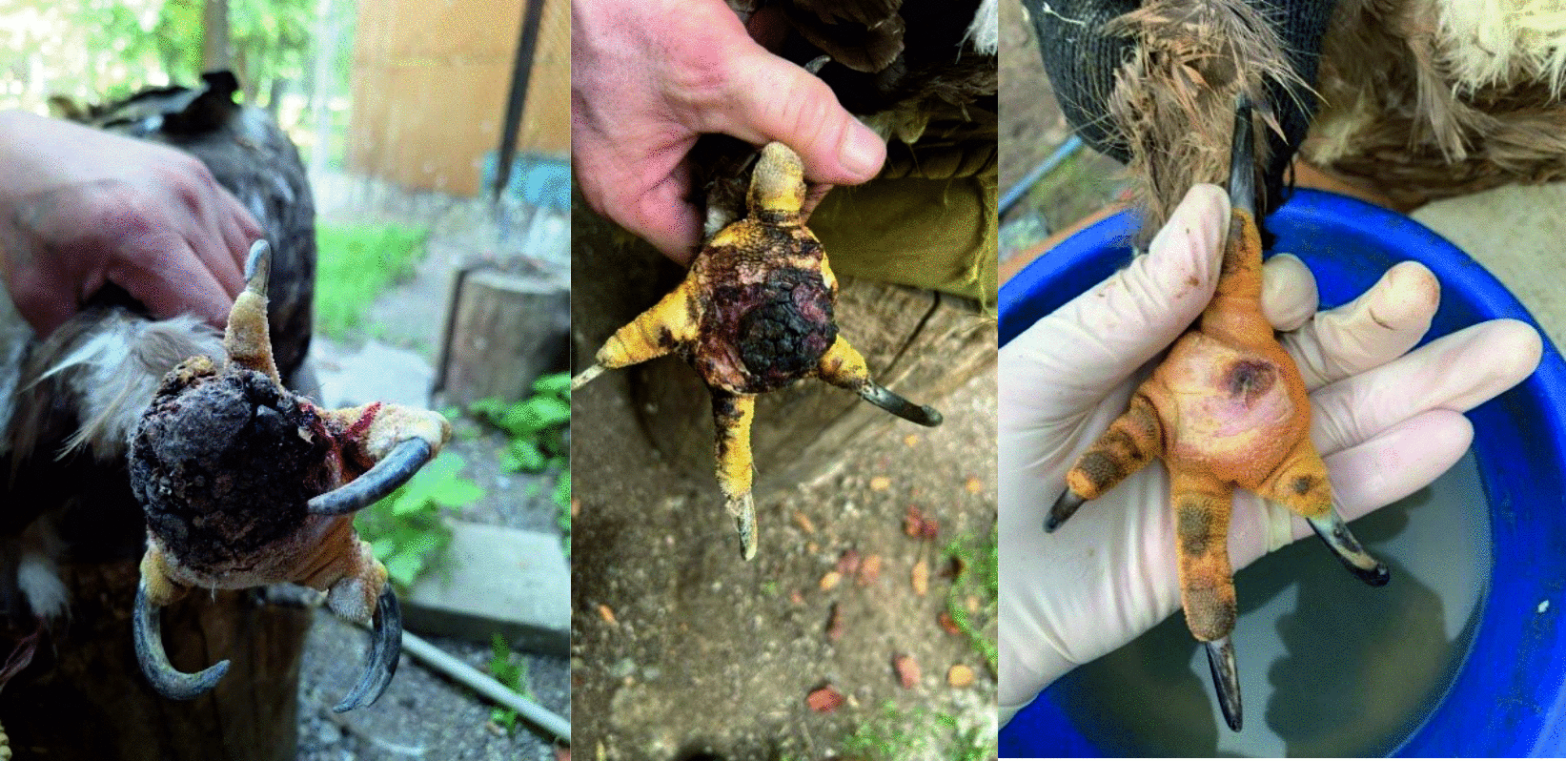
Stages of wound healing on the paw pads of steppe eagles.

Treatment with Clotrimazole 1% ointment (Sintez, PAO, Russia) for external use once a day was prescribed, alternating with Fluconazole ointment (Werteks, Russia) until a positive effect was achieved. After two weeks, Humic-Salve liniment based on potassium humate (BioNanoPreparat, Kazakhstan) was applied topically to the paw pads to accelerate the epithelialization of the wound surface. To increase the natural resistance of the bird’s body, the drinking feed supplement Gumka-KZ based on potassium humate (Kaztechnougol Scientific and Production Association LLP & BioNanoPreparat, Kazakhstan) with drinking water at a dose of 0.15 ml per 1 kg for a month. The treatment led to recovery, with positive dynamics in the healing of lesions (Fig 8).

The effectiveness of treatment was monitored by mycological examination of biomaterial samples taken from birds. Clinical recovery of eagles occurred on day 15, and mycological recovery (negative test results) on day 21. Relapses were observed after rain in an adult eagle nicknamed “Stary” which were successfully treated with liniment after 3–5 days.

To prevent yeast pododermatitis in wild eagles recommended to replace bedding in a timely manner, especially after rain; treating perches and floors with universal disinfectants or antifungal drugs; giving eagles the Gumka-KZ feed supplement for 10 days a month for six months to boost their immunity.

## Discussion

The genus *Candida* includes fungal species of yeast-like organisms. Of the approximately 200 species belonging to the genus, only 20 species are most isolated from clinical samples [43,44]. Although *C. albicans* is considered the main causative agent of candidiasis in human and veterinary medicine, other *Candida* species are currently most commonly detected [45]. Various authors have mentioned the isolation of pathogens such as *Candida parapsilosis*, *Candida orthopsilosis, Candida metapsilosis, Candida krusei*, *Candida lusitaniae, Candida glabrata* and *Candida tropicalis* from humans [43,46–50]. In this study, we isolated and characterized three strains of yeast-like fungi that caused chronic yeast pododermatitis in wild eagles from the Kazakh population that had been living in captivity for a long time. The strains were identified as *C. metapsilosis* by DNA sequence analysis and deposited in GenBank under numbers PV945930, PV981955 and PV945931. A BLAST search for *Candida metapsilosis* sequences revealed previously deposited yeast sequences from animal sources: in Italy from cattle (PV416737), in Brazil from the common nosuha (MF797776), and in Peru from the red paco fish (MZ540270). There were more *C. metapsilosis* strains identified from humans with lesions of the skin or its appendages: from skin in Malaysia (MH715400); from a skin granuloma in China (PQ626156); from nails in the Netherlands (MK561031) and in Egypt (KU200445); from patients diagnosed with onychomycosis in Shanghai, China (KJ816926, KJ816917, KJ816927). The largest number of *C. metapsilosis* strains (53 deposited sequences) were recovered from patients with internal organ involvement, disseminated candidiasis, candidemia and related conditions.

A comparative analysis of nucleotide sequences with the closest homologs in GenBank revealed the highest homology between the *C. metapsilosis* nucleotide sequences isolated from wild eagles of the Kazakh population and the culture derived from the type strain *Candida metapsilosis* PV416737, isolated from cattle in Italy, which was used as the reference sequence. All three isolates clustered closely with this reference *C. metapsilosis*. The sequence of strain *C. metapsilosis* 17.24.1 differs minimally from the reference sequence, while strain *C. metapsilosis* 17.24.2 is closest to the reference sequence, while *C. metapsilosis* 17.24.3 contains a larger number of single-nucleotide substitutions and may represent the most divergent of the three isolates.

Noteworthy is the maximum homology with the nucleotide sequences of *C. metapsilosis* strains isolated from the intestinal microbiome of fish (MZ540270), marine sediments in India (JQ665419), wastewater in Brazil (QR731586) and the sea surface and underlying waters in Taiwan (FJ515169, FJ515199, FJ515200). This may indicate the high resistance of *C. metapsilosis* strains in the litter of the room where the eagles lived and prove that the birds were reinfected over a long period of time. *C. metapsilosis* strains inhabiting the litter are likely to be highly adaptable and resistant, which may contribute to prolonged reinfection of birds in aviary conditions. This may be related to thermotolerance and the ability to form biofilms, which allows yeast cells to remain viable for long periods [51].

The first reported case of *C. metapsilosis* isolation from wild eagles of the Kazakh population kept in long-term captivity is consistent with the clinical picture observed in this case: the attending veterinarians initially treated the lesions as bacterial pododermatitis and did not prescribe antifungal therapy, which may have contributed to the chronic course of the disease. Because previous reports of pododermatitis in birds of prey have focused on bacterial agents, fungal involvement is rarely considered at the first diagnostic step, and we therefore suggest, rather than conclude, that broader use of mycological work-up in non-resolving cases could be useful.

A possible cause of the wild eagles’ infection with *C. metapsilosis* at the breeding facility was identified as a chronic infection of the foot pads in an older steppe eagle nicknamed “Stary” which had been kept in captivity for a long time under inadequate conditions by previous owners. A review of the medical history of the steppe eagle nicknamed “Stary” revealed that the bird had been kept in conditions with improperly constructed perches. Branches from the top of a felled tree, which were narrow in diameter, were used for this purpose. It is likely that the resulting increase in pressure on the small supporting surface of the feet led to microcracks and the entry of the pathogen. The disease was exacerbated by improper treatment without the use of antifungal medications, active exploitation, and a decrease in the bird’s overall resistance.

The clinical presentation in the three eagles was characteristic of chronic pododermatitis, with a prolonged disease course and intermittent discharge of purulent material from the footpad lesions, which led the attending veterinarians to a presumptive diagnosis of bacterial pododermatitis. This was also mentioned by other authors, who reported that wild birds in captivity often suffer from prolonged traumatic pododermatitis associated with improper husbandry and that the main causative agent of the disease is most often *S. aureus*. There are publications describing *E. coli*, *Pasteurella* spp., *Klebsiella* spp., *Clostridium* spp., *Corynebacterium* spp., *Bacillus* spp., *Diplococcus* spp., *Nocardia* spp., *Actinobacillus* spp., *Actinomyces* spp., *Aeromonas* spp., *Proteus* spp. and *Pseudomonas* spp. as causative agents of bacterial pododermatitis in wild and domestic birds [12].

However, prolonged treatment of bacterial pododermatitis and the use of antibiotics led to a decline in the birds’ immunity, which had already been weakened by poor housing conditions and intensive farming practices. It is known that damp litter, poor nutrition, and stress are classic factors predisposing to opportunistic infections [5,13–15]. Given that the fungus *C. metapsilosis* is an opportunistic pathogen that exploits vulnerabilities in the host and the environment, it was the cause of the yeast mycosis, which developed as a secondary infection when favorable conditions arose.

This was likely the cause of a secondary fungal infection of the footpads in wild eagles, caused by fungi of the genus *Candida*. The isolation of *Candida* spp. from affected sites in birds has been reported on multiple occasions [18]. Cases of *Candida* spp. infection are known in canaries (*Serinus canaria*), macaws (*Ara macao*) and cockatoos (*Nymphicus hollandicus*), pigeons (*Columba livia domestica*), geese (Anser/Branta sp.), guinea fowl (*Numida meleagris*), pheasants (*Phasianus colchicus*), quails (*Coturnix coturnix*), parrots (*Psittacus* sp.), and other birds [14,15,19,43–46].

Reports of fungal infections in captive birds mention the role of *Candida albicans* in skin lesions in hatched hornbill chicks and adult lapwings and curlews [11]. *C. albicans* more often cause cutaneous candidiasis or fungal skin infection which can often occur in connection with systemic infections or weakened immunity. The cutaneous form of the infection can also spread among birds in the presence of infected mites and the use of dirty feeding utensils [5,47]. There have been reports of candidiasis developing because of poor housing conditions and uncleanliness [14,15,48]. There is a known case of systemic candidiasis caused by *C. parapsilosis* in a 20-year-old blue-fronted Amazon parrot with lameness [19]. To our knowledge, no previous reports describe the role of *Candida metapsilosis* in lesions of the footpads of wild eagles living in the wild or in captivity. Our research has expanded the list of pathogens of wild eagles living in captivity in Kazakhstan and proposed a treatment regimen.

The pathogen causing chronic yeast pododermatitis in wild eagles from the Kazakh population, which have been living in captivity for a long time, was identified based on morphological characteristics *in vitro*: the shape and color of colonies and reverse and the size and shape of yeast cells. Attention was paid to the speed and nature of growth tube formation on agarized media, thermotolerance, the ability to assimilate urea, proteins and carbohydrates and to decompose keratin. Joshi KR and co-authors pointed out the need to take these characteristics into account when differentiating pathogenic yeasts from non-pathogenic yeast pathogens [49].

Mycological diagnosis allowed the pathogens to be classified as belonging to the genus *Candida*. The pure culture of all three strains of *C. metapsilosis* was distinguished by characteristic features. On Sabouraud agar, the colonies grew rapidly, forming uniformly rounded, smooth, creamy, yellowish colonies with a diameter of 1.0–2.0 mm, which became matte with age and formed zones separated by deep furrows, growing unevenly to 1.0 × 1.5–1.5 × 1.8 cm in diameter. It should be noted that all strains of *C. metapsilosis* were completely undemanding in terms of nutrient medium, grew well and formed characteristic colonies on Sabouraud dextrose agar at a temperature of – 28 ± 1 °C. During primary isolation, soft velvety zones of young colonies with pink-red pigment formed around the central typical creamy light cream colony.

Vegetative structures identified under a microscope in strain *C. metapsilosis* №17.24.1 – pseudomycelium (pseudohyphae), rudimentary pseudomycelium, actively budding cells; in strains *C. metapsilosis* №17.24.2 and *C. metapsilosis* №17.24.3, budding cells were identified, no mycelial structures were identified. The fungi had uniform yeast cells of round, oval and ellipsoidal shape, with clearly visible differences in smears (Fig 5). The budding cells of all strains had both single daughter cells and cells attached to the mother cell, being practically the same size. A similar morphology of *C. metapsilosis* yeast has been described by Gómez-Gaviria M. et al. and other authors [32].

The literature mentions a controversial issue regarding the ability of *C. metapsilosis* to form pseudohyphae. Several studies report on the formation of pseudohyphae by *C. metapsilosis* strains [50,51]. An analysis of 93 different isolates of *C. parapsilosis* sensu lato conducted by Pryszcz LP et al. (2015) revealed that *C. metapsilosis* does not possess such morphology [52]. It should be noted that the two strains we isolated from eagles were also unable to form pseudohyphae. In contrast, strain *C. metapsilosis* №17.24.1, which was obtained from an elderly steppe eagle that had been ill for many years, formed clearly visible pseudohyphae and rudimentary pseudomycelium. This may be related to strain-specific differences among the analyzed isolates; however, in the absence of whole-genome sequencing we cannot rigorously establish a genetic basis for this phenotype, and we therefore present this observation as a working hypothesis rather than a firm conclusion. Although all isolates were subcultured under nominally identical conditions (medium, temperature, pH, inoculum size, incubation time), we cannot fully exclude an effect of the in vitro micro-environment at the time of subculture, including subtle differences in cell density, oxygen tension and the age of the original primary culture, which have been shown to modulate pseudohyphal morphogenesis in other members of the *C. parapsilosis* complex [32]. Whole-genome sequencing and comparative transcriptomics of the three isolates are planned in our future studies to clarify whether the pseudohyphal phenotype of strain №17.24.1 reflects genuine genetic divergence or phenotypic plasticity.

All three strains were characterized by high keratinolytic and urease activity, indicating the potential pathogenicity of the pathogens [53–55]. The presence of saccharolytic and proteolytic properties indicates the high potential for nutritional adaptability of the isolates and their ability to break down various substrates in the external environment, which allows them to remain viable in the external environment for a long time and acquire virulent and pathogenic properties during microorganism-host interactions [56].

The sensitivity of *C. metapsilosis* strains to all antifungal drugs included in the study was determined. All three strains were highly sensitive to ketoconazole, two were sensitive to clotrimazole and fluconazole and one was sensitive to nystatin. At the same time, all strains were weakly sensitive to amphotericin B. The ambiguous sensitivity of *Candida* strains, including *C. metapsilosis*, to various antimycotics has been reported previously [56,57]. Considering the classic treatment regimens for candidiasis, the availability of drugs in city pharmacies and sensitivity, clotrimazole and fluconazole were recommended for treatment, which had also been used previously for such purposes [47,58].

It should be noted that several other filamentous fungi (*Fusarium* spp., *Penicillium* spp., *Mucor* spp. and *Aspergillus* spp.) were also recovered from the primary cultures (Fig. 4). These are common environmental molds, and their isolation most likely reflects contamination of the wet, soiled bedding rather than an etiological role in the foot lesions: they were not recovered consistently from all three birds, did not dominate the subcultures from the deep tissue scrapings, and on subculture from the affected footpad tissue only *Candida*-like colonies were repeatedly and abundantly recovered, with similar morphology in all three eagles. Furthermore, only the *Candida* isolates combined the in vitro features most relevant to footpad pathology thermotolerance up to 37 °C, urease activity and clear keratinolytic activity in the hair-perforation assay. Together with the clinical response to topical antimycotic therapy, these observations support *C. metapsilosis* as the most likely fungal contributor to the chronic lesions, although we cannot formally exclude an additional, transient role of other environmental molds. The keratinolytic activity, thermotolerance and (in one strain) pseudohyphal formation of these isolates are consistent with an opportunistic pathogen that can play a role in the chronic progression of pododermatitis in immunocompromised birds kept under poor husbandry conditions, rather than with a primary, obligate pathogen. A major limitation of the present work, which we underline here in addition to the limitations listed below, is the absence of histopathology of the affected footpad tissue, which would have allowed direct visualization of fungal elements within the lesions and a more rigorous assessment of tissue invasion versus surface colonization; histopathological correlation should therefore be a priority in any future study of fungal pododermatitis in raptors.

In conclusion, we report that the correctly chosen tactics and strategy of therapeutic measures allowed us to completely cure steppe eagles from candidal pododermatitis. The prescription of antimycotics in combination with humic ointment and feed additives led to the recovery of steppe eagles, and the use of the correct perch prevented further reinfection of wild birds with *C. metapsilosis* by normalizing the natural resistance of the organism.

### Limitations

This study was limited by several objective factors, which the authors consider necessary to inform the potential reader.

- Small sample size: a series of observations of three birds from a single aviary.

- Absence of histological pathology.

- No control group: samples were not collected from healthy birds in the same or different habitats to determine whether *C. metapsilosis* is a normal part of the eagle’s skin microbiome or the environment.

- Concomitant factors: the birds had serious husbandry issues and had previously received antibiotic treatment, which are major risk factors for fungal overgrowth.

## Conclusion

*C. metapsilosis* yeast fungi were isolated from wild steppe eagles of the Kazakhstan population diagnosed with chronic pododermatitis that had been kept in captivity for a long time. *C. metapsilosis* yeast from the lesions was characterized by using cultural-morphological, biochemical, and molecular-genetic methods. Cultural and morphological characterization confirmed their belonging to the genus *Candida*, while molecular genetic identification of the pathogen strains revealed their identity with *C. metapsilosis* sequences. *C. metapsilosis* isolates exhibited high keratinolytic and urease activity, thermotolerance with growth at temperatures up to 37 °C, marked saccharolytic activity (with preferential utilization of maltose, sucrose and lactose over mannitol and glucose), and weak, selective proteolytic activity towards hemoglobin, gelatin and peptone. Disk diffusion screening showed that all three isolates were susceptible to ketoconazole, two were susceptible to clotrimazole and fluconazole, and one was susceptible to nystatin, while all three showed reduced susceptibility to amphotericin B. Targeted topical antifungal therapy combined with improved husbandry led to clinical and mycological resolution of the lesions. Taken together, our findings support a role for *C. metapsilosis* as an opportunistic fungal contributor to chronic pododermatitis in captive raptors and emphasize the need for mycological work-up of non-resolving footpad lesions in birds of prey.
