# Correction: Isolation and characterization of *Candida metapsilosis* from foci of chronic pododermatitis in captive steppe eagles in Kazakhstan

**DOI:** 10.1371/journal.pone.0360045

**Published:** 2026-10-06

**Authors:** Elena Kukhar, Gulshat Bailina, Aziza Nessipbayeva, Rano Sattarova, Marat Turkeyev

There is an error in affiliation 1 for authors Elena Kukhar, Gulshat Bailina and Aziza Nessipbayeva. The correct affiliation 1 is: Saken Seifullin Kazakh Agrotechnical University, Astana, Kazakhstan.
