# Supplementary material for: Isolation and characterization of Candida metapsilosis from foci of chronic pododermatitis in captive steppe eagles in Kazakhstan
Source: PLoS One. 2026 Jul 31;21(7):e0353552. doi: 10.1371/journal.pone.0353552 (PMC13426984; doi:10.1371/journal.pone.0353552)

PLOS One

The etiological role of the yeast-like fungus Candida metapsilosis in the development of chronic pododermatitis in captive steppe eagles

--Manuscript Draft--

**Manuscript Number:**	PONE-D-26-07402
**Article Type:**	Research Article
**Full Title:**	The etiological role of the yeast-like fungus Candida metapsilosis in the development of chronic pododermatitis in captive steppe eagles
**Short Title:**	Candida metapsilosis in the development of chronic pododermatitis in captive steppe eagles
**Corresponding Author:**	Aziza Nessipbayeva<br>Kazahskij agrotehničeskij universitet imeni Sakena Sejfullina: S Seifullin Kazakh Agro Technical Research University<br>Astana, KAZAKHSTAN
**Keywords:**	Candida albicans, opportunistic yeast, pododermatitis, wild birds, steppe eagle
**Abstract:**	Hunting with wild birds, especially golden eagles, is a Kazakh national tradition, a national idea, a sport and a way of life for hunters. Pododermatitis is a common disease of the sole of the foot in captive birds of prey, which is a serious problem worldwide. Keratinophilic yeasts are new pathogens of pododermatitis in wild birds, which, by destroying and metabolizing keratin in surface tissues, prevent wound healing and contribute to the chronization of the process. This, combined with their thermal tolerance and unpretentiousness to the substrate, enhances their pathogenicity.<br>In this study, we report on the identification of isolates of a new causative agent of yeast pododermatitis Candida metapsilosis from wild eagles of the Kazakh population living in captivity for a long time, the study of their biological properties and pathogenicity. Our results showed that isolates of Candida metapsilosis caused pronounced clinically similar skin lesions on the pads of the paws of wild eagles and showed cultural and morphological similarities with the genus Candida. Genetic identification using ribosomal gene sequencing revealed 98.9% identity with Candida metapsilosis sequences in three cases. C. metapsilosis isolates showed high keratinophilic and urease activity, high sensitivity to ketoconazole, clotrimazole, and fluconazole, and weak sensitivity to nystatin and amphotericin B.<br>Our results indicate the emergence of new pathogenic yeast strains of the genus Candida responsible for pathology in wild eagles in Kazakhstan. Combined treatment with antifungal drugs, humic liniment and a feed additive with potassium humate, improved conditions of detention led to complete wound healing. We recommend that after detection of pododermatitis in wild birds, including eagles, golden eagles, falcons and other birds involved in hunting or photo shoots, prolonged chronic course and difficult treatment, veterinary specialists conduct a comprehensive retrospective analysis of newly identified pathogens, including a complete description of their pathogenicity.
**Order of Authors:**	E.V. Kukhar
	G.E. Bailina
	Aziza Nessipbayeva
	R.S. Sattarova
	M.K. Turkeev
**Additional Information:**	
**Question**	**Response**
**Financial Disclosure**<br><br>Enter a financial disclosure statement that	Yes

Powered by Editorial Manager® and ProduXion Manager® from Aries Systems Corporation

describes the sources of funding for the work included in this submission. Review the submission guidelines for detailed requirements. View published research articles from *PLOS ONE* for specific examples.<br><br>This statement is required for submission and **will appear in the published article** if the submission is accepted. Please make sure it is accurate.<br><br>**Funded studies**<br>Enter a statement with the following details:<br>• Initials of the authors who received each award<br>• Grant numbers awarded to each author<br>• The full name of each funder<br>• URL of each funder website<br>• Did the sponsors or funders play any role in the study design, data collection and analysis, decision to publish, or preparation of the manuscript?<br><br>Did you receive funding for this work?	
Please add funding details.<br>as follow-up to "**Financial Disclosure**<br><br>Enter a financial disclosure statement that describes the sources of funding for the work included in this submission. Review the submission guidelines for detailed requirements. View published research articles from *PLOS ONE* for specific examples.<br><br>This statement is required for submission and **will appear in the published article** if the submission is accepted. Please make sure it is accurate.	The study was funded by grant AP23489001 (2024-2026) from the Ministry of Education and Science of the Republic of Kazakhstan.

**Funded studies**<br>Enter a statement with the following details:<br>• Initials of the authors who received each award<br>• Grant numbers awarded to each author<br>• The full name of each funder<br>• URL of each funder website<br>• Did the sponsors or funders play any role in the study design, data collection and analysis, decision to publish, or preparation of the manuscript?<br><br>Did you receive funding for this work?"	
Please select the country of your main research funder (please select carefully as in some cases this is used in fee calculation).<br>as follow-up to "**Financial Disclosure**<br><br>Enter a financial disclosure statement that describes the sources of funding for the work included in this submission. Review the submission guidelines for detailed requirements. View published research articles from *PLOS ONE* for specific examples.<br><br>This statement is required for submission and **will appear in the published article** if the submission is accepted. Please make sure it is accurate.<br><br>**Funded studies**<br>Enter a statement with the following details:<br>• Initials of the authors who received each award<br>• Grant numbers awarded to each author<br>• The full name of each funder<br>• URL of each funder website<br>• Did the sponsors or funders play any role in the study design, data collection and analysis, decision to publish, or preparation of the manuscript?<br><br>Did you receive funding for this work?"	KAZAKHSTAN - KZ
**Competing Interests**	The authors have declared that no competing interests exist.

Use the instructions below to enter a competing interest statement for this submission. On behalf of all authors, disclose any competing interests that could be perceived to bias this work—acknowledging all financial support and any other relevant financial or non-financial competing interests.<br><br>This statement is required for submission and **will appear in the published article** if the submission is accepted. Please make sure it is accurate and that any funding sources listed in your Funding Information later in the submission form are also declared in your Financial Disclosure statement.<br><br>View published research articles from *PLOS ONE* for specific examples.<br><br>**NO authors have competing interests**<br><br>Enter: *The authors have declared that no competing interests exist.*<br><br>**Authors with competing interests**<br><br>Enter competing interest details beginning with this statement:<br><br>*I have read the journal's policy and the authors of this manuscript have the following competing interests: [insert competing interests here]*<br><br>* typeset	
**Ethics Statement**<br><br>Enter an ethics statement for this submission. This statement is required if the study involved:<br><br>• Human participants<br>• Human specimens or tissue<br>• Vertebrate animals or cephalopods<br>• Vertebrate embryos or tissues	All procedures involving animals were reviewed and approved by the Ethics Committee of the S. Seifullin Kazakh Agro Technical Research University (Astana, Kazakhstan). The study involved captive steppe eagles kept under routine husbandry conditions. Clinical examination and sample collection were performed as part of standard veterinary care. No experimental infection, invasive procedures, euthanasia, or animal sacrifice were conducted. All efforts were made to minimize animal stress and suffering.

Powered by Editorial Manager® and ProduXion Manager® from Aries Systems Corporation

• Field research<br><br>Write "N/A" if the submission does not require an ethics statement.<br><br>General guidance is provided below. Consult the submission guidelines for detailed instructions. **Make sure that all information entered here is included in the Methods section of the manuscript.**	

**Format for specific study types**<br><br>**Human Subject Research (involving human participants and/or tissue)**<br>• Give the name of the institutional review board or ethics committee that approved the study<br>• Include the approval number and/or a statement indicating approval of this research<br>• Indicate the form of consent obtained (written/oral) or the reason that consent was not obtained (e.g. the data were analyzed anonymously)<br><br>**Animal Research (involving vertebrate animals, embryos or tissues)**<br>• Provide the name of the Institutional Animal Care and Use Committee (IACUC) or other relevant ethics board that reviewed the study protocol, and indicate whether they approved this research or granted a formal waiver of ethical approval<br>• Include an approval number if one was obtained<br>• If the study involved *non-human primates*, add *additional details* about animal welfare and steps taken to ameliorate suffering<br>• If anesthesia, euthanasia, or any kind of animal sacrifice is part of the study, include briefly which substances and/or methods were applied<br><br>**Field Research**<br><br>Include the following details if this study involves the collection of plant, animal, or other materials from a natural setting:<br>• Field permit number<br>• Name of the institution or relevant body that granted permission	
**Data Availability**<br><br>Authors are required to make all data underlying the findings described fully available, without restriction, and from the time of publication. PLOS allows rare exceptions to address legal and ethical concerns. See the PLOS Data Policy and FAQ for detailed information.	Yes - all data are fully available without restriction

A Data Availability Statement describing where the data can be found is required at submission. Your answers to this question constitute the Data Availability Statement and **will be published in the article**, if accepted.<br><br>**Important:** Stating 'data available on request from the author' is not sufficient. If your data are only available upon request, select 'No' for the first question and explain your exceptional situation in the text box.<br><br>Do the authors confirm that all data underlying the findings described in their manuscript are fully available without restriction?	
**Describe where the data may be found in full sentences. If you are copying our sample text, replace any instances of XXX with the appropriate details.**<br><br>• If the data are **held or will be held in a public repository**, include URLs, accession numbers or DOIs. If this information will only be available after acceptance, indicate this by ticking the box below. For example: *All XXX files are available from the XXX database (accession number(s) XXX, XXX.).*<br>• If the data are all contained **within the manuscript and/or Supporting Information files**, enter the following: *All relevant data are within the manuscript and its Supporting Information files.*<br>• If neither of these applies but you are able to provide **details of access elsewhere**, with or without limitations, please do so. For example:<br><br>*Data cannot be shared publicly because of [XXX]. Data are available from the XXX Institutional Data Access / Ethics Committee (contact via XXX) for researchers who meet the criteria for access to confidential data.*<br><br>*The data underlying the results presented in the study are available from (include the name of the third party*	The data will be made publicly available upon acceptance of the manuscript.

and contact information or URL).<br>• This text is appropriate if the data are owned by a third party and authors do not have permission to share the data.<br><br>* typeset	
Additional data availability information:	

Click here to access/download;Manuscript;Article.docx

The etiological role of the yeast-like fungus *Candida metapsilosis* in the development of chronic pododermatitis in captive steppe eagles

Kukhar E.V.[1], Bailina G.E.[1], Nessipbayeva A.E. [1]*, Sattarova R.S.[2], Turkeev M.K.[2]

[1] S. Seifullin Kazakh Agro Technical Research University, Astana, Kazakhstan,
[2] «Kazakh Scientific Research Veterinary Institute» LLP, Almaty, Kazakhstan

* Corresponding author: Nessipbayeva A.E.
E-mail: nessipbayeva2510@mail.ru (N.A.E.)

Abstract

Hunting with wild birds, especially golden eagles, is a Kazakh national tradition, a national idea, a sport and a way of life for hunters. Pododermatitis is a common disease of the sole of the foot in captive birds of prey, which is a serious problem worldwide. Keratinophilic yeasts are new pathogens of pododermatitis in wild birds, which, by destroying and metabolizing keratin in surface tissues, prevent wound healing and contribute to the chronization of the process. This, combined with their thermal tolerance and unpretentiousness to the substrate, enhances their pathogenicity.

In this study, we report on the identification of isolates of a new causative agent of yeast pododermatitis *Candida metapsilosis* from wild eagles of the Kazakh population living in captivity for a long time, the study of their biological properties and pathogenicity. Our results showed that isolates of *Candida metapsilosis* caused pronounced clinically similar skin lesions on the pads of the paws of wild eagles and showed cultural and morphological similarities with the genus *Candida*. Genetic identification using ribosomal gene sequencing revealed 98.9% identity with *Candida metapsilosis* sequences in three cases. *C. metapsilosis* isolates showed high keratinophilic and urease activity, high sensitivity to ketoconazole, clotrimazole, and fluconazole, and weak sensitivity to nystatin and amphotericin B.

Our results indicate the emergence of new pathogenic yeast strains of the genus *Candida* responsible for pathology in wild eagles in Kazakhstan. Combined

treatment with antifungal drugs, humic liniment and a feed additive with potassium humate, improved conditions of detention led to complete wound healing. We recommend that after detection of pododermatitis in wild birds, including eagles, golden eagles, falcons and other birds involved in hunting or photo shoots, prolonged chronic course and difficult treatment, veterinary specialists conduct a comprehensive retrospective analysis of newly identified pathogens, including a complete description of their pathogenicity.

Keywords: *Candida albicans*, opportunistic yeast, pododermatitis, wild birds, steppe eagle

Introduction

Pododermatitis is a common disease of the sole of the foot in captive birds of prey, affecting the metatarsal pad area of the foot [1, 2, 3, 4, 5]. Pododermatitis has been described in falcons that developed the condition in captivity while being used for falconry in Abu Dhabi in the United Arab Emirates [6, 7, 8]. Pododermatitis has been reported in other birds, including owls, birds of prey, eagles, geese, owls, ospreys, penguins, and swans [9, 10, 11, 12, 13, 14, 15, 16].

Possible causes of pododermatitis include poor housing conditions (unsuitable perches), excessive weight and lack of physical activity [3, 17, 18, 19], poor hygiene and uncleanliness [5, 20, 21].

As a rule, footpad dermatitis (FPD) in captive birds occurs when the integrity of the skin is compromised and inflammatory processes develop on the

plantar surface of the foot [22]. A distinctive feature of the disease is the chronic process of "necrosis" of the epithelium of the plantar surface of the foot, followed by the penetration of pathogens and deep inflammation of the tissues, leading to their destruction and complete loss of foot function [4, 23, 24].

The main infectious agent of footpad dermatitis (FPD) is usually Staphylococcus aureus [25]. However, other bacterial pathogens of avian pododermatitis are known, such as *E. coli*, *Pasteurella* spp., *Klebsiella* spp. and several others [16].

The isolation of *Candida* spp. microfungi from affected areas has been reported [22]. Fungal diseases in birds caused by opportunistic yeast fungi of the genus *Candida*, especially *C. albicans* [26], pose a serious threat to domestic and wild birds, both captive and free ranging [27].

Literature sources report a case of systemic candidiasis caused by *Candida parapsilosis* (to which *C. metapsilosis* belongs) in a 20-year-old blue-fronted Amazon parrot. The main clinical symptom in this bird was lameness [28]. There is no information on the role of *C. metapsilosis* in the development of diseases in wild birds kept in captivity, including candidal pododermatitis, and the biological properties of the pathogens have not been described [29].

We have established the first case of candidal pododermatitis caused by the pathogen *C. metapsilosis* in three wild steppe eagles from the Kazakh population that had been kept in captivity for a long time. The pathogens were isolated from bedding and biomaterial obtained from the affected areas of the steppe eagles' paw

pads, the main biological properties of the pathogens were studied, and the birds were treated until complete recovery with the combined use of antifungal drugs, humic liniment, and humic feed additive.

Materials and methods

Medical history collection

The Mycology and Fungal Biotechnology Laboratory at the S. Seifullin Kazakh Agrotechnical Research University received biomaterial from wild eagles kept in captivity and samples of bedding from the enclosure where they were kept, with complaints of chronic pododermatitis that did not respond to treatment with antimicrobial drugs.

Steppe eagle №1, nicknamed “Stary” approximately 40 years old, had been used for commercial purposes for many years – for photo shoots at the year-round mountain resort of Chimbulak in Almaty. He was brought to the zoo by the public in 1994. He was taken to a veterinary clinic in the fall of 2023. The first clinical signs and behavioral disturbances were observed in October 2023. The initial diagnosis was pododermatitis, which he had been suffering from for more than three years.

Steppe eagle №2, nicknamed “Orlik,” born in 2018, arrived from the public in 2019, has been suffering from pododermatitis for 2-3 years. Before arriving at the zoo, he was used for commercial purposes – for photo shoots in the Kolsai

Kolderi State National Nature Park in the Almaty region. Medical assistance was sought in December 2023. Diagnosis: pododermatitis.

Steppe eagle №3, nicknamed “Gorny,” age 11. Born in 2014. Received from the public in 2016, previously used by a private individual for hunting. First visit to the veterinary clinic in spring 2022. Diagnosis: pododermatitis, ill for 2-3 years (Fig 1).

Fig 1. External appearance of lesions on the footpads of steppe eagles

Medical history revealed that all three eagles were kept in the same aviary. The aviary was an open-type cage with one solid wall and partial rain cover. The remaining walls and 50% of the ceiling were made of wire mesh. The aviary had perches made of thick branches 7-10 cm in diameter (Fig 2).

Fig 2. Conditions for keeping wild eagles in captivity

The birds were fed red meat purchased from the market and given freshwater ad libitum. Feeders and drinking bowls were placed on the ground with free access. At the time of inspection, the bedding was found to be wet, with water seeping out when pressed, as it had rained and snowed during the night. At the time of capture and inspection, the eagles remained aggressive and anxious, which is characteristic of the behavior of this species of wild bird. The inspection revealed that the birds' bodies were wet, their feathers were damp and ruffled, and the structure of the feathers was damaged. The eagles were generally depressed, had a reduced appetite, reacted weakly to their surroundings, and croaked softly.

The young eagles flew up to their perch, while the old eagle made unsuccessful attempts to fly away when people approached, moving away from them and huddling in a corner. The bird limped when moving and often sat down. Upon examination of the adult eagle, an unpleasant sticky body crumpled down and cold skin were noted.

According to veterinary specialists, the affected areas on the soles of the birds' feet were treated with Levomekol ointment and then bandaged. The treatment had no effect, the positive dynamics were short-lived, the affected area increased and bled regularly. After rain or the formation of puddles in the enclosure, the suppuration process on the soles of the eagles' feet intensified, and the condition of the wild birds deteriorated.

A visual examination revealed that the gauze bandage was wet, dirty and saturated with liquid from the litter. Under the bandage, inflammation of the horny coverings of the soles (the horny corium of the foot) was detected. The skin was thin and, when attempts were made to remove the bandage or take a sample of biomaterial, it cracked easily and bled for a long time.

Biological material samples were taken from the affected area of the bird's foot pads by scraping the skin and cutting away necrotic tissue on day 0 after properly sterilizing the affected area with 70% alcohol and pulling out 1-2 sticky ruffled feathers from the body [30]. Bedding samples were taken from the location of the birds, near the feeder.

Immediately after collection, the biomaterial samples were placed on prepared dry CompactDry YM culture media, and surface cultivation was carried out for 1 to 3 days [31].

The isolation and identification of pure cultures, as well as the analysis of cultural and biochemical properties, were carried out in the microbiology laboratory of the Engineering Center for Organic Agricultural Technologies at the S. Seifullin Kazakh Agricultural Technical Research University. Isolating of pathogens was carried out on Sabouraud medium, surface cultivation was carried out for 10 days at 28 °C until characteristic colonies formed [32, 33].

Microscopic analysis of the morphological structures of microfungi was performed using an AxioScope A1 trinocular transmitted light microscope (Zeiss, Germany) at ×40 magnification, and microstructures were studied using an Olympus microscope [34].

The presence of keratinolytic properties of micromycetes was detected in a hair perforation test [32, 35] and sensitivity to antifungal drugs [36] was determined using the CLSI method [37].

For molecular identification of the species, a small amount of cell biomass was selected from each culture and homogenized. The homogenate was placed in an extraction buffer (0.1 M Tris (pH 8), 0.01 M EDTA, 2% SDS) with the addition of Proteinase K (20 ng/µL), 10% TAV, and 5 M NaCl. Next the extraction was performed with a mixture of chloroform and isoamyl alcohol (24:1). Then, the DNA was precipitated with ethanol, purified, dissolved in 1×TE buffer and stored

at -20°C for subsequent analysis. The amount and purity of the isolated DNA were determined by measuring the optical density at 260 and 280 nm using a NanoDrop 2000 device (Thermo Scientific, USA) [38].

PCR amplification of the variable ITS region of rDNA was performed using universal primers ITS1 (5′TCCGTAGGTGAACCTGCGG3′) and ITS4 (5′TCCTCCGCTTATTGATATGC3′). Amplification was performed using HS-Taq PCR Biomasters (2×) (Biolabmix LLC, Novosibirsk, Russia), with 10 pmol of each primer and 100 ng/μl of larval DNA as a template. The reaction was performed with an initial denaturation at 95°C for 5 min, followed by 35 cycles consisting of: denaturation at 95°C for 30 sec, annealing at 57°C for 30 sec and elongation at 72°C for 60 sec. Final extension at 72°C for 7 min. The results of the PCR analysis were interpreted by gel electrophoresis and documented using a GelDoc XR+ transilluminator (Bio-Rad, Hercules, CA, USA). Sequencing was performed on a SeqStudio genetic analyzer (Thermo Fisher Scientific Applied Biosystems, USA) [39].

The obtained nucleotide sequences were manually checked and edited using BioEdit software (version 7.0) before analysis using BLAST against reference sequences in GenBank (https://www.ncbi.nlm.nih.gov/).

Results

By the end of the first day, multiple blue colonies (yeast growth) and rare brown-green colonies (mold growth) were observed on the CompactDry YM medium (Fig 3).

Fig 3. Growth of microfungal colonies on «CompactDry YM» media

Various molds and yeasts were observed growing on Saburo media (Fig 4).

Fig 4. Primary cultures of microfungi on Sabouraud medium.
1 - *Fusarium* spp., 2 - *Candida* spp., 3 - *Penicillium* spp., 4 - *Mucor* spp., 5 - *Aspergillus* spp.

Analysis of the primary culture revealed the growth of yeast characteristics of *Candida* spp. The cultural and morphological properties of yeast strains isolated from wild eagles and litter are shown in Fig 5.

№17.24.1 eagle №17.24.2 eagle №17.24.3 eagle

Fig 5. Appearance of colonies on media and yeast isolate cells

Molecular genetic identification of isolates allowed strains to be identified as *Candida metapsilosis* and nucleotide sequences of isolates to be deposited in the GenBank database of the NCBI (USA).

Biochemical analysis showed that *C. metapsilosis* yeast strains have high saccharolytic activity, breaking down all carbohydrates in the sequence

maltose>sucrose>lactose>mannitol>glucose (Fig 6a), weak proteolytic activity, selectively breaking down hemoglobin, gelatin, and peptone (Fig 6c). High keratinophilic (Fig 6b) and urease (Fig 6d) activity was noted in all three strains.

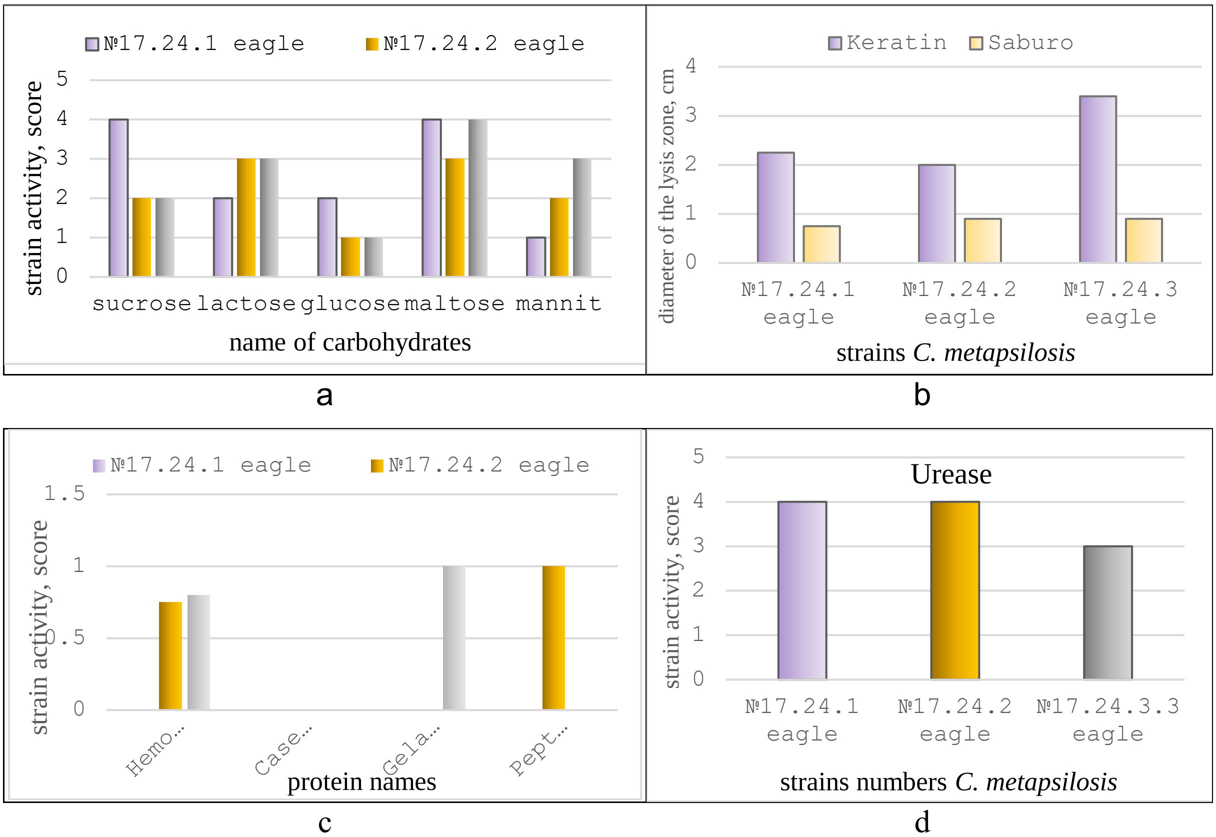

Fig 6. Biochemical activity of *C. metapsilosis* strains. a – keratinophilic, b – saccharolytic, c – proteolytic, d – urease

Sensitivity testing of strains to antifungal drugs showed that the yeasts are sensitive to several drugs. All three strains are highly sensitive to ketoconazole, two are sensitive to clotrimazole and fluconazole, and one is sensitive to nystatin. All strains are weakly sensitive to amphotericin (Fig 7).

Degree of sensitivity, cm	
sustainable	0-0,6
low-sensitive	0,7-1,5
sensitive	1,6-2,4
high-sensitive	2,5 and more

Disc submission and scoring

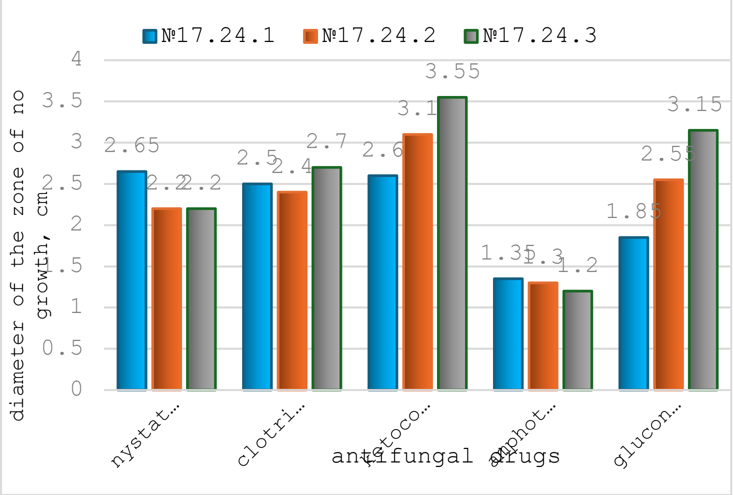

Sensitivity of strains to antifungal drugs

Fig 7. Scheme for introducing and recording the sensitivity of *C. metapsilosis* strains to antimycotics

Treatment with Clotrimazole 1% ointment (Sintez, PAO, Russia) for external use once a day was prescribed, alternating with Fluconazole ointment (Werteks, Russia) until a positive effect was achieved. After two weeks, Humic-Salve liniment (BioNanoPreparat, Kazakhstan) was applied topically to the paw pads to accelerate the epithelialization of the wound surface. To increase the natural resistance of the bird's body, the drinking feed supplement Gumka-KZ (Kaztechnougol Scientific and Production Association LLP & BioNanoPreparat, Kazakhstan) with drinking water at a dose of 0.15 ml per 1 kg for a month. The treatment led to recovery, with positive dynamics in the healing of lesions (Fig 8).

Fig 8. Stages of wound healing on the paw pads of steppe eagles

The effectiveness of treatment was monitored by mycological examination of biomaterial samples taken from birds. Clinical recovery of eagles occurred on day 15, and mycological recovery (negative test results) on day 21. Relapses were

observed after rain in an adult eagle nicknamed “Stary” which were successfully treated with liniment after 3-5 days.

To prevent yeast pododermatitis in wild eagles recommended to replace bedding in a timely manner, especially after rain; treating perches and floors with universal disinfectants or antifungal drugs; giving eagles the Gumka-KZ feed supplement for 10 days a month for six months to boost their immunity.

Discussion

The genus *Candida* includes fungal species of yeast-like organisms. Of the approximately 200 species belonging to the genus, only 20 species are most isolated from clinical samples [40, 41]. Although *C. albicans* is considered the main causative agent of candidiasis in human and veterinary medicine, other *Candida* species are currently most commonly detected [42].

Various authors have mentioned the isolation of pathogens such as *Candida parapsilosis*, *Candida orthopsilosis, Candida metapsilosis, Candida krusei*, *Candida lusitaniae, Candida glabrata* and *Candida tropicalis* from humans [40, 43-47].

In this study, we isolated and characterized three strains of yeast-like fungi that caused chronic yeast pododermatitis in wild eagles from the Kazakh population that had been living in captivity for a long time. The strains were identified as *C. metapsilosis* by DNA sequence analysis and deposited in GenBank under numbers PV945930.1, PV981955.1, and PV945931.1. Comparative analysis of nucleotide sequences with the closest homologues in

GenBank showed maximum homology of the nucleotide sequences of *C. metapsilosis* isolated from wild eagles of the Kazakh population with the culture from the type of material *C. parapsilosis* KC777375.1 used to evaluate the pathogenicity of the *Candida parapsilosis* complex in vivo in a mouse model of disseminated candidiasis (Mexico). Thus, strain PV945930.1 showed a 99.28% match (750 bp), strain PV945931.1 showed a match of 95.88% (669 bp), and strain PV981955.1 showed a match of 94.55% (https://blast.ncbi.nlm.nih.gov/Blast.cgi). At the same time, the GenBank database found 106 matches for strain PV945931.1 with 763 nucleotide pairs, strain PV945930.1 – 105 matches with 769 n.p., and strain PV981955.1 had 113 matches across 442 n.p.

A search for *Candida metapsilosis* nucleotides in BLAST with 769, 763, and 442 nucleotides allowed us to find previously deposited single yeast sequences isolated from animals: in Italy from cattle (PV416737.1) and in Brazil from the common coati (MF797776.1), in Peru from red paco fish (MZ540270.1). There were more strains of *C. metapsilosis* identified from humans with skin or skin appendage lesions: from skin in Malaysia (MH715400.1), from skin granuloma in China (PQ626156.1), from nails in the Netherlands (MK561031.1) and Egypt (KU200445.1), from patients diagnosed with onychomycosis in Shanghai, China (KJ816926.1, KJ816917.1, KJ816927.1). The largest number of *C. metapsilosis* strains was identified in patients with internal organ damage,

disseminated candidiasis, candidemia, and other pathologies – 53 sequences (https://blast.ncbi.nlm.nih.gov/Blast.cgi).

Noteworthy is the maximum homology with the nucleotide sequences of *C. metapsilosis* strains isolated from the intestinal microbiome of fish (MZ540270.1), marine sediments in India (JQ665419.1), wastewater in Brazil (QR731586.1) and the sea surface and underlying waters in Taiwan (FJ515169.1, FJ515199.1, FJ515200.1). This may indicate the high resistance of *C. metapsilosis* strains in the litter of the room where the eagles lived and prove that the birds were reinfected over a long period of time. *C. metapsilosis* strains inhabiting the litter are likely to be highly adaptable and resistant, which may contribute to prolonged reinfection of birds in aviary conditions. This may be related to thermotolerance and the ability to form biofilms, which allows yeast cells to remain viable for long periods [48].

The uniqueness of *C. metapsilosis* isolation from wild eagles of the Kazakh population, which have been living in captivity for a long time, explains the difficulties in diagnosis and problems with the treatment of pododermatitis. We were unable to trace the epidemiological chain of infection of wild eagles with *C. metapsilosis* in the breeding facility. A possible cause was a chronic infection of the footpads in an old steppe eagle nicknamed "Stary" which had been kept in captivity for a long time under inappropriate conditions. Its medical history revealed that the bird had been kept in condition with an improperly constructed perch. Narrow branches from the top of a felled tree were used for this purpose.

It is likely that the resulting increase in pressure on the small support surface of the feet led to microcracks and the introduction of the pathogen. The disease was exacerbated by improper treatment without the use of antifungal drugs.

The clinical signs of pododermatitis in wild birds were characteristic, distinguished by a prolonged course of the disease with periodic discharge of purulent contents, which allowed veterinarians to diagnose «bacterial pododermatitis». This was also mentioned by other authors, who reported that wild birds in captivity often suffer from prolonged traumatic pododermatitis associated with improper husbandry and that the main causative agent of the disease is most often *S. aureus* [25]. There are publications describing *E. coli*, *Pasteurella* spp., *Klebsiella* spp., *Clostridium* spp., *Corynebacterium* spp., *Bacillus* spp., *Diplococcus* spp., *Nocardia* spp., *Actinobacillus* spp., *Actinomyces* spp., *Aeromonas* spp., *Proteus* spp. and *Pseudomonas* spp. as causative agents of bacterial pododermatitis in wild and domestic birds [16, 49].

The isolation of *Candida* spp. micromycetes from affected areas in birds has been reported repeatedly [26-27]. There are known cases of *Candida* spp. infection in canaries (Serinus canaria), macaws (Ara macao) and cockatoos (Nymphicus hollandicus), pigeons (Columba livia domestica), geese (Anser/Branta sp.), guinea fowls (Numida meleagris), pheasants (Phasianus colchicus), quails (Coturnix coturnix), parrots (Psittacus sp.) and other birds [20, 21, 28, 50-53].

Reports of fungal infections in captive birds mention the role of *Candida albicans* in skin lesions in hatched hornbill chicks and adult lapwings and curlews [14]. *C. albicans* more often cause cutaneous candidiasis or fungal skin infection which can often occur in connection with systemic infections or weakened immunity. The cutaneous form of the infection can also spread among birds in the presence of infected mites and the use of dirty feeding utensils [5, 54]. There have been reports of candidiasis developing because of poor housing conditions and uncleanliness [20, 21, 55]. There is a known case of systemic candidiasis caused by *C. parapsilosis* in a 20-year-old blue-winged Amazon with lameness [28]. There are no reports of the role of *Candida metapsilosis* in lesions of the footpads of wild eagles living in the wild or in captivity. Our research has expanded the list of pathogens of wild eagles living in captivity in Kazakhstan and proposed a treatment regimen.

The pathogen causing chronic yeast pododermatitis in wild eagles from the Kazakh population, which have been living in captivity for a long time, was identified based on morphological characteristics in vitro: the shape and color of colonies and reverse and the size and shape of yeast cells. Attention was paid to the speed and nature of growth tube formation on agarized media, the ability to assimilate urea, proteins and carbohydrates and to decompose keratin. Joshi KR and co-authors pointed out the need to take these characteristics into account when differentiating pathogenic yeasts from non-pathogenic yeast pathogens [56].

Mycological diagnosis allowed the pathogens to be classified as belonging to the genus *Candida*. The pure culture of all three strains of *C. metapsilosis* was distinguished by characteristic features. On Sabouraud agar, the colonies grew rapidly, forming uniformly rounded, smooth, creamy, yellowish colonies with a diameter of 1.0–2.0 mm, which became matte with age and formed zones separated by deep furrows, growing unevenly to 1.0×1.5–1.5×1.8 cm in diameter. It should be noted that all strains of *C. metapsilosis* were completely undemanding in terms of nutrient medium, grew well and formed characteristic colonies on Sabouraud dextrose agar at a temperature of – 28 ± 1 °C. During primary isolation, soft velvety zones of young colonies with pink-red pigment formed around the central typical creamy light cream colony.

Vegetative structures identified under a microscope in strain *C. metapsilosis* №17.24.1 – pseudomycelium (pseudohyphae), rudimentary pseudomycelium, actively budding cells; in strains *C. metapsilosis* №17.24.2 and *C. metapsilosis* №17.24.3, budding cells were identified, no mycelial structures were identified. The fungi had uniform yeast cells of round, oval and ellipsoidal shape, with clearly visible differences in smears (Fig 5). The budding cells of all strains had both single daughter cells and cells attached to the mother cell, being practically the same size. A similar morphology of *C. metapsilosis* yeast has been described by Gómez-Gaviria M. et al. and other authors [42].

The literature mentions a controversial issue regarding the ability of *C. metapsilosis* to form pseudohyphae. Several studies report on the formation of

pseudohyphae by *C. metapsilosis* strains [57, 58]. An analysis of 93 different isolates of *C. parapsilosis* sensu lato conducted by Pryszcz LP et al. (2015) revealed that *C. metapsilosis* does not possess such morphology [59]. It should be noted that the two strains we isolated from eagles also did not have the ability to form pseudohyphae. However, strain *C. metapsilosis* №17.24.1, which was obtained from an old steppe eagle that had been ill for many years, formed clearly visible pseudohyphae and rudimentary pseudomycelium. We believe that this is due to genetic differences in the analyzed fungal strain, which affected the phenotypic characteristics (larger colony diameter, folding, deep furrows), including morphological changes (pseudomycelium (pseudohyphae), rudimentary pseudomycelium, actively budding cells). We exclude the influence of cultivation conditions such as temperature, pH and nutrient medium composition, which some researchers mention [42], since cultivation was carried out under identical conditions.

All three strains were characterized by high keratinolytic and urease activity, indicating the potential pathogenicity of the pathogens [60, 61, 62]. The presence of saccharolytic and proteolytic properties indicates the high potential for nutritional adaptability of the isolates and their ability to break down various substrates in the external environment, which allows them to remain viable in the external environment for a long time and acquire virulent and pathogenic properties during microorganism-host interactions [63].

The sensitivity of *C. metapsilosis* strains to all antifungal drugs included in the study was determined. All three strains were highly sensitive to ketoconazole, two were sensitive to clotrimazole and fluconazole and one was sensitive to nystatin. At the same time, all strains were weakly sensitive to amphotericin B. The ambiguous sensitivity of *Candida* strains, including *C. metapsilosis*, to various antimycotics has been reported previously [64, 65]. Considering the classic treatment regimens for candidiasis, the availability of drugs in city pharmacies and sensitivity, clotrimazole and fluconazole were recommended for treatment, which had also been used previously for such purposes [54, 66]. In conclusion, we report that the correctly chosen tactics and strategy of therapeutic measures allowed us to completely cure steppe eagles from candidal pododermatitis. The prescription of antimycotics in combination with humic ointment and feed additives led to the recovery of steppe eagles, and the use of the correct perch prevented further reinfection of wild birds with *C. metapsilosis* by normalizing the natural resistance of the organism.

Conclusion

Candida metapsilosis was first isolated from wild steppe eagles of the Kazakh population living in captivity, diagnosed with bacterial pododermatitis, and from bedding, establishing their etiological role.

Molecular genetic identification of the strains of pathogens revealed their identity with *C. metapsilosis* sequences at 99.28-94.25%.

C. metapsilosis strains were characterized by pronounced pathogenicity, high keratinophilic and urease activity. The strains cleaved maltose, glucose, and, to a lesser extent, lactose, mannitol, and glucose; they had weak proteolytic activity, selectively cleaving hemoglobin, gelatin, and peptone.

C. metapsilosis strains showed varying sensitivity to antifungal drugs. All three strains were highly sensitive to ketoconazole, two were sensitive to clotrimazole and fluconazole and one was sensitive to nystatin. At the same time, all strains were weakly sensitive to amphotericin B.

Treatment of pododermatitis in wild eagles with a combination of antifungal drugs (single dressing once a day with ointment applied to the wound for 2 weeks), humic liniment to enhance tissue regeneration with an antimicrobial effect (single dressing once a day with a napkin impregnated with the drug applied to the wound for 10 days), and a humic feed supplement resulted in complete recovery within a month.

Authors' contributions:

Conceptualization, R.S., E.K.; methodology, E.K.; software, R.S., M.T., G.B.; validation, E.K., G.B.; formal analysis, G.B., A.N.; research, E.K., G.B., N.T., A.N.; resources, E.K.; data processing, E.K., N.T.; writing – preparation of the original draft, E.K.; writing – review and editing, E.K. and A.N.; visualization, G.B., A.N.; author supervision, E.K., A.N.; project administration, E.K.;

fundraising, E.K. All authors have reviewed and agreed with the published version of the manuscript.

Declaration of competing interest

There are none.

Acknowledgements

The study was funded by grant AP23489001 (2024-2026) from the Ministry of Education and Science of the Republic of Kazakhstan. The authors would like to express their gratitude to Kiyan V.S., Head of the Laboratory of Biodiversity and Genetic Resources at the National Center for Biology of the Republic of Kazakhstan and to project participants Jazina K., Uakhit R.S., practicing veterinary specialists for their assistance in sampling, conducting laboratory studies and species identification of pathogens.

References

1 Schwehn R, Engelke E, Seiler C, Fischer D, Seifert H, Pfarrer C, et al. The comparative anatomy of the metatarsal foot pad in eight species of birds of prey and owls with regard to the development of pododermatitis. Vet Sci. 2025;12(5):498. doi:10.3390/vetsci12050498

2 Cooper JE. Foot conditions. In: Cooper JE, editor. Birds of prey: health and disease. 3rd ed. Oxford (UK): Blackwell Science Ltd; 2002. p. 121–131.

3 Harcourt-Brown NH. Bumblefoot. In: Samour J, editor. Avian medicine. 2nd ed. Oxford (UK): Mosby Elsevier; 2008. p. 209–215.

4 Heidenreich M. The musculoskeletal system. In: Heidenreich M, editor. Birds of prey: medicine and management. Oxford (UK): Blackwell Science Ltd; 1997. p. 203–218.

5 Müller MG, Wernery U, Kösters J. Bumblefoot – a historical introduction to a common falcon foot disease. Falco. 2002;19:14–18.

6 Lierz M. Aspects of the pathogenesis of bumblefoot in falcons. In: Proc 7th Conf Eur Assoc Avian Vet. 2003 Apr 22–26; Tenerife, Spain. p. 178–184.

7 Müller MG. Studies on bumblefoot in falcons used for hunting in the United Arab Emirates [dissertation]. Munich (Germany): Ludwig-Maximilian University; 1999.

8 Müller MG, Wernery U, Kösters J. Bumblefoot and lack of exercise among wild and captive-bred falcons tested in the United Arab Emirates. Avian Dis. 2000;44:676–680. doi:10.2307/1593110

9 Beaufrère H, Laniesse D. Medicine of Strigiformes. In: Speer BL, editor. Current therapy in avian medicine and surgery. St. Louis (MO): Elsevier; 2016. p. 566–581.

10 Rodriguez-Lainz AJ, Hird DW, Kass PH, Brooks DL. Incidence and risk factors for bumblefoot (pododermatitis) in rehabilitated raptors. Prev Vet Med. 1997;31:175–184. doi:10.1016/S0167-5877(96)01137-3

11 Poorbaghi SL, Javdani M, Nazifi S. Surgical treatment of bumblefoot in a captive golden eagle (*Aquila chrysaetos*). Vet Res Forum. 2012;3:71–73.

12 Parsad A, Shukhla SK, Arora N, Kumar P. Bumble foot in geese and its successful management. Vet Pract. 2010;11:9.

13 Gentz EJ. *Fusobacterium necrophorum* associated with bumblefoot in a wild great horned owl. J Avian Med Surg. 1996;10:258–261.

14 Daoust PY, Wadowska D, Kibenge F, Campagnol RP, Latimer KS, et al. Proliferative pododermatitis associated with virus-like particles in a northern gannet. J Wildl Dis. 2000;36:378–382. doi:10.7589/0090-3558-36.2.378

15 Reidarson TH, Mcbain J, Burch L. A novel approach to the treatment of bumblefoot in penguins. J Avian Med Surg. 1999;13:124–127.

16 Pad dermatitis associated with osteomyelitis in a mute swan (*Cygnus olor*). Am J Anim Vet Sci. 2018;13(2):66–69. doi:10.3844/ajavsp.2018.66.69

17 Harcourt-Brown N. Birds of prey: anatomy, radiology and clinical conditions of the pelvic limb. Lake Worth (FL): Zoological Education Network Inc.; 2000.

18 Bailey T, Lloyd C. Raptors: disorders of the feet. In: Chitty J, Lierz M, editors. BSAVA manual of raptors, pigeons and passerine birds. Gloucester (UK): BSAVA; 2008. p. 176–189.

19 Fischer D. Bumblefoot – a common problem in captive birds. Wildtierzeit. 2011;1:24–28.

20 Friend M. Fungal diseases. In: Friend M, Franson JC, editors. Field manual of wildlife diseases. Madison (WI): USGS; 1999. p. 128–136.

21 Kumar SK, Kumar MR, Mahalakshmi V, Kavitha S. Candidiasis in a parakeet – an avenue to zooanthroponosis. J Anim Health Prod. 2017;5:85–88.

22 Doneley R. Avian medicine and surgery in practice. London (UK): Manson Publishing; 2010.

23 Remple JD, Al-Ashbal AA. Raptor bumblefoot: histopathology and pathogenesis. In: Redig PT, Cooper JE, Remple JD, Hunter DB, editors. Raptor biomedicine. Minneapolis (MN): University of Minnesota Press; 1993. p. 92–98.

24 Cooper JE. Foot conditions. In: Veterinary aspects of captive birds of prey. Gloucestershire (UK): The Standfast Press; 1978. p. 97–111.

25 Oaks JL. Immune and inflammatory responses in falcon staphylococcal pododermatitis. In: Raptor biomedicine. Minneapolis (MN): University of Minnesota Press; 1993. p. 72–87.

26 Ritchie BW, Harrison GJ, Harrison LR, editors. Mycoses. In: Avian medicine: principles and application. Lake Worth (FL): Wingers Publishing; 1994. p. 998–1006.

27 Pitarch A, Gil C, Blanco G. Oral mycoses in avian scavengers exposed to antibiotics from livestock farming. Sci Total Environ. 2017;605–606:139–146. doi:10.1016/j.scitotenv.2017.06.144

28 Goodman G, Widenmeyer JC. Systemic *Candida parapsilosis* in a blue-fronted Amazon. Proc Assoc Avian Vet. 1986:105–119.

29 Ugochukwu ICI, Aneke CI, Sani NA, Omeke JN, Anyanwu MU, Odigie AE, et al. Important mycoses of wildlife. Animals (Basel). 2022;12(15):1874. doi:10.3390/ani12151874

30 National Research Council (US). The development of science-based guidelines for laboratory animal care. Washington (DC): National Academies Press; 2004.

31 Kodaka H, et al. Comparison of the Compact Dry YM with the FDA BAM method for enumeration of yeasts and molds in fruit-based products. *J AOAC Int.* 2006;89(1):127–138.

32 Sutton D, Fothergill A, Rinaldi M. Determinant of pathogenic and conditionally pathogenic fungi. Moscow: Mir; 2001.

33 Ajello L, Georg LK, Kaplan W, Kaufman L. *Laboratory manual for medical mycology.* Atlanta (GA): CDC; 1966.

34 Robert R, Pihet M. Conventional methods for the diagnosis of dermatophytosis. Mycopathologia. 2008;166:295–306. doi:10.1007/s11046-008-9106-3

35 Kidd S, Halliday C, Alexiou H, Ellis D. Descriptions of medical fungi. 3rd ed. Adelaide: NMRC; 2016.

36 Clinical and Laboratory Standards Institute. Reference method for broth dilution antifungal susceptibility testing of filamentous fungi. CLSI M38-A2. Wayne (PA); 2008.

37 Clinical and Laboratory Standards Institute. *Method for antifungal disk diffusion susceptibility testing of yeasts.* CLSI M44-A2. Wayne (PA); 2009.

38 McKiernan J, Danielson K. Evaluation of phenol–chloroform DNA extraction methods for viromes. PLoS One. 2015;10:e0115960. doi:10.1371/journal.pone.0115960

39 White TJ, Bruns T, Lee S, Taylor J. Amplification and sequencing of fungal rRNA genes. In: *PCR protocols.* San Diego (CA): Academic Press; 1990. p. 315–322.

40 Pfaller MA, Diekema DJ, Procop GW, Rinaldi MG. Multicenter comparison of VITEK 2 antifungal testing. J Clin Microbiol. 2007;45(11):3522–3528. doi:10.1128/JCM.00403-07

41 Bouopda Tamo SP. Candida infections: clinical features, diagnosis and treatment. Infect Dis Clin Microbiol. 2020;2:91–102. doi:10.36519/idcm.2020.0006

42 Gómez-Gaviria M, García-Carnero LC, Baruch-Martínez DA, Mora-Montes HM. *Candida metapsilosis*. Infect Drug Resist. 2024;17:171–185. doi:10.2147/IDR.S448213

43 Hani U, Shivakumar HG, Vaghela R, Osmani RA, Shrivastava A. Candidiasis. Infect Disord Drug Targets. 2015;15(1):42–52.

44 Arendrup M, Horn T, Frimodt-Møller N. In vivo pathogenicity of *Candida* spp. Infection. 2002;30(5):286–291.

45 Pfaller MA, Neofytos D, Diekema DJ, et al. Epidemiology of candidemia. Diagn Microbiol Infect Dis. 2012;74(4):323–331.

46 Pfaller MA, Diekema DJ, Mendez M, et al. *Candida guilliermondii*. J Clin Microbiol. 2006;44(10):3551–3556.

47 Pfaller MA, Jones RN, Messer SA, Edmond MB, Wenzel RP. National surveillance of candidemia. Diagn Microbiol Infect Dis. 1998;30(2):121–129.

48 Lattif AA, Mukherjee PK, Chandra J, Swindell K, Lockhart SR, Diekema DJ, Pfaller MA, Ghannoum MA. Characterization of biofilms formed by *Candida parapsilosis*, *C. metapsilosis*, and *C. orthopsilosis*. Int J Med Microbiol. 2010;300(4):265–270.

49 Heidemann Olsen R, Christensen H, Kabell S, Bisgaard M. Pododermatitis in egg layers. Avian Pathol. 2018;47(3):281–285.

50 Rhimi W, Sgroi G, Aneke CI, Annoscia G, Latrofa MS, Mosca A, et al. Wild boar as reservoir of zoonotic yeasts. Mycopathologia. 2022;187:235–248.

51 Donnelly KA, Wellehan JFX, Quesenberry K. Non-*albicans Candida* in birds. J Avian Med Surg. 2019;33:413–418.

52 Marruchella G, Todisco G, D'Arezzo S, Di Guardo G, Paglia MG. *Candida albicans* myocarditis in canary. J Avian Med Surg. 2011;25:205–209.

53 Moretti A, Piergili Fioretti D, Boncio L, Pasquali P, Del Rossi E. *Candida rugosa* in turkeys. J Vet Med B. 2000;47:433–439.

54 Hoppes MH. Mycotic diseases of pet birds. MSD Vet Manual. 2024.

55 Redig P. Fungal diseases. In: Samour J, editor. Avian medicine. Edinburgh: Elsevier; 2003. p. 275–291.

56 Joshi KR, Bremner DA, Parr DN, Gavin JB. Identification of pathogenic yeasts. J Clin Pathol. 1975;28(1):18–24.

57 Lackey E, Vipulanandan G, Childers DS, Kadosh D. Morphological regulation in *Candida*. Eukaryot Cell. 2013;12(10):1356–1368.

58 Melo AS, Bizerra FC, Freymüller E, Arthington-Skaggs BA, Colombo AL. Biofilms in *Candida*. Med Mycol. 2011;49(3):253–262.

59 Pryszcz LP, Nemeth T, Saus E, et al. Hybridization in *Candida metapsilosis*. PLoS Genet. 2015;11(10):e1005626.

60 Papini R, Mancianti F. Enzymatic activity of *Microsporum canis*. Mycopathologia. 1996;132:129–132.

61 Lerm B, Kenyon C, Schwartz IS, Kroukamp H, de Witt R, Govender NP, et al. Emergomyces africanus. FEMS Yeast Res. 2017;17(7):fox069.

62 Okafor JI, Ngwogu AC. Keratinolytic activity of dermatophytes. J Commun Dis. 2001;32:300–305.

63 Bras G, Satala D, Juszczak M, Kulig K, Wronowska E, Bednarek A, et al. Secreted aspartic proteinases in *Candida*. Int J Mol Sci. 2024;25(9):4775.

64 Bonfietti LX, Martins MDA, Szeszs MW, Pukiskas SBS, Purisco SU, Pimentel FC, et al. *Candida parapsilosis* complex. J Med Microbiol. 2012;61:1003–1008.

65 Chen YC, Lin YH, Chen KW, Lii J, Teng HJ, Li SY. *Candida parapsilosis* complex in Taiwan. Diagn Microbiol Infect Dis. 2010;68(3):284–292.

66 Issa AJ, Muhammed AI, Mohamed M, Jabar R. Clotrimazole treatment of fungal skin infections. Iraqi J Ind Res. 2023;10(1):131–137.

Click here to access/download;Figure;Fig 1 Steppe eagle 1.jpg

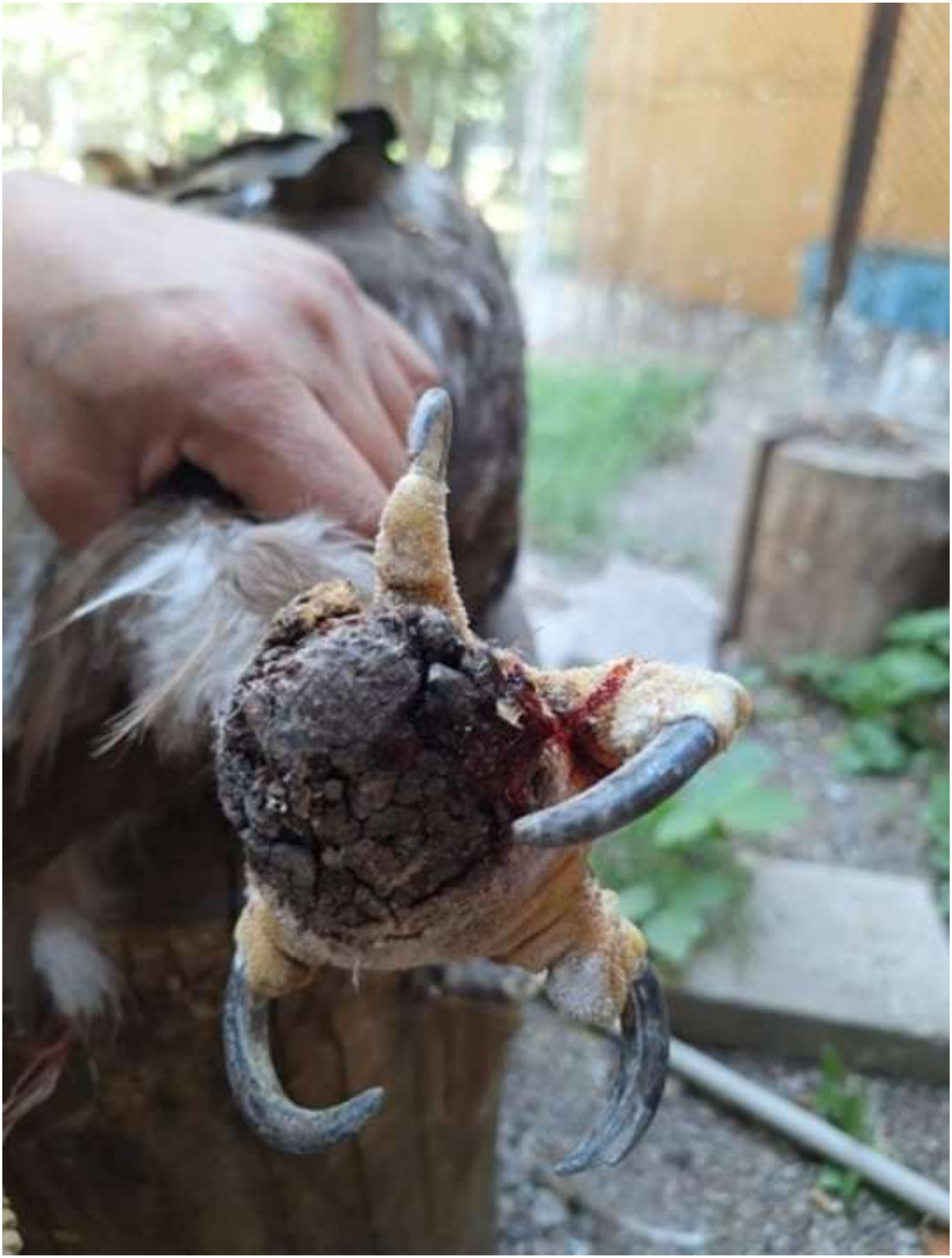

Click here to access/download;Figure;Fig 1 Steppe eagle 2.jpg

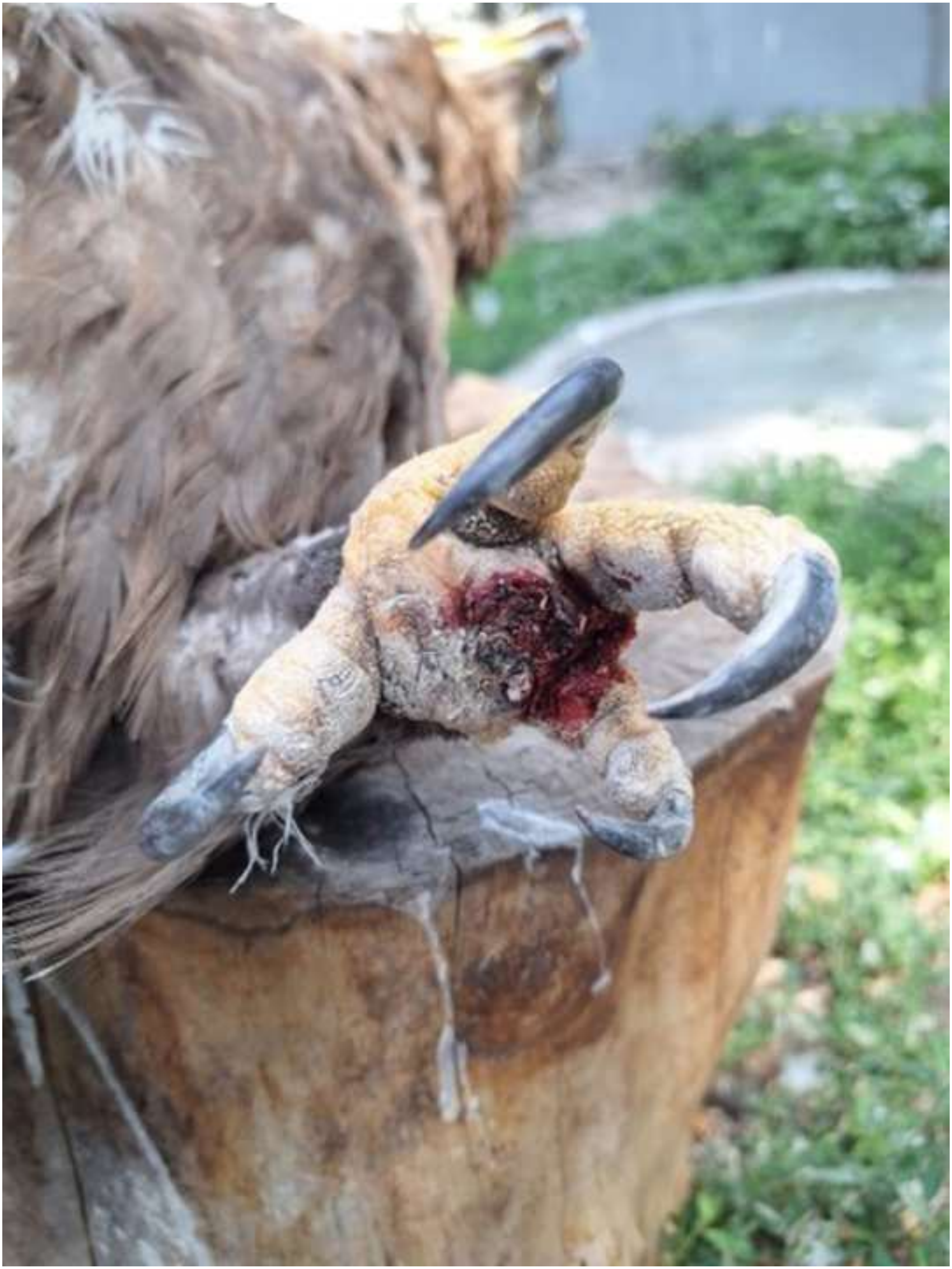

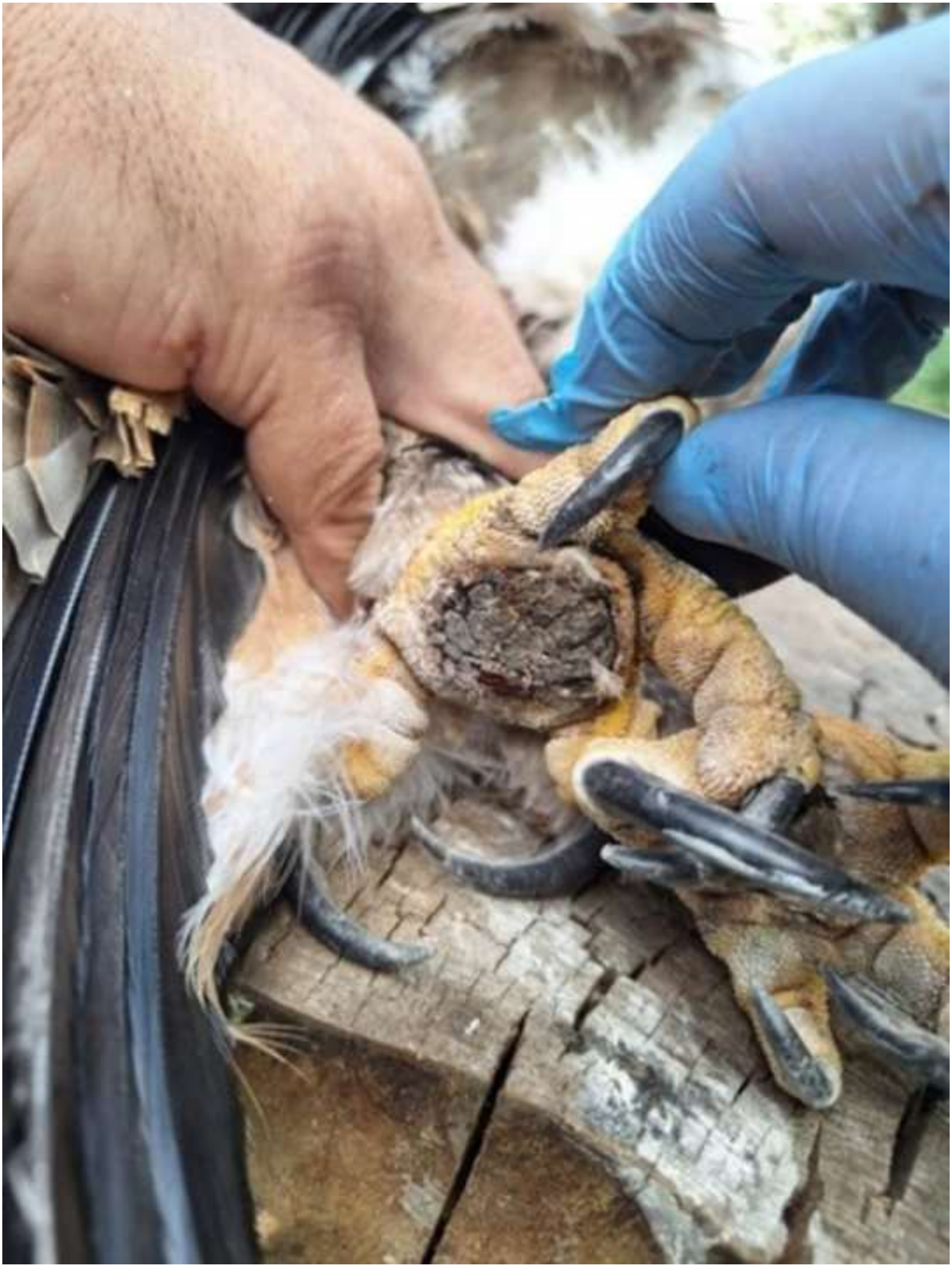

Click here to access/download;Figure;Fig 2 (1).jpg

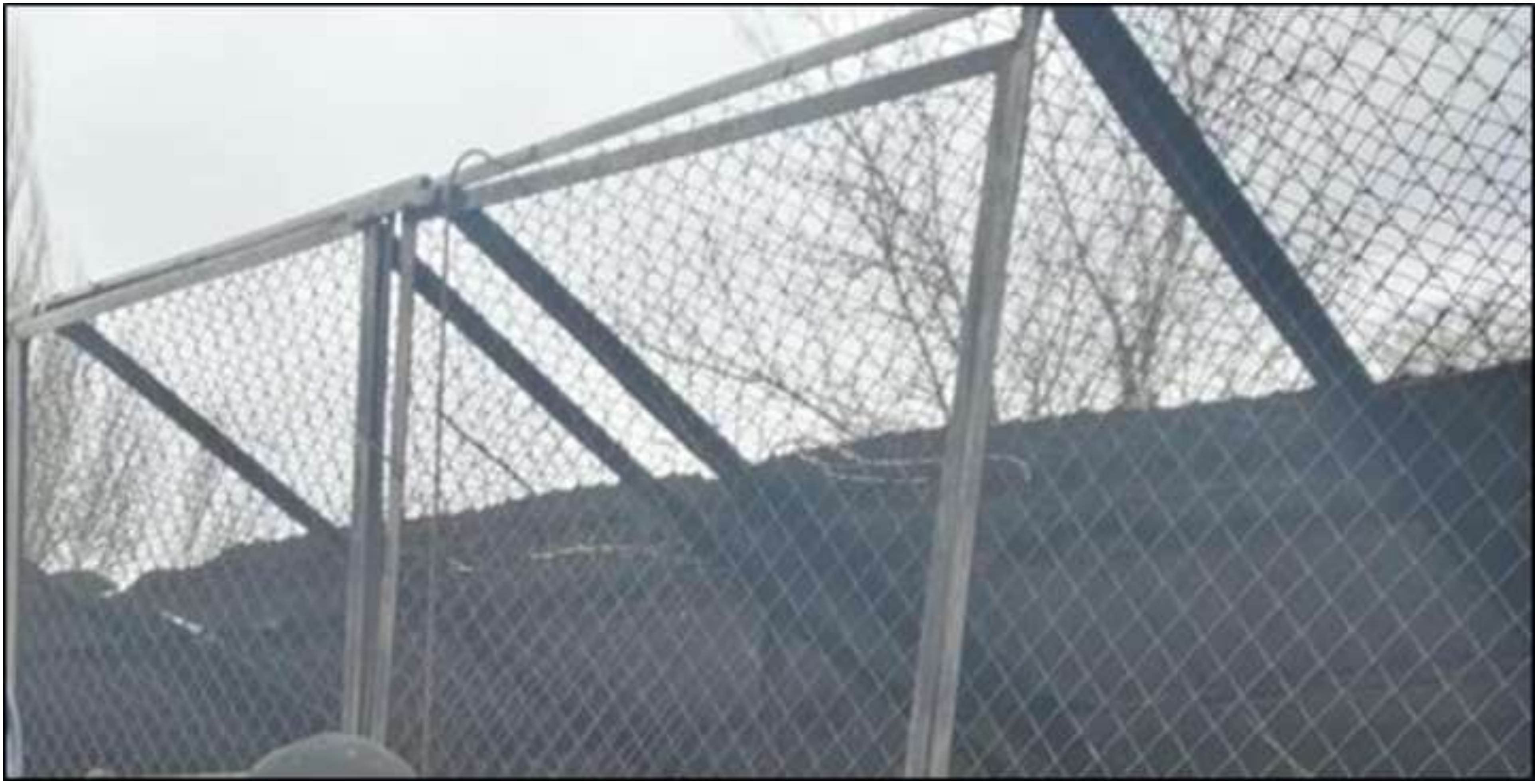

Click here to access/download;Figure;Fig 2 (2).png

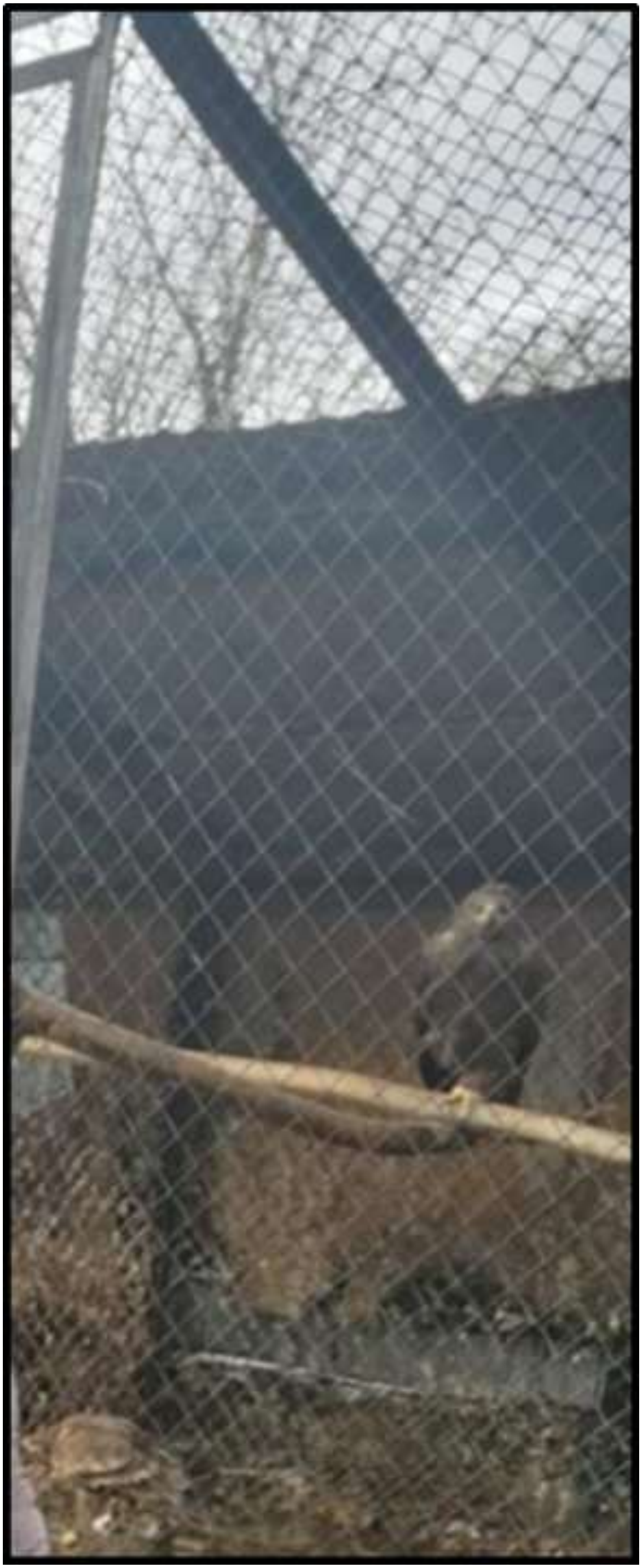

Click here to access/download;Figure;Fig 3 (1).jpg

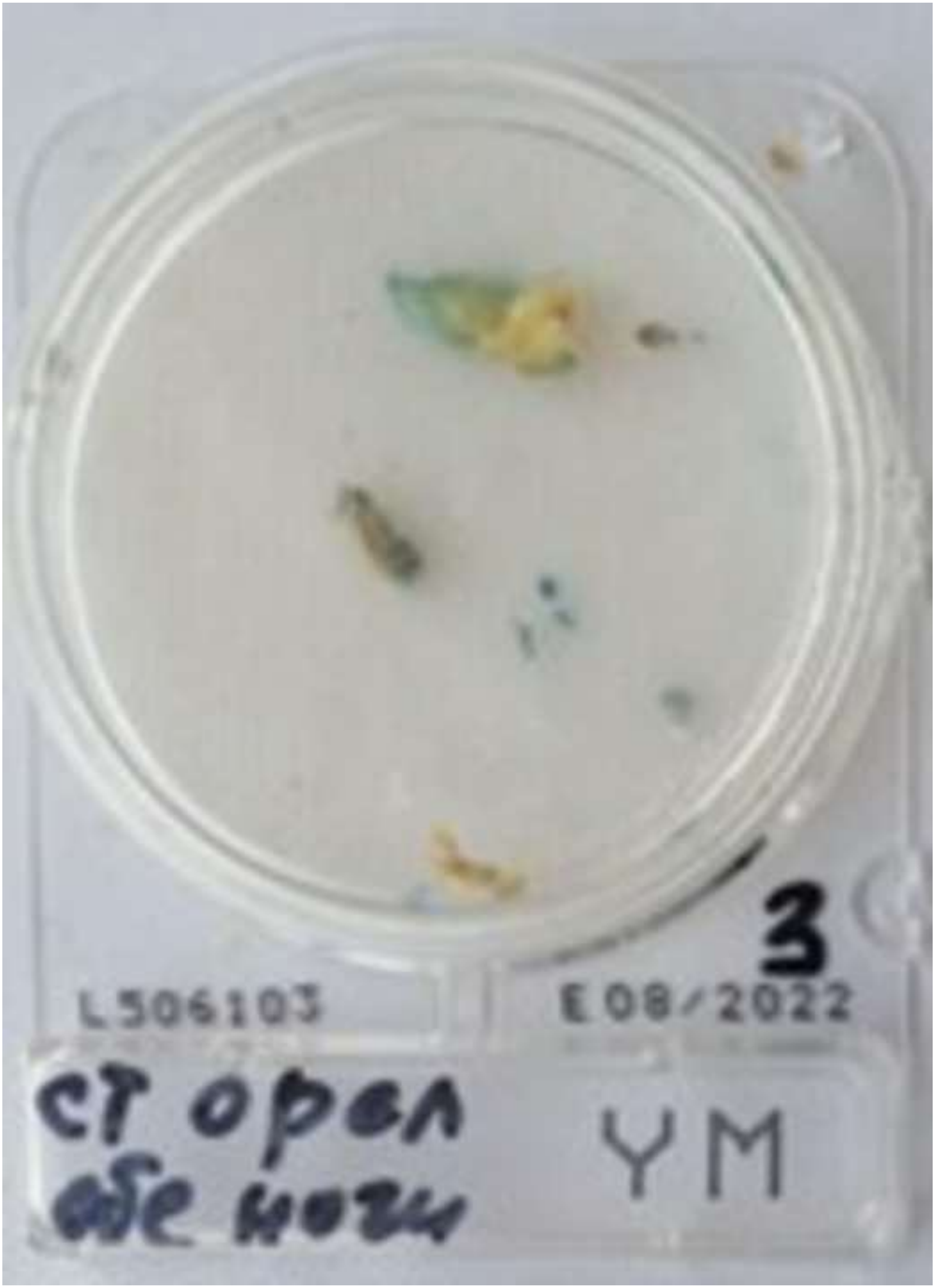

Click here to access/download;Figure;Fig 3 (2).jpg

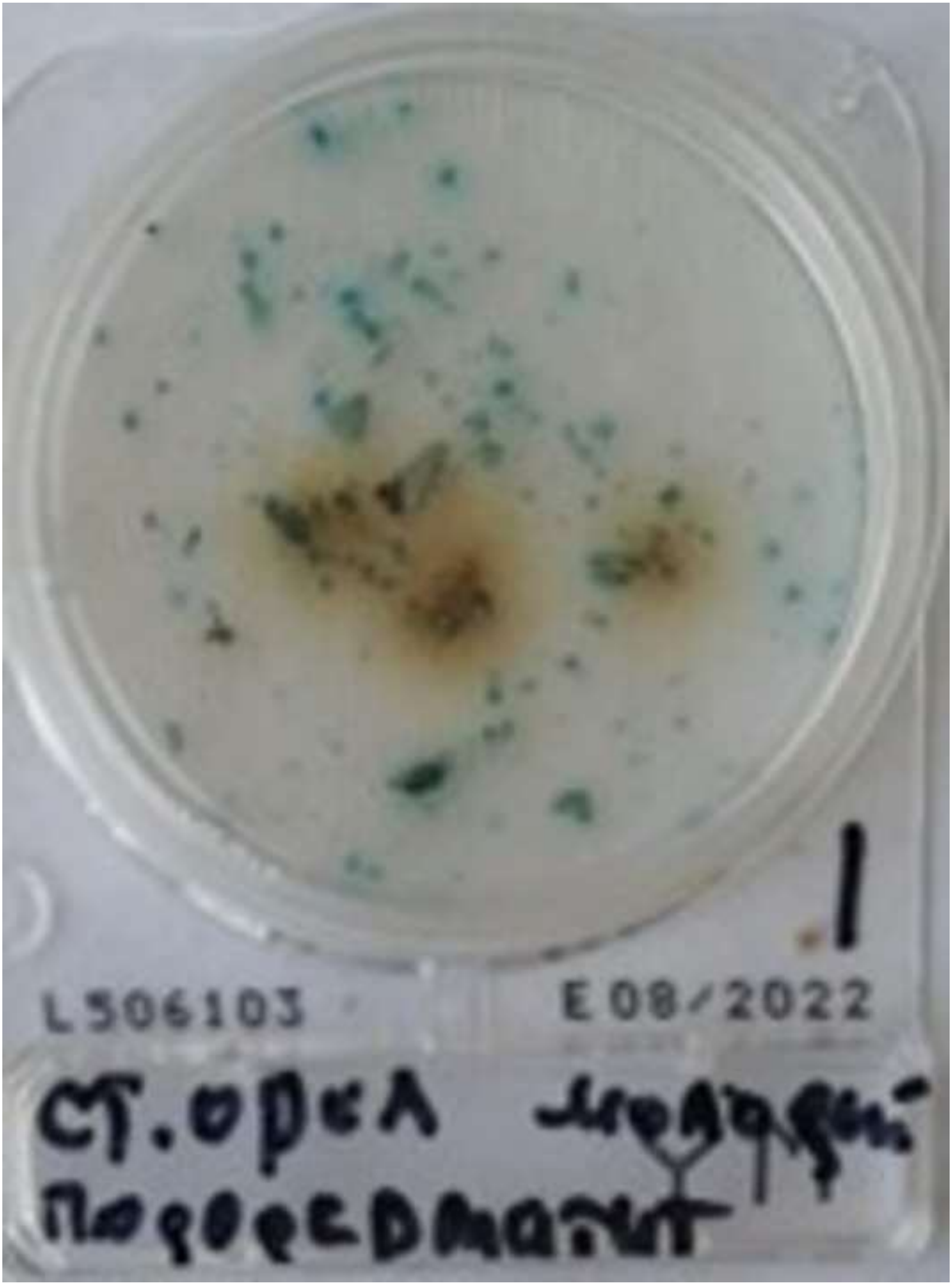

Click here to access/download;Figure;Fig 3 (3).jpg

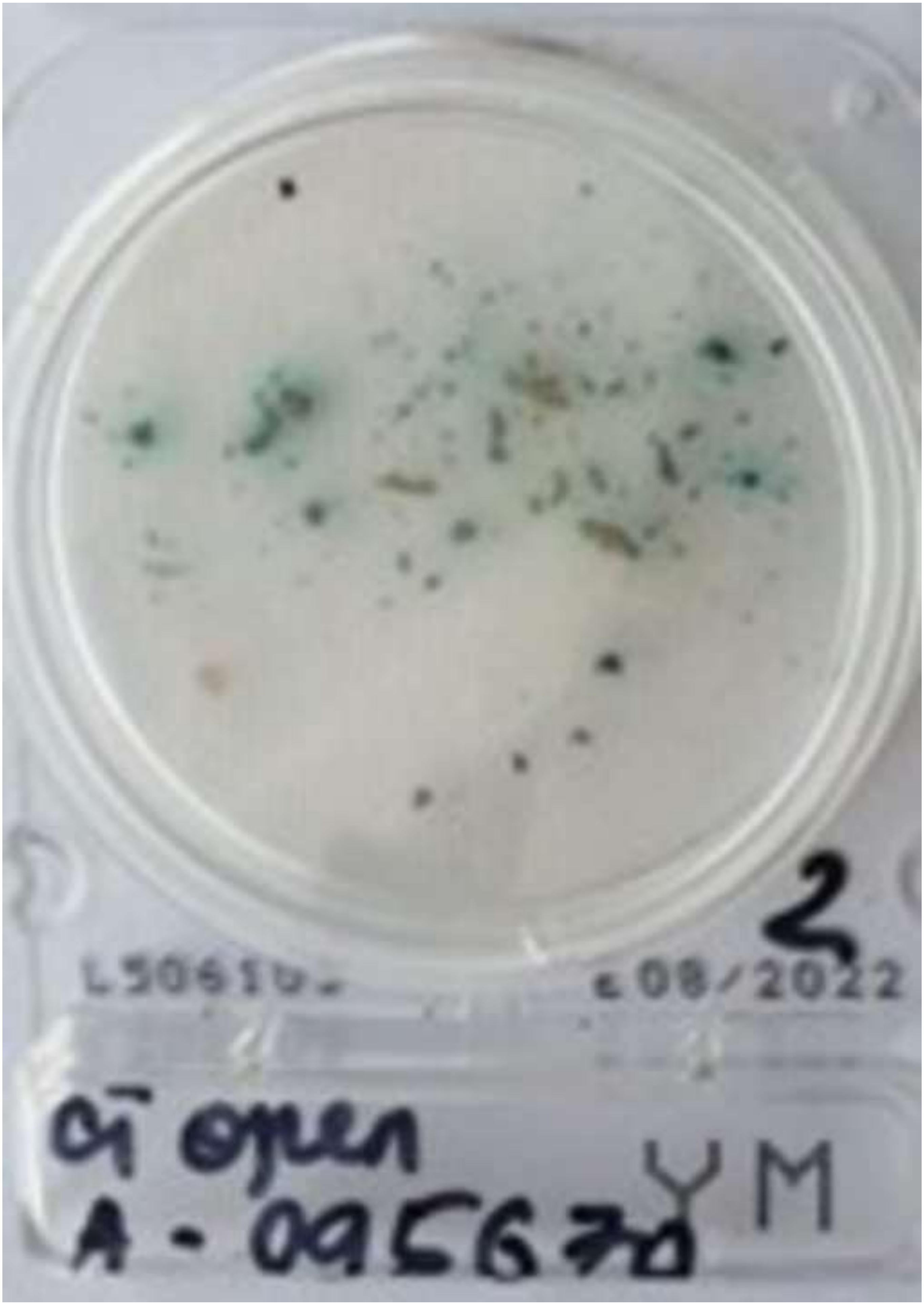

Click here to access/download;Figure;Fig 4 (1).png

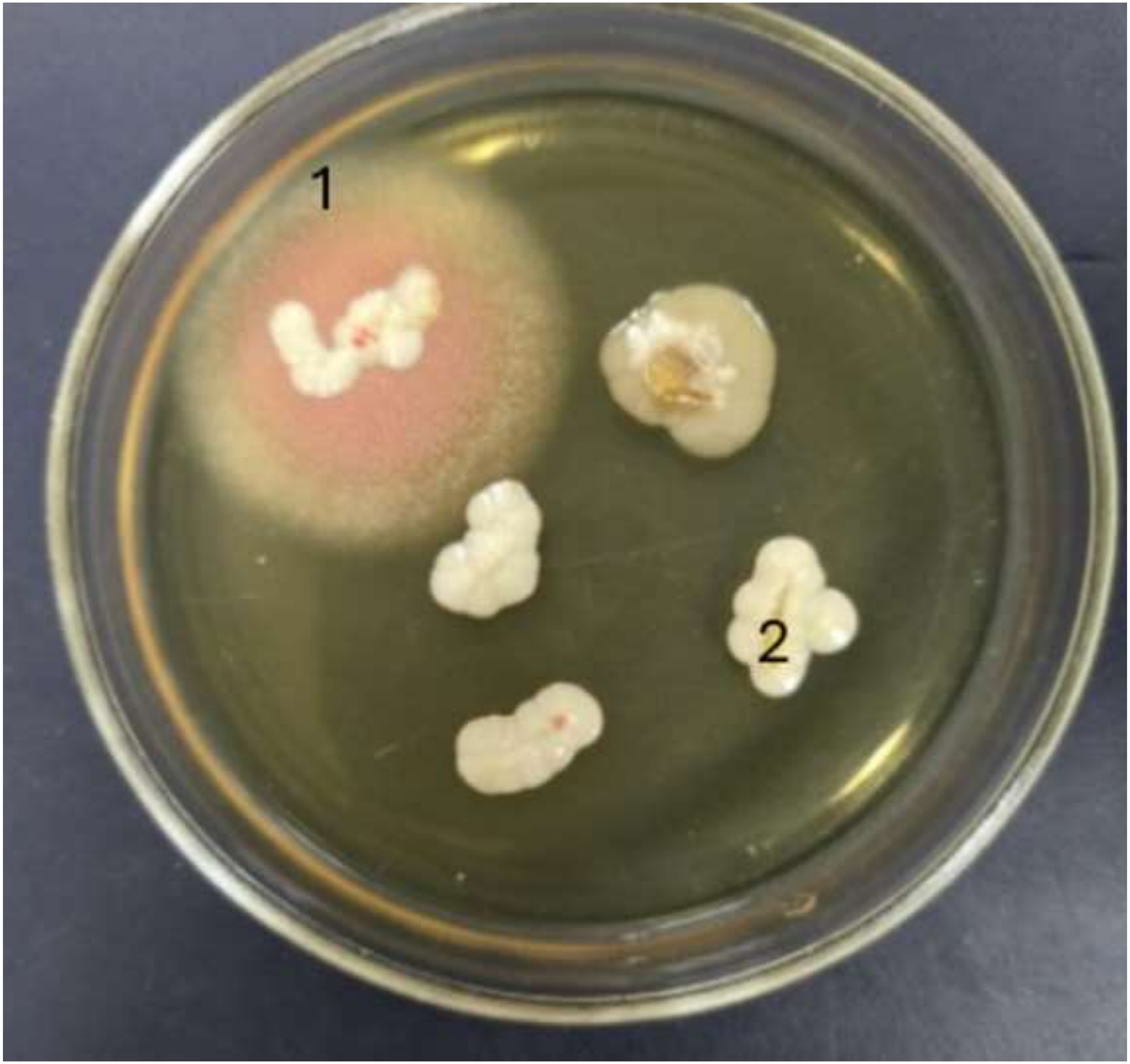

Click here to access/download;Figure;Fig 4 (2).png

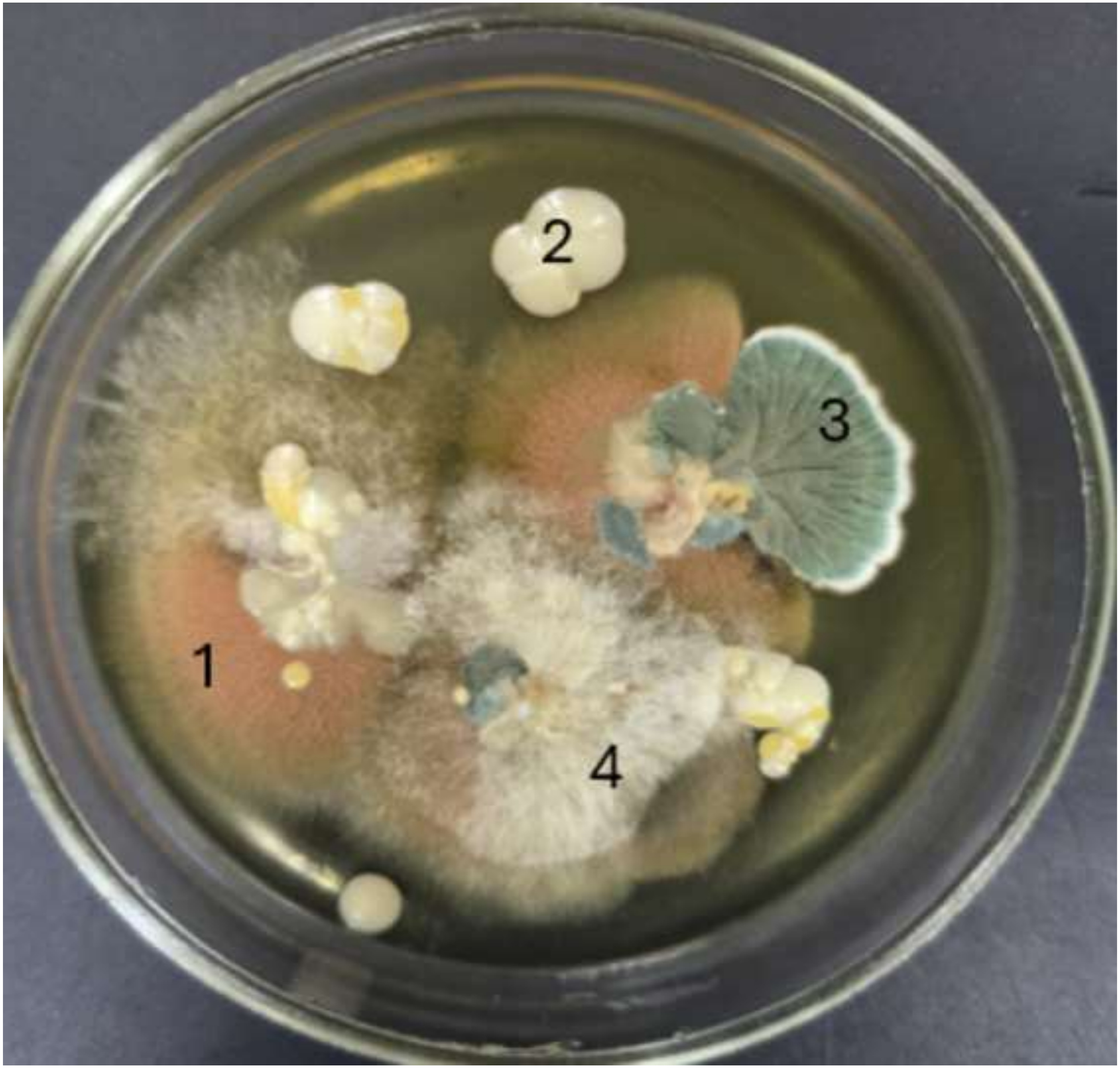

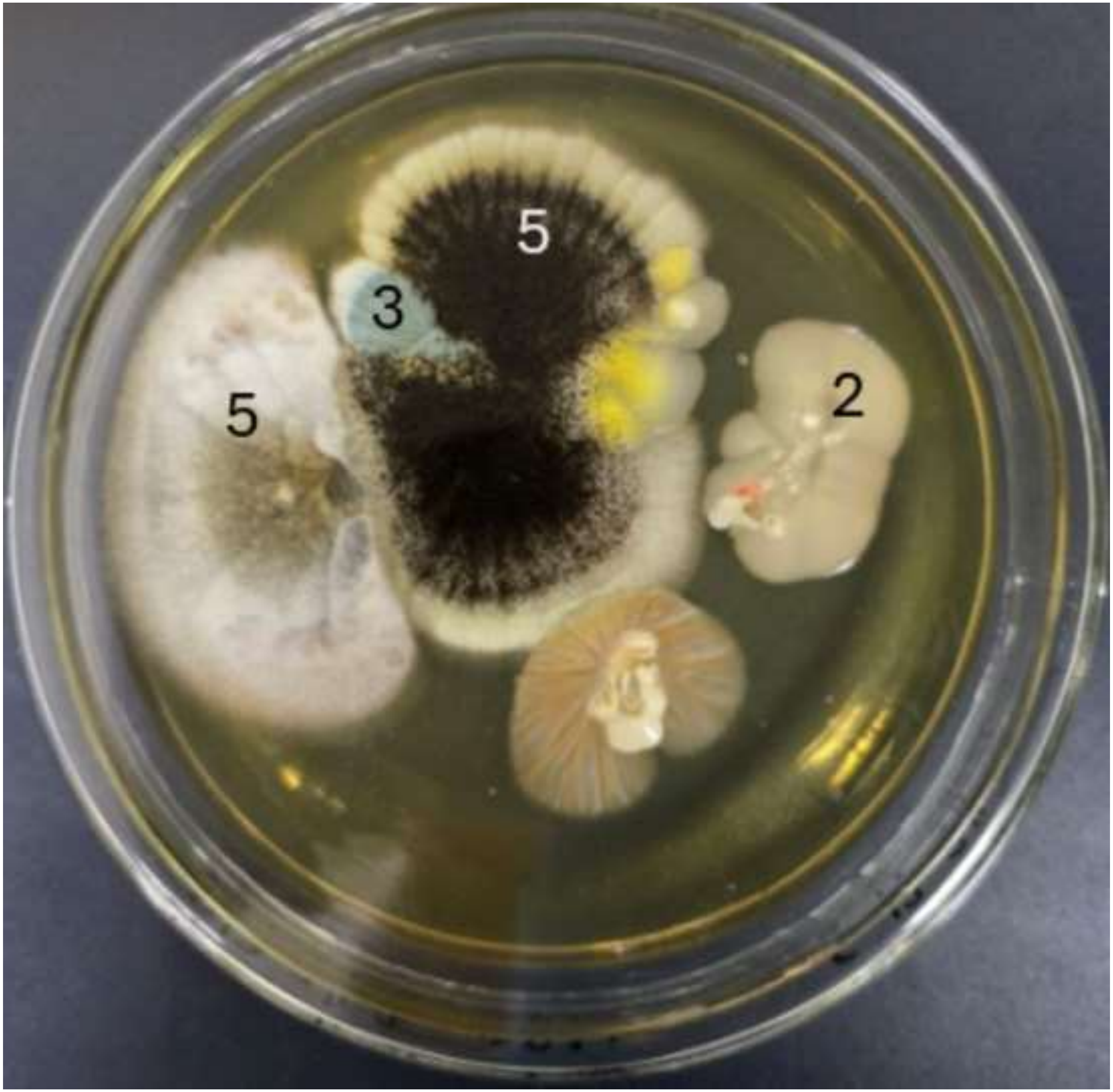
5
3
5
2

Click here to access/download;Figure;Fig 5 17.24.1 eagle.png

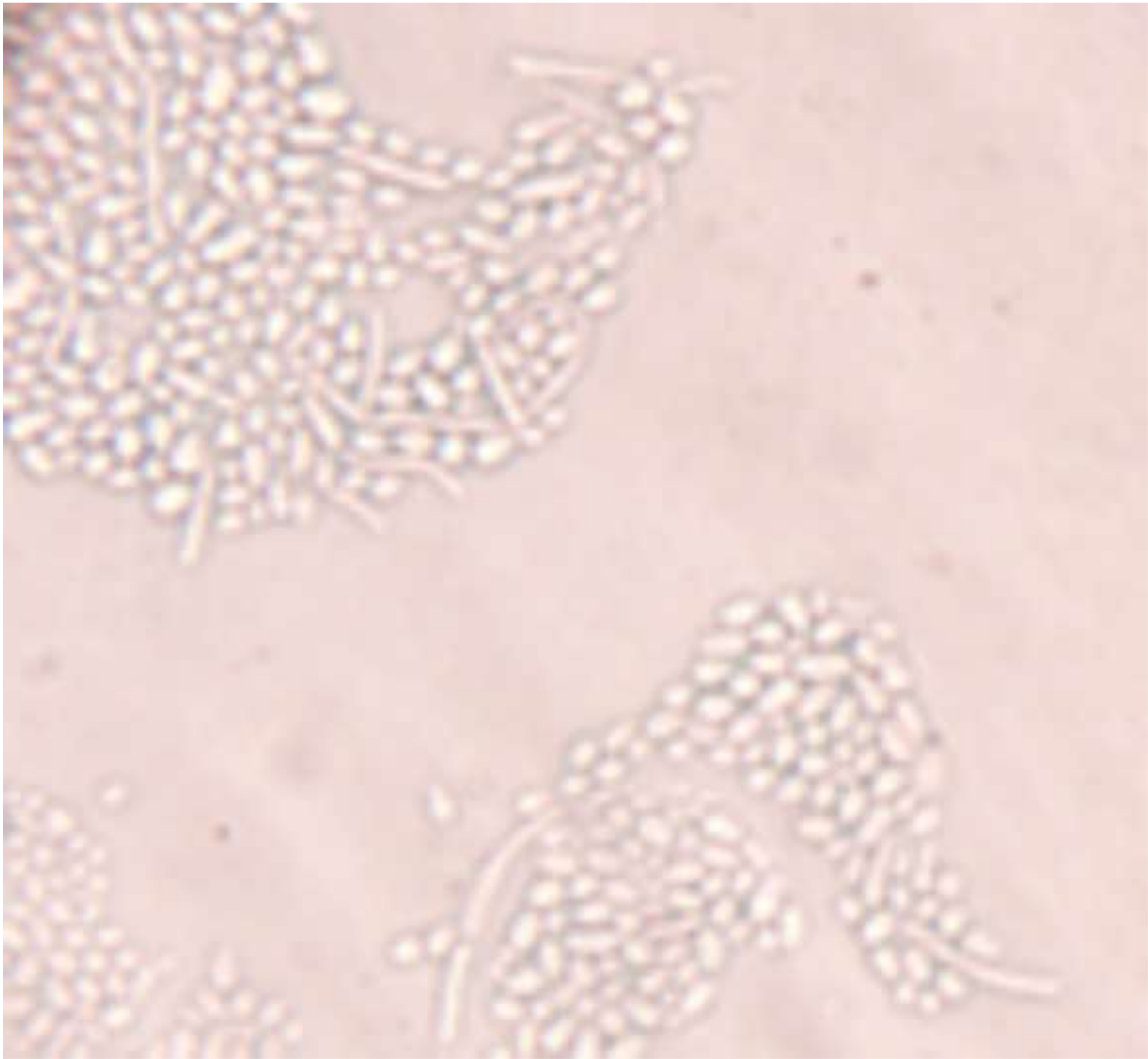

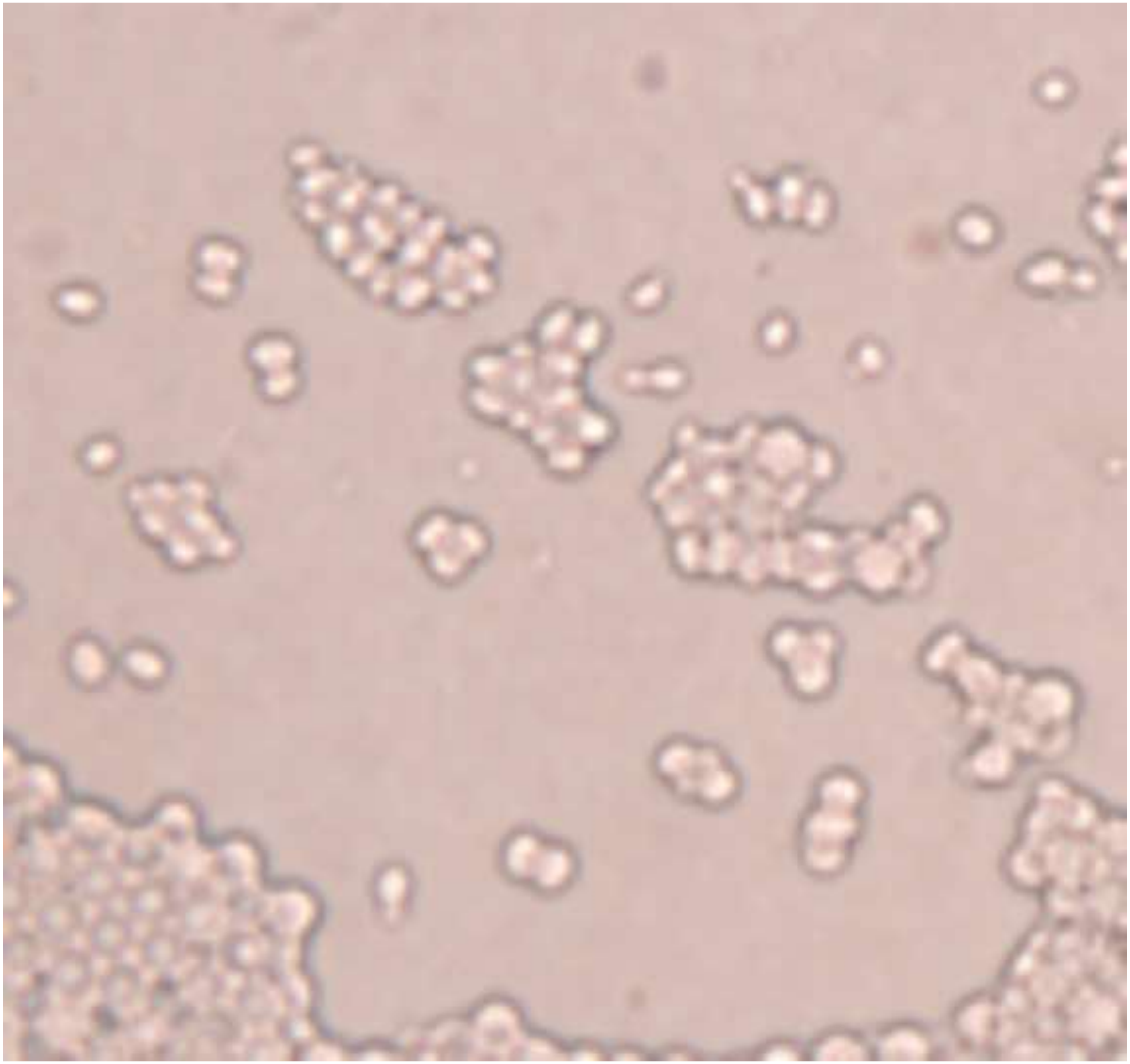

Click here to access/download;Figure;Fig 5 17.24.3 eagle.png

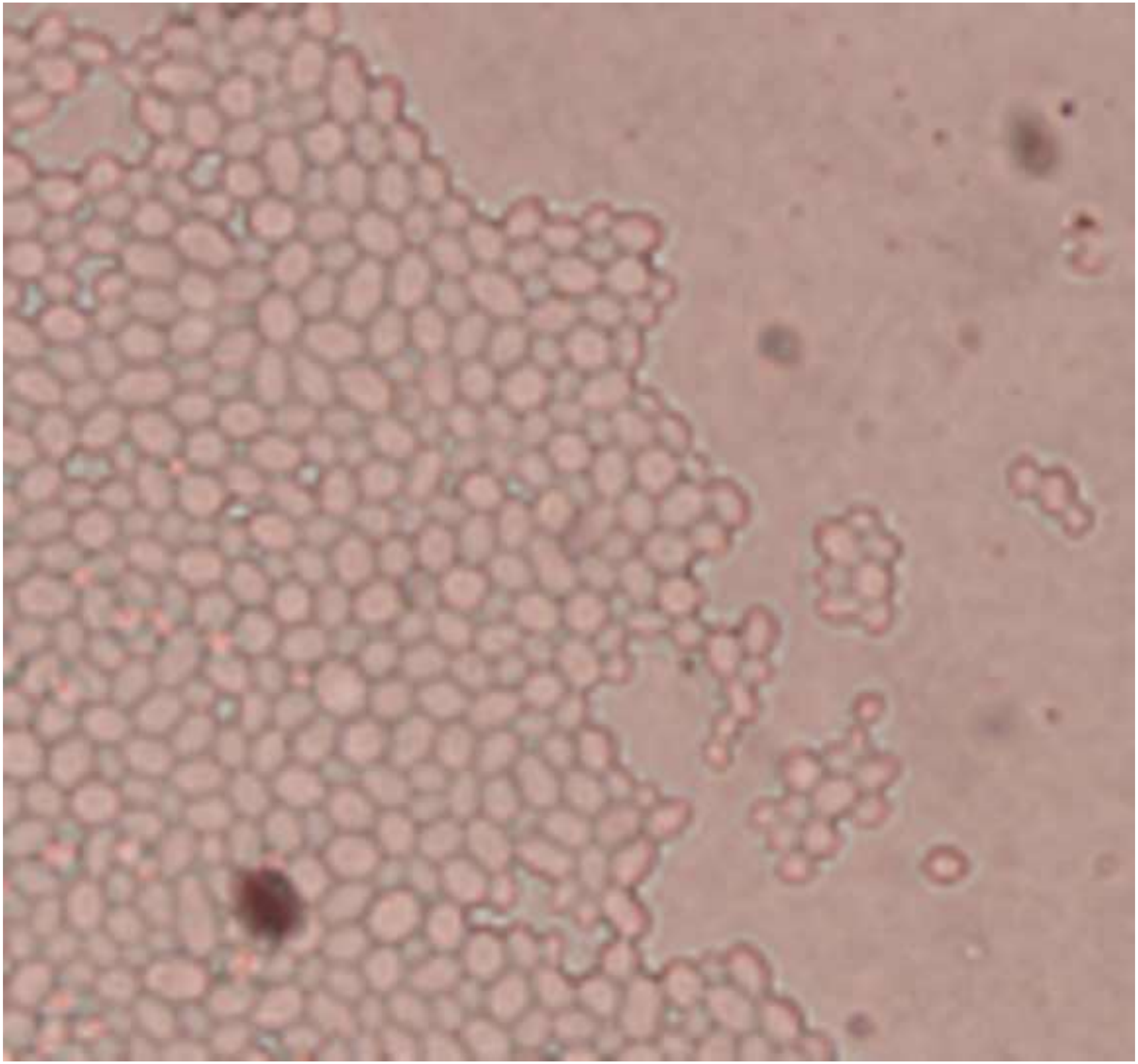

Click here to access/download;Figure;renamed_c33f9.jpg

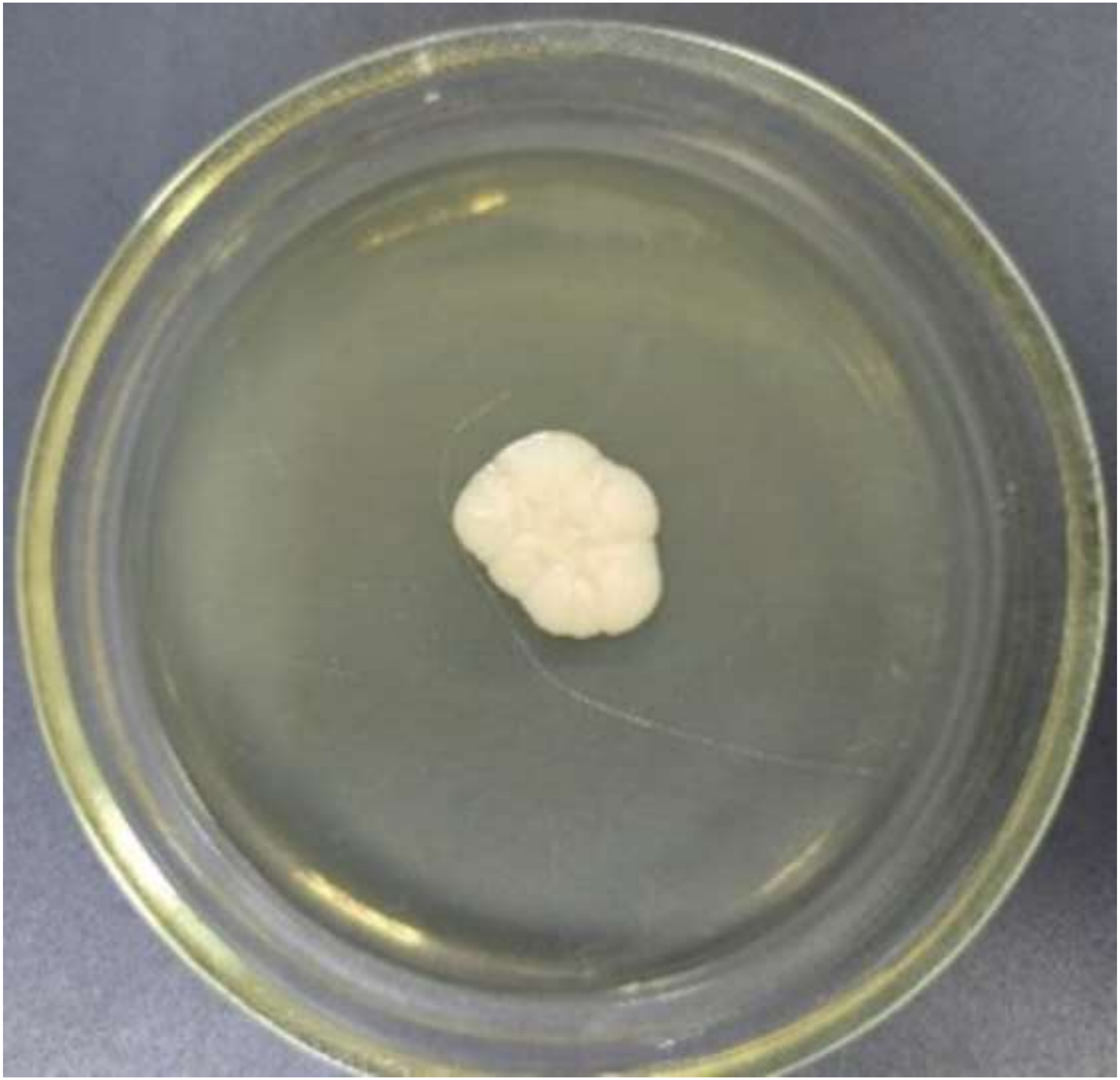

Click here to access/download;Figure;renamed_cf327.jpg

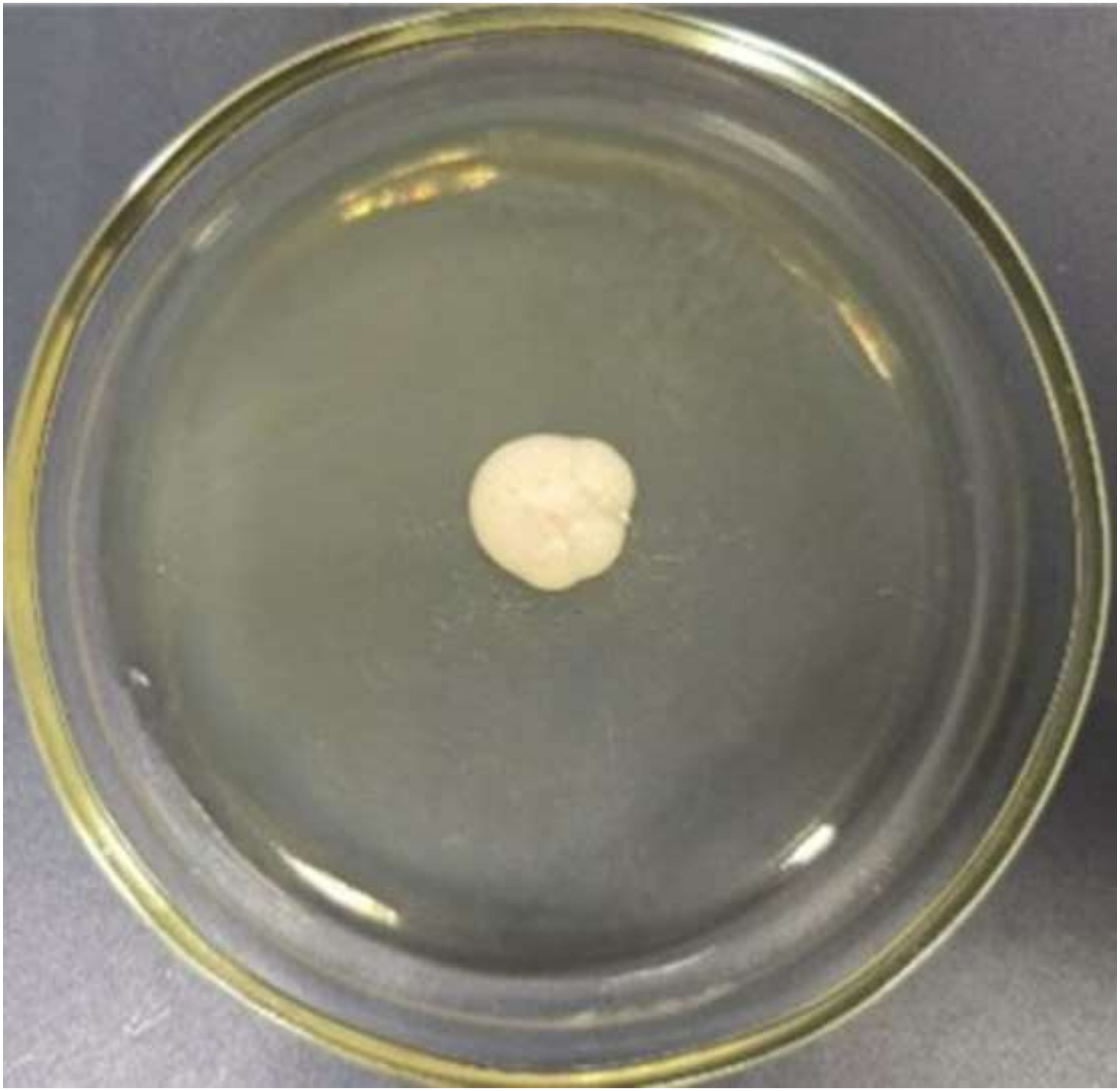

Click here to access/download;Figure;renamed_5afe3.jpg

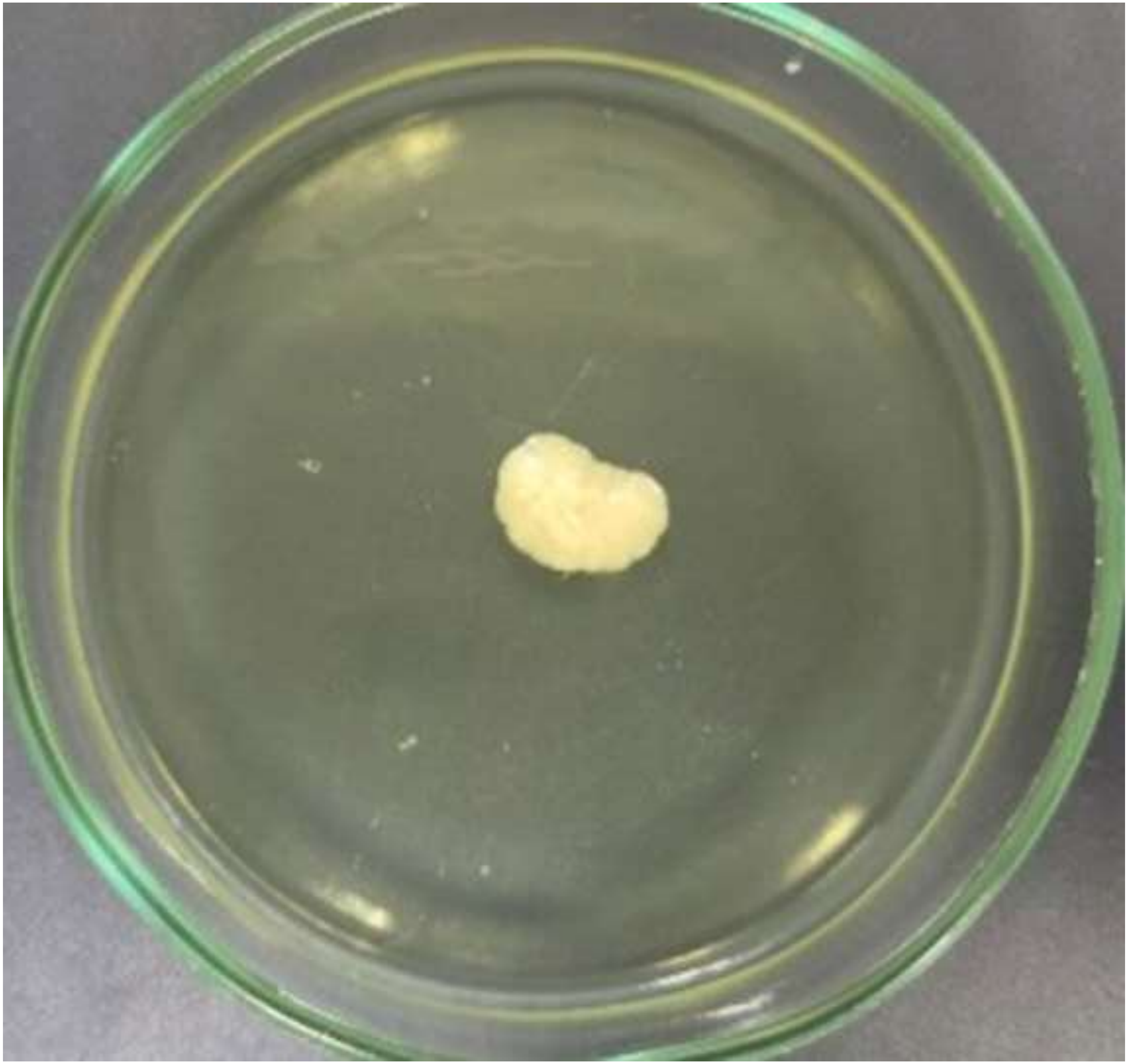

Click here to access/download;Figure;Fig 7.jpg

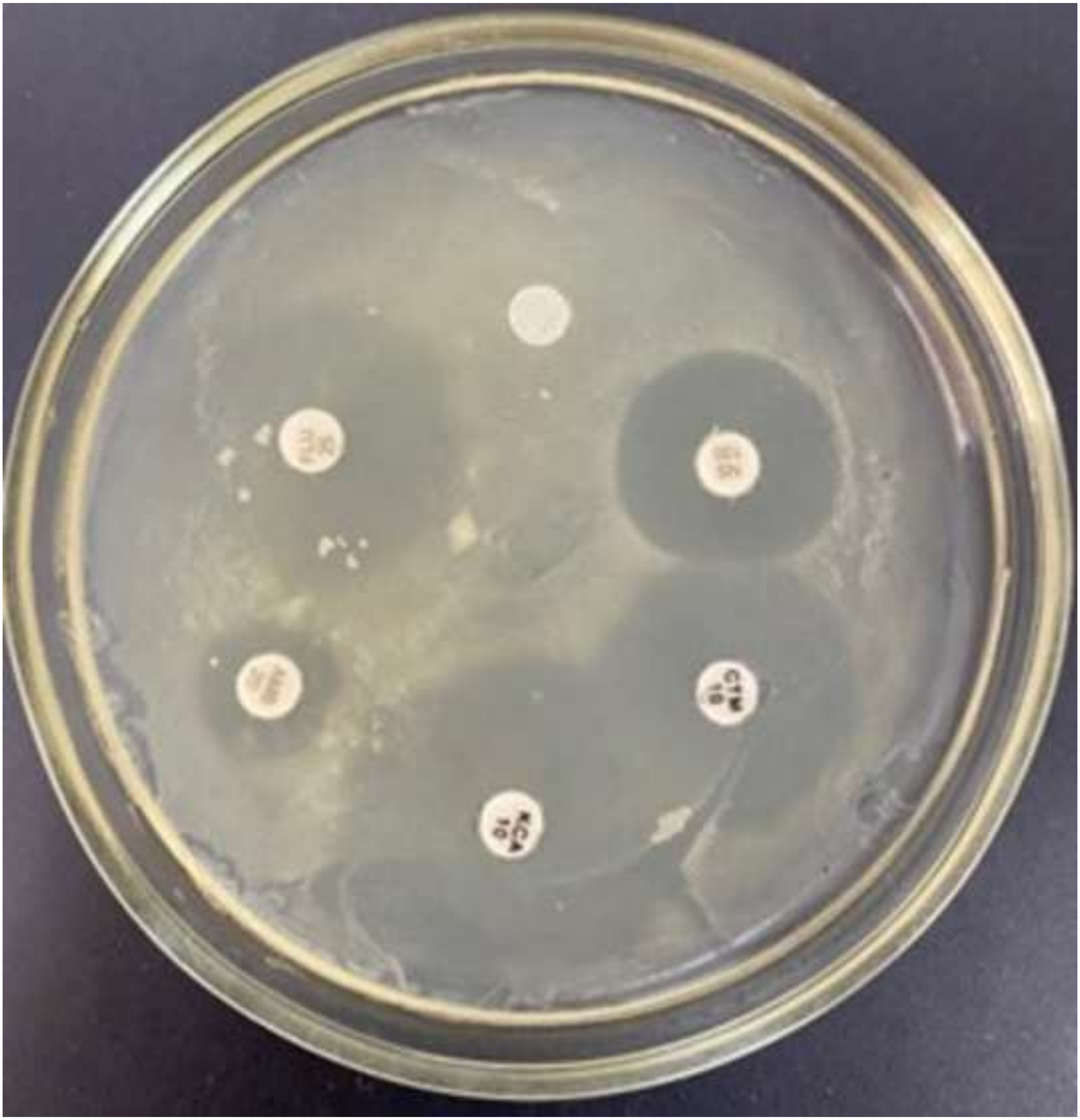

Click here to access/download;Figure;Fig 8 (1).jpg

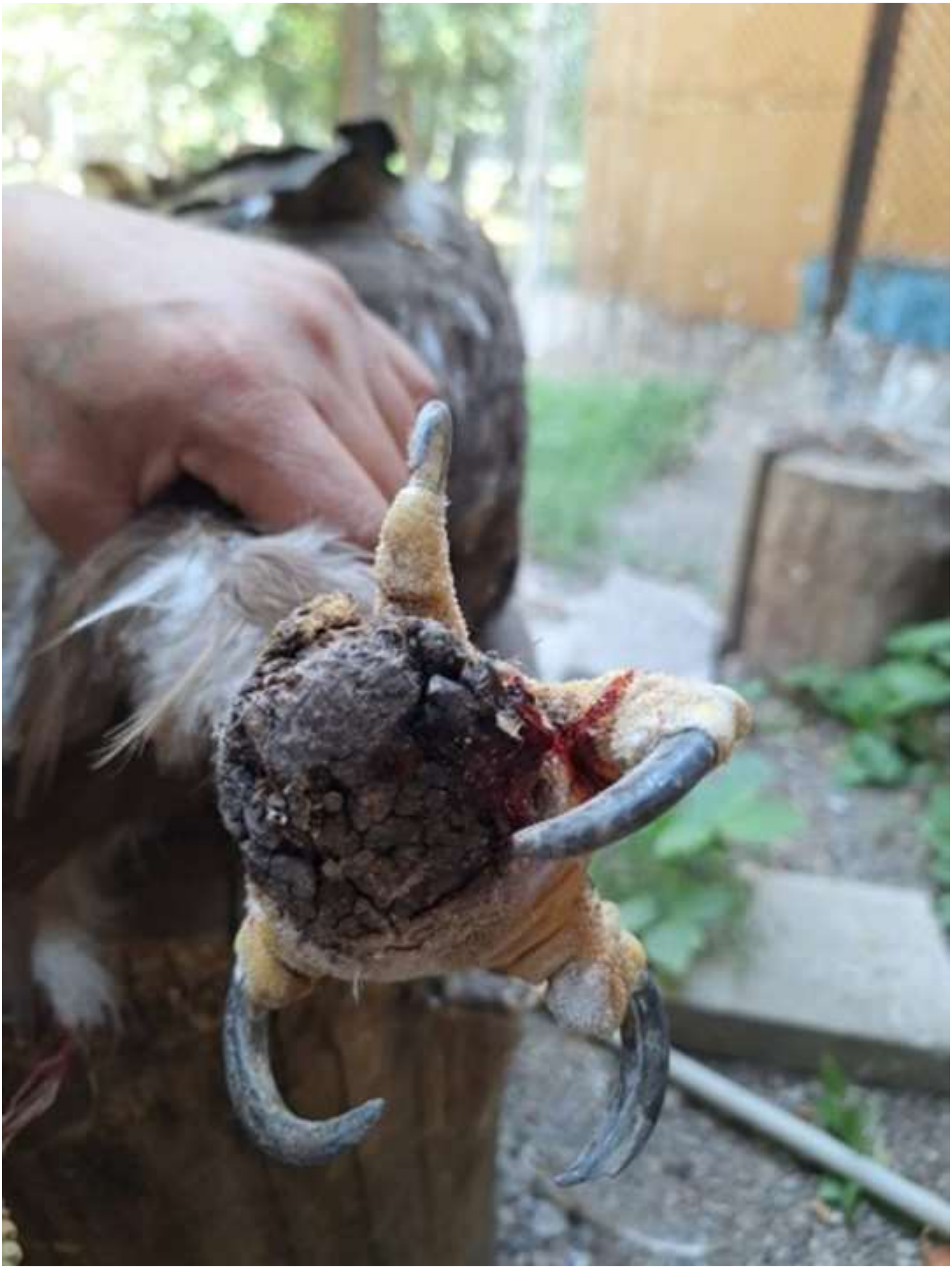

Click here to access/download;Figure;Fig 8 (2).jpg

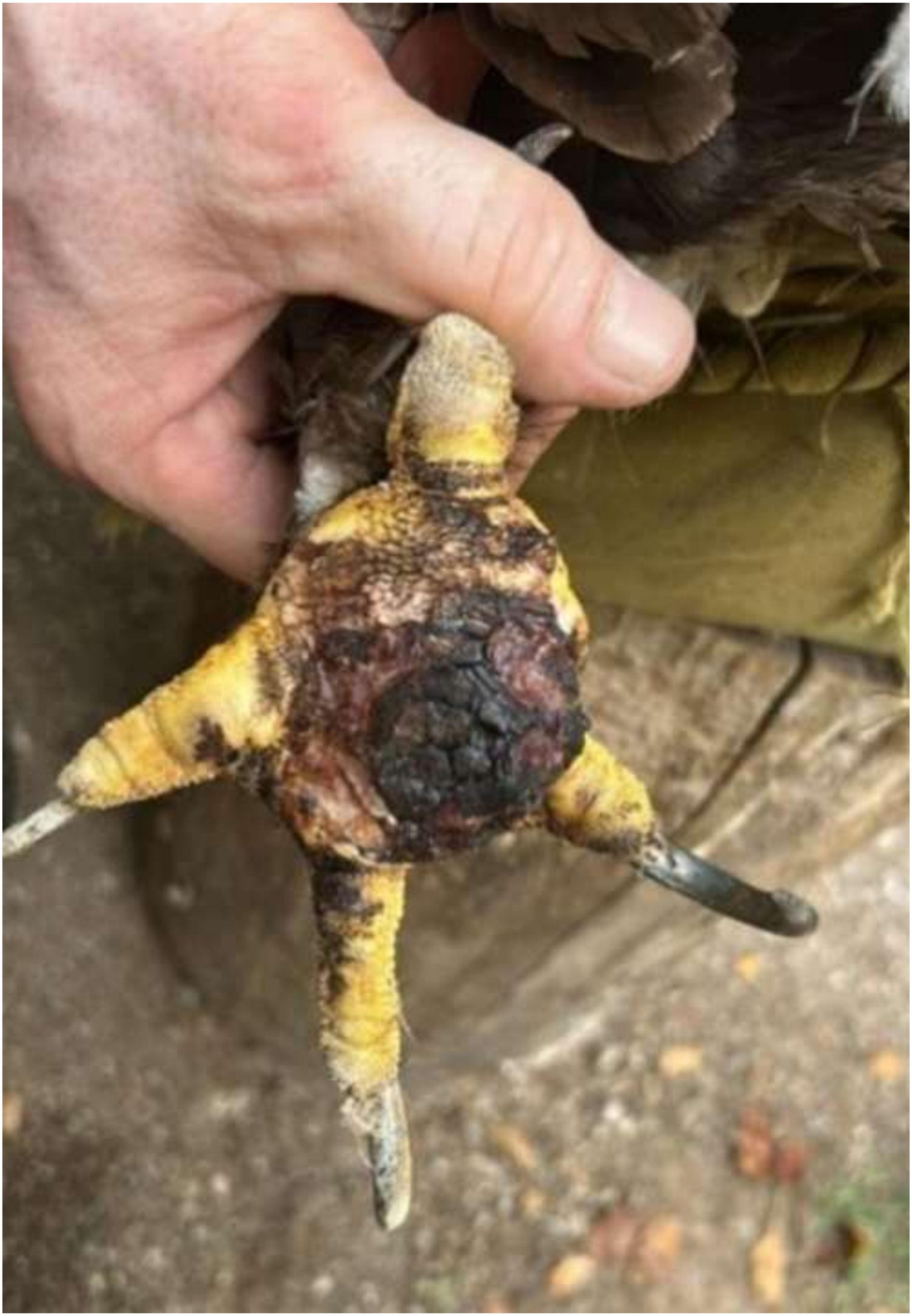

Click here to access/download;Figure;Fig 8 (3).jpg

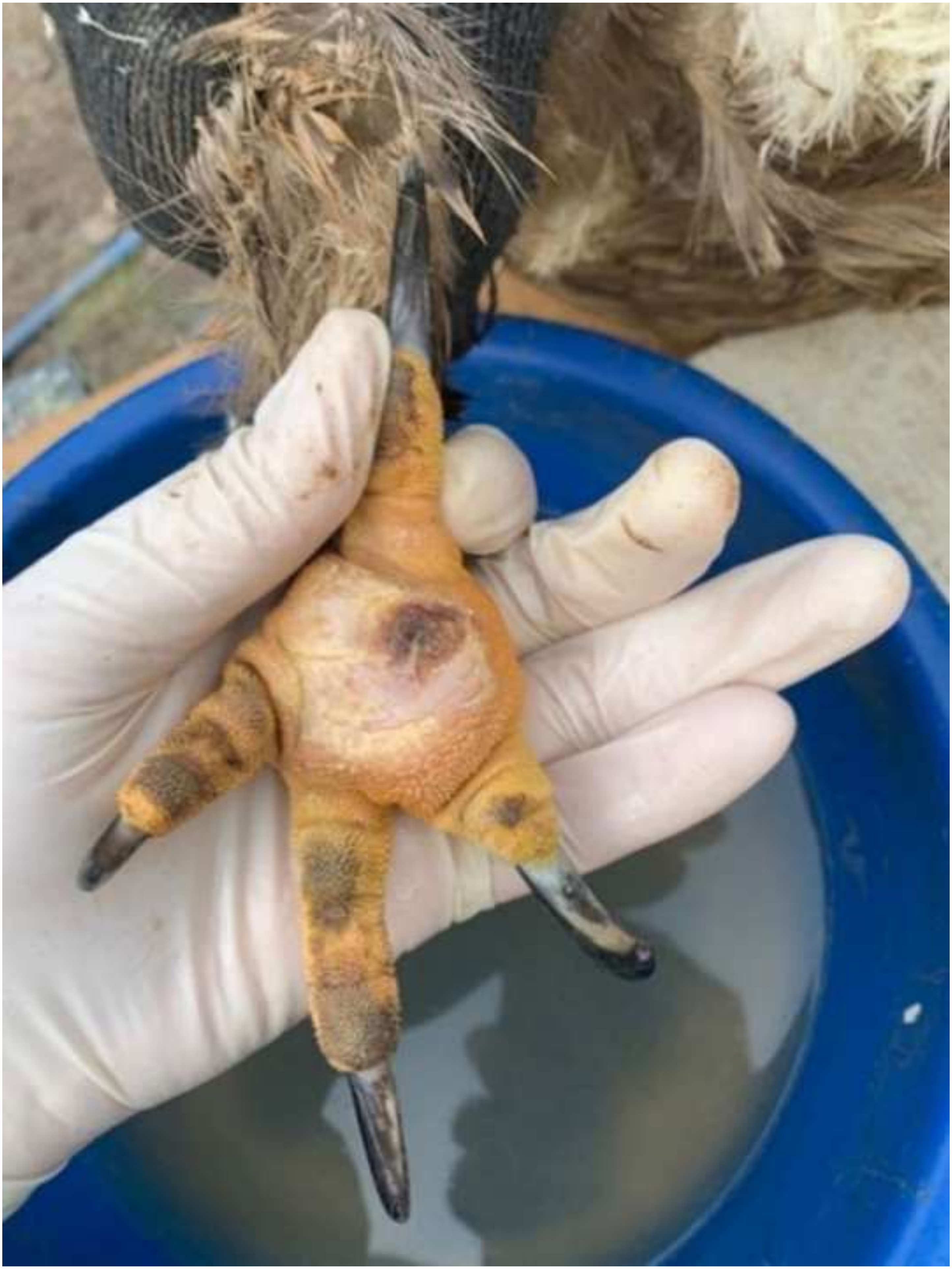

Supplement: Attachment — Submitted filename: PONE-D-26-07402_reviewer.pdf [file pone.0353552.s001.pdf]
