# Supplementary material for: Isolation and characterization of Candida metapsilosis from foci of chronic pododermatitis in captive steppe eagles in Kazakhstan
Source: PLoS One. 2026 Jul 31;21(7):e0353552. doi: 10.1371/journal.pone.0353552 (PMC13426984; doi:10.1371/journal.pone.0353552)
Supplement: Attachment — Submitted filename: Response_to_Reviewers.docx [file pone.0353552.s003.docx]

**Response to Reviewers**

**Manuscript title:** Isolation and characterization of *Candida metapsilosis* from foci of chronic pododermatitis in captive steppe eagles in Kazakhstan

**Corresponding author:** Nessipbayeva A.E.

Dear Editor and Reviewers,

We sincerely thank the editor and all four reviewers for the thorough and constructive evaluation of our manuscript. The comments have substantially helped us to improve the clarity, methodological rigour and scientific value of the paper. We have addressed every point raised, and the changes are highlighted in the revised manuscript using the track-changes feature.

In particular, we would like to highlight the following major revisions made in response to the reviewers' comments:

- All three ITS sequences were re-amplified and re-sequenced bidirectionally, and re-deposited in GenBank with the corrected lengths (PV945930.1, 416 bp; PV945931.1, 413 bp; PV981955.2, 467 bp).
- A **phylogenetic tree** (Maximum-Likelihood, Tamura–Nei model, MEGA 12.1) was constructed to demonstrate the position of our isolates within the *C. metapsilosis* clade (new Fig 6).
- The Materials and Methods section has been extensively revised: the biochemical methodology, the hair-perforation test scoring system, the antifungal susceptibility testing protocol (CLSI M44-A2 with explicit limitations), and bidirectional ITS sequencing are now described in detail. The Ethics Statement has been expanded to comply with the journal's requirements.
- The Discussion has been substantially restructured to (i) explicitly acknowledge *C. metapsilosis* as an opportunistic pathogen exploiting a compromised host, (ii) discuss the role of other isolated environmental moulds, (iii) acknowledge the absence of histopathology as a major limitation, and (iv) tone down speculative interpretations.
- A Data Availability Statement has been added, and the Conclusion section has been rewritten as a concise summary of the key findings.

All page and line numbers in our responses below refer to the revised manuscript with tracked changes accepted. Please find our point-by-point responses below.

Sincerely,

The authors

# Response to Reviewer #1

We thank the reviewer for the positive assessment of our work and for the clear identification of the major issues that needed to be addressed before potential acceptance. We have carefully revised the manuscript according to all of the reviewer's comments.

**Comment 1.1 - Methodology of biochemical analyses (API20 Candida / ID 32 C)**

***Reviewer #1:*** *The MM for the biochemical analyses is lacking. We don't know how you performed them. Household approaches? API 20 Candida? ID 32 C?*

**Response:** We agree that the original description was insufficient. We have clarified in the Materials and Methods section (subsection on biochemical characterization) that we used **conventional in-house biochemical assays on individual classical media** (Giss media for sugar fermentation, Christensen urea agar, meat-peptone gelatin, skim milk, peptone water, blood and keratin-enriched Sabouraud agar) **rather than commercial identification panels such as API 20C AUX or ID 32 C (bioMérieux), which were not available in our laboratory**. We explicitly state that definitive species-level identification therefore relied on ITS rDNA sequencing, while the biochemical assays were used to characterize the metabolic and pathogenicity-related phenotypes of the isolates. (Materials and Methods, *Biochemical characterization* subsection.)

**Comment 1.2 - Short ITS sequences and absence of forward/reverse sequencing**

***Reviewer #1:*** *Some of your ITS sequences are very short, too short for a successful sequencing. A correct ITS sequencing is supposed to give between 700/750 to 850/900 bp. Not around 250. Furthermore you do not explain if you have only a forward, reverse or both sequencing.*

**Response:** We thank the reviewer for identifying this critical issue. We acknowledge that the originally submitted short sequences were inadequate for species-level identification. We have therefore **repeated the entire molecular workflow**: DNA was re-extracted, the ITS1–5.8S–ITS2 region was re-amplified, and the amplicons were **sequenced bidirectionally using both the ITS1 (forward) and ITS4 (reverse) primers**; consensus sequences were assembled from both reads. The new sequences have been re-deposited in GenBank under the following accession numbers: **PV945930.1 (416 bp), PV945931.1 (413 bp) and PV981955.2 (467 bp)**. The corresponding electrophoresis image illustrating the proper amplicon length is included in the manuscript. The Materials and Methods section now explicitly describes the bidirectional sequencing strategy and the consensus assembly step. (Materials and Methods, *Molecular identification* subsection; Results; Discussion.)

**Comment 1.3 - BLAST identity and species identification**

***Reviewer #1:*** *PV981955 has a length of 239 bp, an E-value which is not 0.0 and an identity of 95.44%. Below 98 (or 99% if we want to be conservative) it is unlikely to be the correct species blasted. The same can be said for PV945931.1 (96.13% identity).*

**Response:** We fully agree with the reviewer. Following the re-sequencing described in our response to comment 1.2, the new ITS sequences now meet conservative criteria for species-level identification within the *C. parapsilosis* complex. The updated BLAST results show: **PV945930.1 (416 bp, bit score 769, E = 0.0)**, **PV945931.1 (413 bp, bit score 763, E = 0.0)**, and **PV981955.2 (467 bp, bit score 863, E = 0.0)**. These values are reported in the revised Results and Discussion sections, replacing the previous problematic data. Identification was further confirmed by phylogenetic analysis, in which all three isolates clustered within the *C. metapsilosis* clade together with reference sequences (see new Fig 6 and response to Reviewer #3).

**Comment 1.4 - Figure 7 / CLSI antifungal susceptibility testing**

***Reviewer #1:*** *Figure 7: this is not a CLSI result. Either you use antifungal discs (not a golden standard method) or microdilution method (CLSI – golden laboratory standard). I'd advise to present CLSI results.*

**Response:** We thank the reviewer for this important methodological point. We have clarified in the Materials and Methods that we used **disk diffusion in accordance with CLSI document M44-A2** (Mueller–Hinton agar supplemented with 2% glucose and 0.5 μg/mL methylene blue, inoculum adjusted to a 0.5 McFarland standard, incubation at 35 ± 2 °C for 24 h). We have also **explicitly acknowledged the limitations of M44 in our setting**: validated zone-diameter breakpoints are currently provided only for fluconazole and voriconazole against a limited number of *Candida* species, and no species-specific clinical breakpoints are available for *C. metapsilosis* or for nystatin, clotrimazole, ketoconazole and amphotericin B. Categorical thresholds previously applied to disk-diffusion testing of yeasts in veterinary studies were therefore used (≥20 mm = S, 15–19 mm = I/SDD, <15 mm = R). We now explicitly state that the present results constitute a **screening characterization** rather than a formal clinical susceptibility categorization, and that **broth microdilution according to CLSI M27 (reference standard for yeasts)** will be performed in a follow-up study to determine MIC values. The non-standard terminology ("weakly sensitive") has been replaced with the standard CLSI categories throughout the manuscript.

# Response to Reviewer #2

We thank the reviewer for the very detailed and constructive comments, particularly regarding the methodology, the BLAST analysis and the framing of the Discussion. We have addressed every point and we believe the manuscript is substantially stronger as a result.

**Comment 2.1 - Vague biochemical methodology; hair perforation test quantification**

***Reviewer #2:*** *For the keratinophilic activity (hair perforation test), which specific protocol was used? Was it in vitro? How was it quantified to be deemed "high"?*

**Response:** We have substantially expanded the description of the hair perforation test. The method is based on the protocol of Ajello (1967) and was performed *in vitro* on autoclaved hair fragments incubated with yeast suspensions on yeast-extract-supplemented water agar. For each isolate, **at least 30 hair fragments were examined microscopically at ×400 magnification on days 7, 14, 21 and 30 of incubation**, and keratinolytic activity was scored semi-quantitatively as follows: **negative** (no perforations), **low** (perforations on ≤25% of fragments, single perforations per hair), **moderate** (26–50%), and **high** (>50%, with multiple perforation sites per hair). All assays were performed in duplicate, and isolates were classified as "high" only when both replicates met this criterion. (Materials and Methods, *Keratinolytic activity* subsection.)

**Comment 2.2 - CLSI standards (M27 vs M38 vs M44); breakpoints terminology**

***Reviewer #2:*** *The specific CLSI standards (e.g., M27 for yeasts, not M38) must be correctly cited. The authors cite M38-A2 and M44-A2. M44 is for disk diffusion, but the results are presented in a way that suggests a dilution method. Which method was actually used, and how were the breakpoints determined? "Weakly sensitive" is not standard CLSI terminology for yeasts.*

**Response:** We thank the reviewer for highlighting this confusion. The reference to M38-A2 (which is for filamentous fungi) was an error and has been **removed**. The method actually used was **disk diffusion in accordance with CLSI M44-A2** (full protocol detailed in the response to Reviewer #1, comment 1.4). We have also replaced the non-standard "weakly sensitive" terminology with the standard CLSI categorical interpretation (S / I / SDD / R). We explicitly note in the manuscript that species-specific clinical breakpoints are not available for *C. metapsilosis* or for several of the antifungals tested, and that the present results should therefore be considered a screening characterization. Broth microdilution according to CLSI M27 will be performed in a follow-up study.

**Comment 2.3 - BLAST comparison to C. parapsilosis instead of C. metapsilosis reference strains**

***Reviewer #2:*** *Why compare to a C. parapsilosis sequence if you have identified it as C. metapsilosis? A direct comparison to confirmed C. metapsilosis reference strains (e.g., ATCC 96144) or other GenBank accessions would be more appropriate. The varying query coverage (750 bp vs 669 bp vs 442 bp) is not explained.*

**Response:** We fully agree with the reviewer and we apologize for the confusion. We have **revised the BLAST comparison to use C. metapsilosis reference sequences only**, with the type-strain-derived sequence **PV416737.1 (isolated from cattle, Italy)** used as the primary reference. Additional *C. metapsilosis* reference sequences from animal sources (MF797776.1, MZ540270.1), environmental sources (JQ665419.1, FJ515169.1, FJ515199.1, FJ515200.1, QR731586.1) and human clinical sources (MH715400.1, PQ626156.1, MK561031.1, KU200445.1, KJ816926.1, KJ816917.1, KJ816927.1) were also compared. The *C. parapsilosis* sequence (KC777375.1) has been removed from this comparison. The varying query coverage in the previous version is no longer relevant, because after re-sequencing all three isolates yielded ITS amplicons of comparable, appropriate length (416, 413 and 467 bp), and the consensus sequences are based on bidirectional reads with full coverage.

**Comment 2.4 - Overstated discussion of "uniqueness"**

***Reviewer #2:*** *Phrases like "the uniqueness of C. metapsilosis isolation… explains the difficulties in diagnosis" are speculative.*

**Response:** We agree. The corresponding paragraph has been **substantially rewritten** to remove speculative wording. The current text reads (Discussion): *"The first reported case of C. metapsilosis isolation from wild eagles of the Kazakh population kept in long-term captivity is consistent with the clinical picture observed in this case: the attending veterinarians initially treated the lesions as bacterial pododermatitis and did not prescribe antifungal therapy, which may have contributed to the chronic course of the disease. … we therefore suggest, rather than conclude, that broader use of mycological work-up in non-resolving cases could be useful."* The original speculative claim has been removed.

**Comment 2.5 - Opportunistic pathogen vs. primary cause**

***Reviewer #2:*** *Were these eagles sick because of C. metapsilosis, or was C. metapsilosis present because the eagles were sick and immunosuppressed? The wet bedding, poor nutrition, and stress are classic predisposing factors. The discussion must acknowledge that the fungus is likely an opportunistic pathogen exploiting a compromised host and environment, rather than the primary cause.*

**Response:** This is a very important point and we fully agree. The Discussion has been **substantially restructured** to explicitly frame *C. metapsilosis* as an opportunistic pathogen. The relevant text now reads: *"…damp litter, poor nutrition, and stress are classic factors predisposing to opportunistic infections. Given that the fungus C. metapsilosis is an opportunistic pathogen that exploits vulnerabilities in the host and the environment, it was the cause of the yeast mycosis, which developed as a secondary infection when favorable conditions arose…"* In a new Discussion paragraph we also state that the keratinolytic activity, thermotolerance and (in one strain) pseudohyphal formation of these isolates are *"consistent with an opportunistic pathogen that can play a role in the chronic progression of pododermatitis in immunocompromised birds kept under poor husbandry conditions, rather than with a primary, obligate pathogen"*. The terminology "causative agent" has been replaced throughout with "contributory aetiological role" or "opportunistic fungal contributor".

**Comment 2.6 - Pseudohyphae attributed to genetic differences without WGS**

***Reviewer #2:*** *The authors attribute pseudohyphae formation to "genetic differences," but without whole-genome sequencing, this is pure speculation.*

**Response:** We agree and we have substantially softened this discussion. The relevant text now explicitly acknowledges the absence of whole-genome sequencing: *"This may be related to strain-specific differences among the analyzed isolates; however, in the absence of whole-genome sequencing we cannot rigorously establish a genetic basis for this phenotype, and we therefore present this observation as a working hypothesis rather than a firm conclusion. Although all isolates were subcultured under nominally identical conditions… we cannot fully exclude an effect of the in vitro micro-environment at the time of subculture…"* We have also added that **whole-genome sequencing and comparative transcriptomics** of the three isolates are planned in our future work to clarify this point.

**Comment 2.7 - Absence of histopathology**

***Reviewer #2:*** *No histopathology was performed in this study, which is a major limitation that should be mentioned.*

**Response:** We fully agree and we have addressed this in two places. First, in the dedicated Limitations section, where *"Absence of histological pathology"* is explicitly listed. Second, in a new dedicated Discussion paragraph, we now state: *"A major limitation of the present work, which we underline here in addition to the limitations listed below, is the absence of histopathology of the affected footpad tissue, which would have allowed direct visualization of fungal elements within the lesions and a more rigorous assessment of tissue invasion versus surface colonization; histopathological correlation should therefore be a priority in any future study of fungal pododermatitis in raptors."*

# Response to Reviewer #3

We thank the reviewer for the constructive comments and suggestions. The construction of a phylogenetic tree, in particular, has substantially strengthened the species identification, and the additional discussion of other co-isolated fungi has made the case for *C. metapsilosis* as the principal fungal contributor more rigorous.

**Comment 3.1 - Was C. metapsilosis the only causative agent?**

***Reviewer #3:*** *How can you be sure that C. metapsilosis was the only causative agent? You mention that you also isolated other fungi.*

**Response:** This is a very important point. We have added **a dedicated Discussion paragraph** that critically addresses this question. We explain that the other filamentous fungi recovered from the primary cultures (*Fusarium* spp., *Penicillium* spp., *Mucor* spp. and *Aspergillus* spp., Fig 4) are common environmental moulds, and their isolation most likely reflects contamination of the wet, soiled bedding rather than an aetiological role in the foot lesions. Four lines of evidence support this interpretation: (i) these moulds were not recovered consistently from all three birds; (ii) they did not dominate the subcultures from the deep tissue scrapings; (iii) on subculture from the affected footpad tissue, only *Candida*-like colonies were repeatedly and abundantly recovered, with similar morphology in all three eagles; and (iv) only the *Candida* isolates combined the in vitro features most relevant to footpad pathology (thermotolerance up to 37 °C, urease activity and clear keratinolytic activity in the hair-perforation assay). Together with the clinical response to topical antimycotic therapy, these observations support *C. metapsilosis* as *"the most likely fungal contributor to the chronic lesions, although we cannot formally exclude an additional, transient role of other environmental moulds"*. The wording has been deliberately chosen to reflect the appropriate level of certainty.

**Comment 3.2 - Figure 7 does not comply with CLSI guidelines**

***Reviewer #3:*** *You should review the CLSI document; Figure 7 does not comply with the guidelines set forth in the documents.*

**Response:** Thank you for this comment, which echoes the concerns raised by Reviewers #1 and #2. The Materials and Methods section has been revised to **explicitly state the use of the CLSI M44-A2 disk-diffusion protocol**, to use standard CLSI categorical terminology (S / I / SDD / R), and to acknowledge the methodological limitations (absence of species-specific clinical breakpoints for *C. metapsilosis* and for most of the antifungals tested). The figure caption and the corresponding Results paragraph have been updated accordingly. (See response to Reviewer #1, comment 1.4, for the full description.)

**Comment 3.3 - Phylogenetic tree; low identity percentages for two strains**

***Reviewer #3:*** *Is it possible to construct a phylogenetic tree? The percentages for the two strains are too low to be considered the same species (metapsilosis).*

**Response:** We agree that a phylogenetic analysis is the appropriate way to address this question. We have constructed **a Maximum-Likelihood phylogenetic tree (new Fig 6)** based on aligned ITS1–5.8S–ITS2 sequences (309 positions in the final dataset, 12 sequences), using the Tamura–Nei (1993) nucleotide substitution model as implemented in MEGA 12.1. The initial tree for the heuristic search was selected from the better-scoring of a Neighbor-Joining tree and a Maximum-Parsimony tree. The tree with the highest log-likelihood (−1398.60) is shown. The low identity percentages observed in the original BLAST analysis were caused by the short and incomplete ITS sequences originally deposited; after re-sequencing (see response to Reviewer #1, comment 1.2), all three isolates now yield BLAST identities and E-values consistent with *C. metapsilosis*, and on the phylogenetic tree the three isolates cluster closely within the *C. metapsilosis* clade with the reference type-strain-derived sequence PV416737.1.

**Comment 3.4 - Quality of the figures (blurry)**

***Reviewer #3:*** *The figures are of very poor quality. They appear blurry.*

**Response:** We thank the reviewer for this observation. All figures have been **re-prepared at a higher resolution (≥600 dpi for line art and ≥300 dpi for photographs)** and re-inserted into the manuscript. Figure 6 (the new phylogenetic tree) has been exported as a vector graphic from MEGA 12.1 to ensure publication-quality sharpness. Figures showing macroscopic and microscopic findings have been re-acquired at higher magnification where possible, and the figure files in the final formats (TIFF / PDF) will be provided as separate high-resolution files at the next submission step.

# Response to Reviewer #4

We thank the reviewer for the positive overall assessment and for the constructive suggestion regarding the structure of the Conclusion section.

**Comment 4.1 - Conclusion section needs improvement; relocation of speculative content**

***Reviewer #4:*** *The authors can improve the quality of their write-up in the conclusion section by summarizing their key findings, and removing some sections such as lines 428–433 on page 21. This section could be part of the discussion in the manuscript.*

**Response:** We have followed the reviewer's recommendation closely. The paragraph in lines 428–433 of the previous version, which *speculated on the pathogenic potential of C. metapsilosis*, **has been removed from the Conclusion** and its core message has been **relocated to the Discussion** within the new paragraph addressing the opportunistic nature of the pathogen (see response to Reviewer #2, comment 2.5). The Conclusion has been **rewritten as a concise summary of the key findings**, including the species identification, the in vitro virulence-associated traits (keratinolytic and urease activity, thermotolerance, saccharolytic and proteolytic profiles), the antifungal susceptibility profile, the clinical outcome of targeted treatment, and the take-home message that mycological work-up should be included in the diagnostic algorithm for non-resolving pododermatitis in captive birds of prey.

We trust that the revised manuscript now adequately addresses all of the reviewers' concerns and that it meets the publication standards of the journal. We thank the editor and the four reviewers once again for the time and expertise invested in evaluating our work.

Sincerely,

The authors
