# Supplementary material for: Isolation and characterization of Candida metapsilosis from foci of chronic pododermatitis in captive steppe eagles in Kazakhstan
Source: PLoS One. 2026 Jul 31;21(7):e0353552. doi: 10.1371/journal.pone.0353552 (PMC13426984; doi:10.1371/journal.pone.0353552)
Supplement: Peer review history

# Peer review: Decision Letter 0

**DOI:** 10.1371/journal.pone.0353552.r001

**Authors:** Petr Heneberg

19 Apr 2026

PONE-D-26-07402

The etiological role of the yeast-like fungus Candida metapsilosis in the development of chronic pododermatitis in captive steppe eagles

PLOS One

Dear Dr. Nessipbayeva,

Thank you for submitting your manuscript to PLOS ONE. After careful consideration, we feel that it has merit but does not fully meet PLOS ONE’s publication criteria as it currently stands. Therefore, we invite you to submit a revised version of the manuscript that addresses the points raised during the review process.

Please submit your revised manuscript by Jun 03 2026 11:59PM. If you will need more time than this to complete your revisions, please reply to this message or contact the journal office at plosone@plos.org. When you're ready to submit your revision, log on to https://www.editorialmanager.com/pone/ and select the 'Submissions Needing Revision' folder to locate your manuscript file.

Please include the following items when submitting your revised manuscript:

- A letter that responds to each point raised by the academic editor and reviewer(s). You should upload this letter as a separate file labeled 'Response to Reviewers'.
- A marked-up copy of your manuscript that highlights changes made to the original version. You should upload this as a separate file labeled 'Revised Manuscript with Track Changes'.
- An unmarked version of your revised paper without tracked changes. You should upload this as a separate file labeled 'Manuscript'.

If you would like to make changes to your financial disclosure, please include your updated statement in your cover letter. Guidelines for resubmitting your figure files are available below the reviewer comments at the end of this letter.

If applicable, we recommend that you deposit your laboratory protocols in protocols.io to enhance the reproducibility of your results. Protocols.io assigns your protocol its own identifier (DOI) so that it can be cited independently in the future. For instructions see: https://journals.plos.org/plosone/s/submission-guidelines#loc-laboratory-protocols. Additionally, PLOS ONE offers an option for publishing peer-reviewed Lab Protocol articles, which describe protocols hosted on protocols.io. Read more information on sharing protocols at https://plos.org/protocols?utm_medium=editorial-email&utm_source=authorletters&utm_campaign=protocols.

As the corresponding author, your ORCID iD is verified in the submission system and will appear in the published article. PLOS supports the use of ORCID, and we encourage all coauthors to register for an ORCID iD and use it as well. Please encourage your coauthors to verify their ORCID iD within the submission system before final acceptance, as unverified ORCID iDs will not appear in the published article. *Only*  the individual author can complete the verification step; PLOS staff *cannot*  verify ORCID iDs on behalf of authors.

We look forward to receiving your revised manuscript.

Kind regards,

Petr Heneberg

Academic Editor

PLOS One

Journal Requirements:

hen submitting your revision, we need you to address these additional requirements.

1. Please ensure that your manuscript meets PLOS ONE's style requirements, including those for file naming. The PLOS ONE style templates can be found at

https://journals.plos.org/plosone/s/file?id=wjVg/PLOSOne_formatting_sample_main_body.pdf and

https://journals.plos.org/plosone/s/file?id=ba62/PLOSOne_formatting_sample_title_authors_affiliations.pdf.

2. Thank you for stating the following financial disclosure:

“The study was funded by grant AP23489001 (2024-2026) from the Ministry of Education and Science of the Republic of Kazakhstan. “

Please state what role the funders took in the study.  If the funders had no role, please state: "The funders had no role in study design, data collection and analysis, decision to publish, or preparation of the manuscript."

If this statement is not correct you must amend it as needed.

Please include this amended Role of Funder statement in your cover letter; we will change the online submission form on your behalf.

3. When completing the data availability statement of the submission form, you indicated that you will make your data available on acceptance. We strongly recommend all authors decide on a data sharing plan before acceptance, as the process can be lengthy and hold up publication timelines. Please note that, though access restrictions are acceptable now, your entire data will need to be made freely accessible if your manuscript is accepted for publication. This policy applies to all data except where public deposition would breach compliance with the protocol approved by your research ethics board. If you are unable to adhere to our open data policy, please kindly revise your statement to explain your reasoning and we will seek the editor's input on an exemption. Please be assured that, once you have provided your new statement, the assessment of your exemption will not hold up the peer review process.

4. Thank you for stating the following in the Acknowledgments Section of your manuscript:

“The study was funded by grant AP23489001 (2024-2026) from the Ministry of Education and Science of the Republic of Kazakhstan”

We note that you have provided additional information within the Acknowledgements Section that is not currently declared in your Funding Statement. Please note that funding information should not appear in the Acknowledgments section or other areas of your manuscript. We will only publish funding information present in the Funding Statement section of the online submission form.

Please remove any funding-related text from the manuscript and let us know how you would like to update your Funding Statement. Currently, your Funding Statement reads as follows:

“The study was funded by grant AP23489001 (2024-2026) from the Ministry of Education and Science of the Republic of Kazakhstan. “

Please include your amended statements within your cover letter; we will change the online submission form on your behalf.

5. Please include your full ethics statement in the ‘Methods’ section of your manuscript file. In your statement, please include the full name of the IRB or ethics committee who approved or waived your study, as well as whether or not you obtained informed written or verbal consent. If consent was waived for your study, please include this information in your statement as well.

6. If the reviewer comments include a recommendation to cite specific previously published works, please review and evaluate these publications to determine whether they are relevant and should be cited. There is no requirement to cite these works unless the editor has indicated otherwise.

[Note: HTML markup is below. Please do not edit.]

Reviewers' comments:

Reviewer's Responses to Questions

**Comments to the Author**

1. Is the manuscript technically sound, and do the data support the conclusions?

The manuscript must describe a technically sound piece of scientific research with data that supports the conclusions. Experiments must have been conducted rigorously, with appropriate controls, replication, and sample sizes. The conclusions must be drawn appropriately based on the data presented.

Reviewer #1: Partly

Reviewer #2: Yes

Reviewer #3: Partly

Reviewer #4: Yes

**********

2. Has the statistical analysis been performed appropriately and rigorously?

Reviewer #1: N/A

Reviewer #2: N/A

Reviewer #3: N/A

Reviewer #4: N/A

**********

3. Have the authors made all data underlying the findings in their manuscript fully available?

The PLOS Data policy requires authors to make all data underlying the findings described in their manuscript fully available without restriction, with rare exception (please refer to the Data Availability Statement in the manuscript PDF file). The data should be provided as part of the manuscript or its supporting information, or deposited to a public repository. For example, in addition to summary statistics, the data points behind means, medians and variance measures should be available. If there are restrictions on publicly sharing data—e.g. participant privacy or use of data from a third party—those must be specified.

Reviewer #1: Yes

Reviewer #2: No

Reviewer #3: Yes

Reviewer #4: Yes

**********

4. Is the manuscript presented in an intelligible fashion and written in standard English?

PLOS ONE does not copyedit accepted manuscripts, so the language in submitted articles must be clear, correct, and unambiguous. Any typographical or grammatical errors should be corrected at revision, so please note any specific errors here.

Reviewer #1: Yes

Reviewer #2: Yes

Reviewer #3: Yes

Reviewer #4: Yes

**********

5. Review Comments to the Author

Please use the space provided to explain your answers to the questions above. You may also include additional comments for the author, including concerns about dual publication, research ethics, or publication ethics. (Please upload your review as an attachment if it exceeds 20,000 characters)

Reviewer #1: Dear Author

Thank you for submitting this work interesting both from medical mycology and veterinary point of view

I have a few minors points that need correction

I have however a few major points that need to be adressed before article potential acceptation

Minor points

Be careful to write all Genera and species in italic (see L70 Staphyloccocus aureus and check all the manuscript)

Be more straightforward in your title : yeast-like fungis C. metapsilosis = yeast C. metapsilosis

L96 : would have been informative if you were more precise on what antimicrobials were used. Here we can only guess about antibiotics but which ones ?

L139 : Levomekol is a commercial name. Be more precise (the main component is chloramphenicol which also adresses my point above)

L162 : seeding at 28°C. OK this is acceptable. But pathogenic yeasts can also grow at higher temperature. Why haven't you tried 30 or 35°C . This would have strenghtened your discussion about thermotolerance -line 303-

some pictures especially from macro and micro cultures are blurry.

Now the major issues

The MM for the biochemical analyses is lacking. We dont' know how your performed them. Household approaches ? , API20 Candida ? ID32 C ?

The main issue is your conclusion based on BLAST results

1- some of your ITS sequences are very short, too short for a successful sequencing. A correct ITS sequening is supposed to give your between 700/750 to 850/900 bp. Not around 250. Futhermore you do not explain if you have only a forward, reverse or both sequencing. Usually, in front of unknown colonies, a forward AND reverse ITS is wanted.

2- this has a direct impact on your species identification

While your PV945930.1 has a lenght of 750 pb, a 100% coverage E0.0 and 99.28% of identity (meaning that it is a C.metapsilosis) the same cannot be said with your other sequences

PV981955. has a lenght of 239 pb, a E which is not 0.0 and an identity of 95.44%. Below 98 (or 99% if we want to be conservative) it is unlikely to be the correct specie blasted. Correct genera, ok, but the specie identification is highly questionable. Which impacts your whole discussion

The same can be said for the PV945931.1 sequence. The lenght and results are a bit better but you are in a grey area (96.13% of identity)

3-Figure 7 : this is not a CLSI result. Either you use antifungal discs (not a golden standard method) or microdilution method (CLSI-golden laboratory standard). I'd advise to present CLSI results.

There are quite a few conceptual sentences that are misleading. Probably lost in translation

L41 Our results indicate the emergence of new pathogenic yeast strains : well, as it is written, C.metapsilosis is new. Which is not the case. refer to sentence L31 which is way more clearer

same for sentence here : Candida metapsilosis was first isolated from wild steppe eagles of the

Kazakh population living in captivity, diagnosed with bacterial pododermatitis,

and from bedding, establishing their etiological role. The sentence is misleading

rework it in a similar way to ; This is the first isolation of C.metapsilosis from wild steppe eagles of the

Kazakh population living in captivity. Their etilogical role in these birds pododermatitis condition was established.

Regards

Reviewer #2: General comments

This manuscript presents a case series of three steppe eagles with chronic pododermatitis from which Candida metapsilosis was isolated. While the novelty of the pathogen is noteworthy, the study's major weakness is its inability to establish causation. It is an observational study with mycological identification, not an experimental infection study that can prove an "etiological role." The title and conclusions are overreaching. The manuscript requires major revisions to reframe the narrative as a descriptive case report with molecular characterization.

Specific Comments

Title

1) Consider "Isolation and characterization of Candida metapsilosis from chronic pododermatitis lesions in captive steppe eagles in Kazakhstan."

Abstract

1) The phrase "Our results indicate the emergence of new pathogenic yeast strains... responsible for pathology" is an overstatement. The data show an association, not definitive responsibility. The presence of the fungus does not prove it is the primary pathogen; it could be a secondary opportunistic colonizer of a wound caused by poor husbandry.

Keywords

1) Replace "Candida albicans" with "Candida metapsilosis" as the primary keyword.

Introduction

1) The introduction fails to state a clear, testable hypothesis or a specific aim for the study. It simply describes what the authors did.

2) The opening sentence about hunting with golden eagles being a "national idea" is too culturally specific for an international journal like PLOS One and detracts from the scientific focus.

Materials and Methods

1) The description of sample collection ("scraping the skin and cutting away necrotic tissue... after properly sterilizing the affected area with 70% alcohol") is insufficient. How was the site sterilized to differentiate infection from contamination? Was a biopsy taken, or just a surface swab/scraping? The method of sterilizing the surface before sampling is crucial to argue that the isolated yeast was truly from the deep tissue and not a surface contaminant.

2) The manuscript relies heavily on morphological and biochemical traits. However, for cryptic species like C. metapsilosis, which is part of the C. parapsilosis complex, molecular identification is the gold standard. The authors did this, which is good, but the methodology for the biochemical tests is vague. For the keratinophilic activity (hair perforation test), which specific protocol was used? Was it in vitro? How was it quantified to be deemed "high"? For the antifungal sensitivity testing (CLSI method), the specific standards (e.g., M27 for yeasts, not M38, which is for filamentous fungi) must be correctly cited. The authors cite M38-A2 and M44-A2. M44 is for disk diffusion, but the results are presented in a way that suggests a dilution method. Which method was actually used, and how were the breakpoints for "sensitive," "weakly sensitive," etc., determined? This is not standard CLSI terminology for yeasts.

Results

1) The results are presented in a narrative style that mixes observations with interpretation. Figures 3, 4, and 5 show primary cultures and microscopy, but the quality of the images (especially Fig. 5, which appears pixelated in the PDF) is poor and needs improvement.

2) The BLAST results are presented confusingly. The authors mention homology with a C. parapsilosis sequence (KC777375.1) used in a mouse model. Why compare to a C. parapsilosis sequence if you have identified it as C. metapsilosis? A direct comparison to confirmed C. metapsilosis reference strains (e.g., ATCC 96144) or other GenBank accessions would be more appropriate. The varying query coverage (750bp vs 669bp vs 442bp) is not explained.

3) The data in Figure 6 is presented without error bars or replicates. Was this experiment performed in triplicate? The authors state that strains have "high" keratinophilic activity, but the figure shows a bar graph. What does the y-axis represent? Quantification of this assay is missing.

Discussion

1) The discussion continues to overstate the findings. Phrases like "the uniqueness of C. metapsilosis isolation... explains the difficulties in diagnosis" are speculative.

2) The authors fail to adequately address the elephant in the room: were these eagles sick because of C. metapsilosis, or was C. metapsilosis present because the eagles were sick and immunosuppressed due to terrible husbandry? The wet bedding, poor nutrition, and stress are classic predisposing factors for opportunistic infections. The discussion must acknowledge that the fungus is likely an opportunistic pathogen exploiting a compromised host and environment, rather than the primary cause.

3) The discussion regarding the pseudohyphae formation in one strain is interesting but over-analyzed. The authors attribute it to "genetic differences," but without whole-genome sequencing, this is pure speculation. It could easily be due to the specific in vitro conditions at the time of subculture.

4) The discussion provides a good list of other Candida species found in birds but does not critically compare the pathological findings (e.g., histopathology) of those cases with the current one. No histopathology was performed in this study, which is a major limitation that should be mentioned.

Limitations

1) The authors did not include a dedicated "Limitations" section. This is a serious omission. The following must be acknowledged: (i) Small Sample Size: A case series of three birds from the same aviary. (ii) Lack of Histopathology: Without histologic examination of the tissue, it is impossible to confirm tissue invasion by the fungus and differentiate it from surface colonization or contamination. (iii) No Control Group: There were no healthy birds sampled from the same or different environments to determine if C. metapsilosis is a normal part of the eagle's skin microbiome or environment. (iv) Confounding Factors: The birds had severe husbandry issues and had received prior antibiotic treatment, which are major risk factors for fungal overgrowth. It is impossible to disentangle the role of the fungus from these factors.

Conclusions

1) Rewrite the conclusion to be more measured. State that C. metapsilosis was isolated from the lesions and characterized, and that its pathogenic potential (keratinolytic activity) suggests it may play a role in the chronicity of the disease, especially in immunocompromised birds kept in poor conditions.

References

1) The references are extensive and relevant to the field. However, some are quite old (e.g., 1975, 1978). While citing classic papers is fine, the discussion should be balanced with more recent literature (post-2020) on the C. parapsilosis complex.

Language and Writing Style

1) "unpretentiousness to the substrate" -> "undemanding nutritional requirements"

2) "an unpleasant sticky body crumpled down" -> This phrase in the medical history is nonsensical and needs clarification.

3) The sentence structure is often overly complex and repetitive.

Reviewer #3: Dear authors

thank you very much for this article.

I have several suggestion, questions, and corrections in the pdf file.

How can you be sure that C. metapsilosis was the only causative agent? You mention that you also isolated other fungi.

You should review the CLSI document; Figure 7 does not comply with the guidelines set forth in the documents.

Is it possible to construct a phylogenetic tree? The percentages for the two strains are too low to be considered the same species (metapsilosis).

Several other comments in the file.

The figures are of very poor quality. They appear blurry.

kind regards

Reviewer #4: The manuscript adds to knowledge of what we know about the role of the yeast-like fungus Candida metapsilosis in the development of pododermatitis in steppe eagles in Kazakhstan. However, the authors can improve the quality of their write up in the conclusion section. By summarizing their key findings. And removing some sections such as (line 428 to 433, page 21. This section could be part of the discussion in the manuscript.

**********

6. PLOS authors have the option to publish the peer review history of their article (what does this mean?). If published, this will include your full peer review and any attached files.

If you choose “no”, your identity will remain anonymous but your review may still be made public.

**Do you want your identity to be public for this peer review?**  For information about this choice, including consent withdrawal, please see our Privacy Policy.

Reviewer #1: No

Reviewer #2: No

Reviewer #3: No

Reviewer #4: No

**********

[NOTE: If reviewer comments were submitted as an attachment file, they will be attached to this email and accessible via the submission site. Please log into your account, locate the manuscript record, and check for the action link "View Attachments". If this link does not appear, there are no attachment files.]

To ensure your figures meet our technical requirements, please review our figure guidelines: https://journals.plos.org/plosone/s/figures

You may also use PLOS’s free figure tool, NAAS, to help you prepare publication quality figures: https://journals.plos.org/plosone/s/figures#loc-tools-for-figure-preparation.

NAAS will assess whether your figures meet our technical requirements by comparing each figure against our figure specifications.

Attachment Submitted filename: PONE-D-26-07402_reviewer.pdf

# Author comment: Author response to Decision Letter 1

**DOI:** 10.1371/journal.pone.0353552.r002

24 Jun 2026

Response to Reviewer #1

We thank the reviewer for the positive assessment of our work and for the clear identification of the major issues that needed to be addressed before potential acceptance. We have carefully revised the manuscript according to all of the reviewer's comments.

Comment 1.1 - Methodology of biochemical analyses (API20 Candida / ID 32 C)

Reviewer #1: The MM for the biochemical analyses is lacking. We don't know how you performed them. Household approaches? API 20 Candida? ID 32 C?

Response: We agree that the original description was insufficient. We have clarified in the Materials and Methods section (subsection on biochemical characterization) that we used conventional in-house biochemical assays on individual classical media (Giss media for sugar fermentation, Christensen urea agar, meat-peptone gelatin, skim milk, peptone water, blood and keratin-enriched Sabouraud agar) rather than commercial identification panels such as API 20C AUX or ID 32 C (bioMérieux), which were not available in our laboratory. We explicitly state that definitive species-level identification therefore relied on ITS rDNA sequencing, while the biochemical assays were used to characterize the metabolic and pathogenicity-related phenotypes of the isolates. (Materials and Methods, Biochemical characterization subsection.)

Comment 1.2 - Short ITS sequences and absence of forward/reverse sequencing

Reviewer #1: Some of your ITS sequences are very short, too short for a successful sequencing. A correct ITS sequencing is supposed to give between 700/750 to 850/900 bp. Not around 250. Furthermore you do not explain if you have only a forward, reverse or both sequencing.

Response: We thank the reviewer for identifying this critical issue. We acknowledge that the originally submitted short sequences were inadequate for species-level identification. We have therefore repeated the entire molecular workflow: DNA was re-extracted, the ITS1–5.8S–ITS2 region was re-amplified, and the amplicons were sequenced bidirectionally using both the ITS1 (forward) and ITS4 (reverse) primers; consensus sequences were assembled from both reads. The new sequences have been re-deposited in GenBank under the following accession numbers: PV945930.1 (416 bp), PV945931.1 (413 bp) and PV981955.2 (467 bp). The corresponding electrophoresis image illustrating the proper amplicon length is included in the manuscript. The Materials and Methods section now explicitly describes the bidirectional sequencing strategy and the consensus assembly step. (Materials and Methods, Molecular identification subsection; Results; Discussion.)

Comment 1.3 - BLAST identity and species identification

Reviewer #1: PV981955 has a length of 239 bp, an E-value which is not 0.0 and an identity of 95.44%. Below 98 (or 99% if we want to be conservative) it is unlikely to be the correct species blasted. The same can be said for PV945931.1 (96.13% identity).

Response: We fully agree with the reviewer. Following the re-sequencing described in our response to comment 1.2, the new ITS sequences now meet conservative criteria for species-level identification within the C. parapsilosis complex. The updated BLAST results show: PV945930.1 (416 bp, bit score 769, E = 0.0), PV945931.1 (413 bp, bit score 763, E = 0.0), and PV981955.2 (467 bp, bit score 863, E = 0.0). These values are reported in the revised Results and Discussion sections, replacing the previous problematic data. Identification was further confirmed by phylogenetic analysis, in which all three isolates clustered within the C. metapsilosis clade together with reference sequences (see new Fig 6 and response to Reviewer #3).

Comment 1.4 - Figure 7 / CLSI antifungal susceptibility testing

Reviewer #1: Figure 7: this is not a CLSI result. Either you use antifungal discs (not a golden standard method) or microdilution method (CLSI – golden laboratory standard). I'd advise to present CLSI results.

Response: We thank the reviewer for this important methodological point. We have clarified in the Materials and Methods that we used disk diffusion in accordance with CLSI document M44-A2 (Mueller–Hinton agar supplemented with 2% glucose and 0.5 μg/mL methylene blue, inoculum adjusted to a 0.5 McFarland standard, incubation at 35 ± 2 °C for 24 h). We have also explicitly acknowledged the limitations of M44 in our setting: validated zone-diameter breakpoints are currently provided only for fluconazole and voriconazole against a limited number of Candida species, and no species-specific clinical breakpoints are available for C. metapsilosis or for nystatin, clotrimazole, ketoconazole and amphotericin B. Categorical thresholds previously applied to disk-diffusion testing of yeasts in veterinary studies were therefore used (≥20 mm = S, 15–19 mm = I/SDD, <15 mm = R). We now explicitly state that the present results constitute a screening characterization rather than a formal clinical susceptibility categorization, and that broth microdilution according to CLSI M27 (reference standard for yeasts) will be performed in a follow-up study to determine MIC values. The non-standard terminology ("weakly sensitive") has been replaced with the standard CLSI categories throughout the manuscript.

Response to Reviewer #2

We thank the reviewer for the very detailed and constructive comments, particularly regarding the methodology, the BLAST analysis and the framing of the Discussion. We have addressed every point and we believe the manuscript is substantially stronger as a result.

Comment 2.1 - Vague biochemical methodology; hair perforation test quantification

Reviewer #2: For the keratinophilic activity (hair perforation test), which specific protocol was used? Was it in vitro? How was it quantified to be deemed "high"?

Response: We have substantially expanded the description of the hair perforation test. The method is based on the protocol of Ajello (1967) and was performed in vitro on autoclaved hair fragments incubated with yeast suspensions on yeast-extract-supplemented water agar. For each isolate, at least 30 hair fragments were examined microscopically at ×400 magnification on days 7, 14, 21 and 30 of incubation, and keratinolytic activity was scored semi-quantitatively as follows: negative (no perforations), low (perforations on ≤25% of fragments, single perforations per hair), moderate (26–50%), and high (>50%, with multiple perforation sites per hair). All assays were performed in duplicate, and isolates were classified as "high" only when both replicates met this criterion. (Materials and Methods, Keratinolytic activity subsection.)

Comment 2.2 - CLSI standards (M27 vs M38 vs M44); breakpoints terminology

Reviewer #2: The specific CLSI standards (e.g., M27 for yeasts, not M38) must be correctly cited. The authors cite M38-A2 and M44-A2. M44 is for disk diffusion, but the results are presented in a way that suggests a dilution method. Which method was actually used, and how were the breakpoints determined? "Weakly sensitive" is not standard CLSI terminology for yeasts.

Response: We thank the reviewer for highlighting this confusion. The reference to M38-A2 (which is for filamentous fungi) was an error and has been removed. The method actually used was disk diffusion in accordance with CLSI M44-A2 (full protocol detailed in the response to Reviewer #1, comment 1.4). We have also replaced the non-standard "weakly sensitive" terminology with the standard CLSI categorical interpretation (S / I / SDD / R). We explicitly note in the manuscript that species-specific clinical breakpoints are not available for C. metapsilosis or for several of the antifungals tested, and that the present results should therefore be considered a screening characterization. Broth microdilution according to CLSI M27 will be performed in a follow-up study.

Comment 2.3 - BLAST comparison to C. parapsilosis instead of C. metapsilosis reference strains

Reviewer #2: Why compare to a C. parapsilosis sequence if you have identified it as C. metapsilosis? A direct comparison to confirmed C. metapsilosis reference strains (e.g., ATCC 96144) or other GenBank accessions would be more appropriate. The varying query coverage (750 bp vs 669 bp vs 442 bp) is not explained.

Response: We fully agree with the reviewer and we apologize for the confusion. We have revised the BLAST comparison to use C. metapsilosis reference sequences only, with the type-strain-derived sequence PV416737.1 (isolated from cattle, Italy) used as the primary reference. Additional C. metapsilosis reference sequences from animal sources (MF797776.1, MZ540270.1), environmental sources (JQ665419.1, FJ515169.1, FJ515199.1, FJ515200.1, QR731586.1) and human clinical sources (MH715400.1, PQ626156.1, MK561031.1, KU200445.1, KJ816926.1, KJ816917.1, KJ816927.1) were also compared. The C. parapsilosis sequence (KC777375.1) has been removed from this comparison. The varying query coverage in the previous version is no longer relevant, because after re-sequencing all three isolates yielded ITS amplicons of comparable, appropriate length (416, 413 and 467 bp), and the consensus sequences are based on bidirectional reads with full coverage.

Comment 2.4 - Overstated discussion of "uniqueness"

Reviewer #2: Phrases like "the uniqueness of C. metapsilosis isolation… explains the difficulties in diagnosis" are speculative.

Response: We agree. The corresponding paragraph has been substantially rewritten to remove speculative wording. The current text reads (Discussion): "The first reported case of C. metapsilosis isolation from wild eagles of the Kazakh population kept in long-term captivity is consistent with the clinical picture observed in this case: the attending veterinarians initially treated the lesions as bacterial pododermatitis and did not prescribe antifungal therapy, which may have contributed to the chronic course of the disease. … we therefore suggest, rather than conclude, that broader use of mycological work-up in non-resolving cases could be useful." The original speculative claim has been removed.

Comment 2.5 - Opportunistic pathogen vs. primary cause

Reviewer #2: Were these eagles sick because of C. metapsilosis, or was C. metapsilosis present because the eagles were sick and immunosuppressed? The wet bedding, poor nutrition, and stress are classic predisposing factors. The discussion must acknowledge that the fungus is likely an opportunistic pathogen exploiting a compromised host and environment, rather than the primary cause.

Response: This is a very important point and we fully agree. The Discussion has been substantially restructured to explicitly frame C. metapsilosis as an opportunistic pathogen. The relevant text now reads: "…damp litter, poor nutrition, and stress are classic factors predisposing to opportunistic infections. Given that the fungus C. metapsilosis is an opportunistic pathogen that exploits vulnerabilities in the host and the environment, it was the cause of the yeast mycosis, which developed as a secondary infection when favorable conditions arose…" In a new Discussion paragraph we also state that the keratinolytic activity, thermotolerance and (in one strain) pseudohyphal formation of these isolates are "consistent with an opportunistic pathogen that can play a role in the chronic progression of pododermatitis in immunocompromised birds kept under poor husbandry conditions, rather than with a primary, obligate pathogen". The terminology "causative agent" has been replaced throughout with "contributory aetiological role" or "opportunistic fungal contributor".

Comment 2.6 - Pseudohyphae attributed to genetic differences without WGS

Reviewer #2: The authors attribute pseudohyphae formation to "genetic differences," but without whole-genome sequencing, this is pure speculation.

Response: We agree and we have substantially softened this discussion. The relevant text now explicitly acknowledges the absence of whole-genome sequencing: "This may be related to strain-specific differences among the analyzed isolates; however, in the absence of whole-genome sequencing we cannot rigorously establish a genetic basis for this phenotype, and we therefore present this observation as a working hypothesis rather than a firm conclusion. Although all isolates were subcultured under nominally identical conditions… we cannot fully exclude an effect of the in vitro micro-environment at the time of subculture…" We have also added that whole-genome sequencing and comparative transcriptomics of the three isolates are planned in our future work to clarify this point.

Comment 2.7 - Absence of histopathology

Reviewer #2: No histopathology was performed in this study, which is a major limitation that should be mentioned.

Response: We fully agree and we have addressed this in two places. First, in the dedicated Limitations section, where "Absence of histological pathology" is explicitly listed. Second, in a new dedicated Discussion paragraph, we now state: "A major limitation of the present work, which we underline here in addition to the limitations listed below, is the absence of histopathology of the affected footpad tissue, which would have allowed direct visualization of fungal elements within the lesions and a more rigorous assessment of tissue invasion versus surface colonization; histopathological correlation should therefore be a priority in any future study of fungal pododermatitis in raptors."

Response to Reviewer #3

We thank the reviewer for the constructive comments and suggestions. The construction of a phylogenetic tree, in particular, has substantially strengthened the species identification, and the additional discussion of other co-isolated fungi has made the case for C. metapsilosis as the principal fungal contributor more rigorous.

Comment 3.1 - Was C. metapsilosis the only causative agent?

Reviewer #3: How can you be sure that C. metapsilosis was the only causative agent? You mention that you also isolated other fungi.

Response: This is a very important point. We have added a dedicated Discussion paragraph that critically addresses this question. We explain that the other filamentous fungi recovered from the primary cultures (Fusarium spp., Penicillium spp., Mucor spp. and Aspergillus spp., Fig 4) are common environmental moulds, and their isolation most likely reflects contamination of the wet, soiled bedding rather than an aetiological role in the foot lesions. Four lines of evidence support this interpretation: (i) these moulds were not recovered consistently from all three birds; (ii) they did not dominate the subcultures from the deep tissue scrapings; (iii) on subculture from the affected footpad tissue, only Candida-like colonies were repeatedly and abundantly recovered, with similar morphology in all three eagles; and (iv) only the Candida isolates combined the in vitro features most relevant to footpad pathology (thermotolerance up to 37 °C, urease activity and clear keratinolytic activity in the hair-perforation assay). Together with the clinical response to topical antimycotic therapy, these observations support C. metapsilosis as "the most likely fungal contributor to the chronic lesions, although we cannot formally exclude an additional, transient role of other environmental moulds". The wording has been deliberately chosen to reflect the appropriate level of certainty.

Comment 3.2 - Figure 7 does not comply with CLSI guidelines

Reviewer #3: You should review the CLSI document; Figure 7 does not comply with the guidelines set forth in the documents.

Response: Thank you for this comment, which echoes the concerns raised by Reviewers #1 and #2. The Materials and Methods section has been revised to explicitly state the use of the CLSI M44-A2 disk-diffusion protocol, to use standard CLSI categorical terminology (S / I / SDD / R), and to acknowledge the methodological limitations (absence of species-specific clinical breakpoints for C. metapsilosis and for most of the antifungals tested). The figure caption and the corresponding Results paragraph have been updated accordingly. (See response to Reviewer #1, comment 1.4, for the full description.)

Comment 3.3 - Phylogenetic tree; low identity percentages for two strains

Reviewer #3: Is it possible to construct a phylogenetic tree? The percentages for the

Attachment Submitted filename: Response_to_Reviewers.docx

# Editor report: Decision Letter 1

**DOI:** 10.1371/journal.pone.0353552.r003

**Authors:** Petr Heneberg

25 Jun 2026

Isolation and characterization of Candida metapsilosis from foci of chronic pododermatitis in captive steppe eagles in Kazakhstan

PONE-D-26-07402R1

Dear Dr. Nessipbayeva,

We’re pleased to inform you that your manuscript has been judged scientifically suitable for publication and will be formally accepted for publication once it meets all outstanding technical requirements.

Within one week, you’ll receive an e-mail detailing the required amendments. When these have been addressed, you’ll receive a formal acceptance letter and your manuscript will be scheduled for publication.

An invoice will be generated when your article is formally accepted. Please note, if your institution has a publishing partnership with PLOS and your article meets the relevant criteria, all or part of your publication costs will be covered. Please make sure your user information is up-to-date by logging into Editorial Manager at Editorial Manager® and clicking the ‘Update My Information' link at the top of the page. For questions related to billing, please contact billing support.

If your institution or institutions have a press office, please notify them about your upcoming paper to help maximize its impact. If they’ll be preparing press materials, please inform our press team as soon as possible -- no later than 48 hours after receiving the formal acceptance. Your manuscript will remain under strict press embargo until 2 pm Eastern Time on the date of publication. For more information, please contact onepress@plos.org.

Kind regards,

Petr Heneberg

Academic Editor

PLOS One

Additional Editor Comments (optional):

Reviewers' comments:

# Editor report: Acceptance letter

**DOI:** 10.1371/journal.pone.0353552.r004

**Authors:** Petr Heneberg

PONE-D-26-07402R1

PLOS One

Dear Dr. Nessipbayeva,

I'm pleased to inform you that your manuscript has been deemed suitable for publication in PLOS One. Congratulations! Your manuscript is now being handed over to our production team.

At this stage, our production department will prepare your paper for publication. This includes ensuring the following:

* All references, tables, and figures are properly cited

* All relevant supporting information is included in the manuscript submission,

* There are no issues that prevent the paper from being properly typeset

You will receive further instructions from the production team, including instructions on how to review your proof when it is ready. Please keep in mind that we are working through a large volume of accepted articles, so please give us a few days to review your paper and let you know the next and final steps.

Lastly, if your institution or institutions have a press office, please let them know about your upcoming paper now to help maximize its impact. If they'll be preparing press materials, please inform our press team within the next 48 hours. Your manuscript will remain under strict press embargo until 2 pm Eastern Time on the date of publication. For more information, please contact onepress@plos.org.

You will receive an invoice from PLOS for your publication fee after your manuscript has reached the completed accept phase. If you receive an email requesting payment before acceptance or for any other service, this may be a phishing scheme. Learn how to identify phishing emails and protect your accounts at https://explore.plos.org/phishing.

If we can help with anything else, please email us at customercare@plos.org.

Thank you for submitting your work to PLOS ONE and supporting open access.

Kind regards,

PLOS ONE Editorial Office Staff

on behalf of

Dr. Petr Heneberg

Academic Editor

PLOS One
